# ﻿Mid-Holocene marine faunas from the Bangkok Clay deposits in Nakhon Nayok, the Central Plain of Thailand

**DOI:** 10.3897/zookeys.1202.119389

**Published:** 2024-05-15

**Authors:** Parin Jirapatrasilp, Gilles Cuny, László Kocsis, Chirasak Sutcharit, Nom Ngamnisai, Thasinee Charoentitirat, Satapat Kumpitak, Kantapon Suraprasit

**Affiliations:** 1 Animal Systematics Research Unit, Department of Biology, Faculty of Science, Chulalongkorn University, Bangkok 10330, Thailand; 2 Leibniz-Institut zur Analyse des Biodiversitätswandels - Standort Hamburg, Martin-Luther-King-Platz 3, Hamburg 20146, Germany; 3 Université Claude Bernard Lyon 1, LEHNA UMR 5023, CNRS, ENTPE, F-69622, Villeurbanne, France; 4 Institute of Earth Surface Dynamics, University of Lausanne, Rue de la Mouline, 1015 Lausanne, Switzerland; 5 Department of Geography, Faculty of Social Sciences, Srinakharinwirot University, Bangkok 10110, Thailand; 6 Department of Geology, Faculty of Science, Chulalongkorn University, Bangkok 10330, Thailand; 7 Center of Excellence in Morphology of Earth Surface and Advanced Geohazards in Southeast Asia (MESA CE), Department of Geology, Faculty of Science, Chulalongkorn University, Bangkok 10330, Thailand

**Keywords:** carbon-14 dating, Chao Phraya River Basin, Chondrichthyes, marine transgression, mollusc, paleoenvironment

## Abstract

Based on several field investigations, many molluscan shells and chondrichthyan teeth, together with other invertebrate and actinopterygian remains were found from the marine Bangkok Clay deposits in Ongkharak, Nakhon Nayok, at a depth of ~ 5–7 m below the topsoil surface. Animal macrofossils recovered from these Holocene marine deposits were identified and their chronological context was investigated in order to reconstruct the paleoenvironments of the area at that time. The majority of marine fossils recovered from the site consist of molluscs, with a total of 63 species identified. Other invertebrate species include a stony coral, a mud lobster, barnacles, and a sea urchin. The vertebrates are represented by fish remains, including carcharhinid shark teeth from at least nine species, stingray and trichiurid teeth, and one sciaenid otolith. The molluscan fauna indicates that the paleoenvironments of the area corresponded to intertidal to sublittoral zones, where some areas were mangrove forests and intertidal mudflats. The fish fauna is dominated by the river shark *Glyphis*, indicating freshwater influences and possibly occasional brackish conditions. The carbon-14 analysis of mollusc and charcoal remains shows that deposition of the marine sediment sequence began during the mid-Holocene, spanning approximately from 8,800 to 5,300 cal yr BP. This study provides in-depth insights into the diversity of fishes, marine molluscs, and other invertebrates from the Bangkok Clay deposits, supporting the existence of a marine transgression onto the Lower Central Plain of Thailand during the mid-Holocene.

## ﻿﻿Introduction

The Holocene began approximately 11,700 years ago and belongs to one of the interglacial periods. Prior to the Holocene, during the period of the Last Glacial Maximum (LGM), the global climate was much colder and dryer and was a period of significant landscape changes in many areas due to a lowered sea level, approximately 120 m below the present-day stands (Clark et al. 2009). During the Holocene, however, the climate became warmer, and the sea level rose and inundated a previously emerged landmass. Southeast Asia is one of the regions impacted by sea level inundations (Sathiamurthy and Voris 2006), due in part to an increase in precipitation level after the cold and dry period (Chabangborn 2017). Differences in climatic and environmental conditions between the Pleistocene and Holocene have therefore had impacts on the ecological adaptations of both terrestrial and marine organisms, leading to dispersal, migration, and extinction of species.

The Central Plain of Thailand is an important area where the topography and climate changes were under the influence of a sea level rise starting at ~ 12 ka. The region covers the plains between the mountain ranges along the lower northern part of Thailand, including the Yom and Nan River basins, and the low-lying plains of the Chao Phraya, Tha Chin, Mae Klong and Bang Pakong River basins near the Gulf of Thailand (Sinsakul 2000). During the Holocene, the Sing Buri Plain (5–15 m above mean sea level (amsl)) and the Bangkok Lowland (0–5 m amsl), forming the Central plain of Thailand, were inundated (Takaya 1969). Approximately 8–7 ka, the sea level was 2–4 m higher than present-day stands and inundated the whole lowland areas of Bangkok. Later, the sea level subsided along with a new sedimentation regime and a new landmass started to develop, shaping the current coastal shorelines (Umitsu et al. 2002; Tanabe et al. 2003).

The extent of marine intrusion into the Central Plain of Thailand during the Holocene was largely interpreted based on various studies (e.g., geomorphological, sedimentological, paleontological, palynological, and stratigraphical data as well as carbon-14 dating of peat layers). These studies indicated that the ancient coastal shoreline of the Gulf of Thailand was located further north in Phra Nakhon Si Ayutthaya Province during 7.3–6.5 ka (Jarupongsakul and Thiramongkol 1992). The ancient coastal shoreline was also reconstructed based on a 4,500-year-old coastal archaeological site in Chonburi Province (Boyd et al. 1996), open-pit mining of clay deposits at a depth of 7 m under the ground surface in many provinces, dated to ~ 8–7 ka (Songtham et al. 2007, 2015), and geological studies of coastal berms in Chumporn and Prachuap Khiri Khan provinces, dated between 8.9–5.6 ka and 6.5–6 ka, respectively (Nimnate et al. 2015; Surakiatchai et al. 2018).

In addition to the inference of ancient coastal shorelines during the Holocene, studies on faunas and floras along the ancient coast of the Gulf of Thailand are crucial to understand paleoenvironmental and palaeoecological changes in response to climatic oscillations. The ancient coastal habitats occupied by these animals are investigated based on the identification and dating of fossils (e.g., molluscs, arthropods, and vertebrates). According to accurate species-level identification, microhabitats can be investigated based on comparisons between fossils and living species or related taxa. The paleoenvironments of the area are also reconstructed based on the identification of various groups of animals because each of them has a different mode of life in a specific habitat (Negri 2009, 2012; Oliver and Terry 2019).

**Figure 1.**
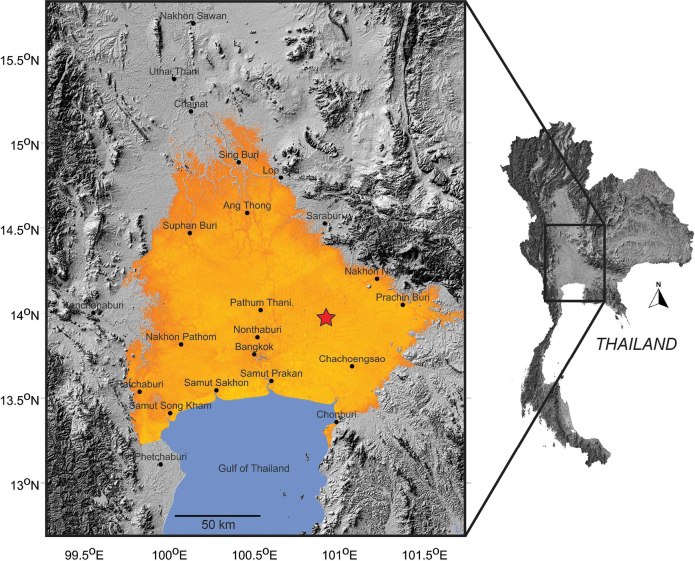
Map of the lower central plain of Thailand (orange). A red star indicates the location of the study area.

Marine faunas from the Holocene Bangkok Clay deposits of the Central Plain of Thailand have infrequently been analysed in detail, and we identify the animal macrofossils recovered from the clay pit of Ongkharak, Nakhon Nayok Province in central Thailand (Fig. 1). We also date the Bangkok Clay sequence deposited in the area, using a carbon-14 dating technique. Although the Bangkok Clay deposits have previously been described from some areas of Nakhon Nayok Province (Songtham et al. 2007; Royal Irrigation Department 2009), the fauna and its paleoenvironments have not yet been thoroughly studied and compared to other sites from different regions (e.g., Negri 2009, 2012). This study not only fulfils the gap regarding the paleoenvironments of the Gulf of Thailand during the Holocene, but also illustrates the ancient coastlines in the eastern part of the Central Plain of Thailand to some extent. The reconstruction of ancient coastlines and paleoenvironments is crucial to advance the knowledge of archaeological contexts in terms of prehistoric human settlements and cultures, and probably helpful in forecasting changes in coastal ecosystems and environments in response to sea level oscillations due to the present-day global warming situation.

## ﻿﻿Geological settings of the study area

The study area is located at the clay pit in Ban Lad Chang, Chumpon Subdistrict, Ongkharak District, Nakhon Nayok Province (13°59'30.9"N, 100°55'11.5"E) in the Lower Central Plain of Thailand (Fig. 1), which is covered by the thick marine deposits of the Chao Phraya River delta, also known as the Bangkok Clay. These marine clay deposits have been dated between the Late Pleistocene and Holocene (Cox 1968; Moh et al. 1969; Sinsakul 2000). The Bangkok clay sequence comprises three main zones (Ohtsubo et al. 2000): 1) weathered clay with a thickness of 0–2 m in the uppermost part, 2) soft marine clay in the middle part, and 3) medium stiff clay at the bottom. The Holocene Bangkok Soft Clay in the middle part, with a thickness ranging from 10 to 20 m, is olive grey and medium to dark grey in colour, indicating reducing environments (Sinsakul 2000; Choowong 2002; Tanabe et al. 2003). This Bangkok Soft Clay layer is further divided into lower transgressive peaty (mangrove swamp) and upper regressive deltaic sediments (Tanabe et al. 2003). Some marine shells and remains of mangrove trees were found from this middle layer, indicating intertidal and shallow sublittoral inner bay conditions, with local mangrove and freshwater influences (Robba et al. 2002, 2003, 2004, 2007; Songtham et al. 2007; Negri 2009, 2012). This evidence supports the occurrence of a marine transgression into the Central Plain of Thailand after the LGM (Songtham et al. 2015).

**Figure 2.**
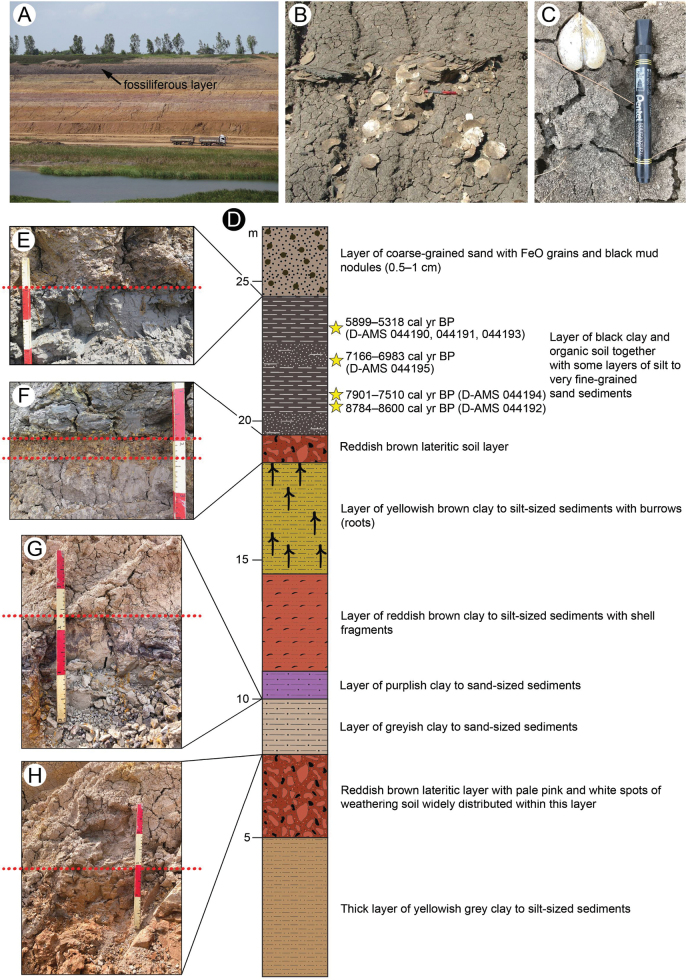
The study area and the sediment profile of the clay pit **A** the clay pit of Ongkharak, Nakhon Nayok Province in central Thailand **B** the Bangkok clay layer bearing numerous *in situ* complete shells of *Placunaplacenta***C** Shells of a bivalve *Anadarainaequivalvis* buried *in situ* in the Bangkok clay deposits **D** lithological profile of the clay pit **E** the contact between two successive layers: coarse-grained sand (upper) and black clay (lower) **F** the contact between three successive layers: black clay (upper), lateritic soil (middle), and yellowish brown clay to silt (lower) **G** the contact between two successive layers: purplish (upper) and greyish (lower) clay to sand **H** the contact between two successive layers: greyish clay to sand (upper) and reddish brown lateritic layer (lower).

The rectangular mine is 1,000 m long × 400 m wide and 30 m deep. It was opened to produce clay material used in construction sites. The active mining operations of the clay pit allow us to have access to the stratigraphic sequence of the Quaternary deposits of the area (Fig. 2). The topsoil (0.5–1 m thick) consists of coarse-grained sand with FeO grains and black mud nodules, underlain by a 5 m thick layer of black clay and organic soil (dark layer) partially interbedded with thin layers of silt to very fine-grained sand. Marine invertebrate and vertebrate remains were only found from this organic-rich clay unit, also known as the Bangkok Clay, which overlies the layers of Pleistocene stiff clay (Rau and Nutalaya 1983). In this site, the Pleistocene stiff clay contains seven different lithological units from the top to bottom of the pit: 1) reddish brown lateritic soil (1 m thick), 2) yellowish brown clay to silt-sized sediments with root-trace burrows (4 m thick), 3) reddish brown clay to silt-sized sediments with shell fragments (4 m thick), 4) purplish clay to sand-sized sediments (1 m thick), 5) greyish clay to sand-sized sediments (2 m thick), 6) reddish brown lateritic soil with pale pink and white spots of weathering soil (3 m thick), and 7) yellowish grey clay to silt-sized sediments (5 m thick), respectively (Fig. 2).

The Bangkok clay layer at the clay pit of Ongkharak has yielded nearly *in situ* fossil deposits as indicated by preservation of complete shells with both valves attached (Fig. 2C) as well as the orientation of shell valves of *Pholasorientalis*, which has been recovered in natural position within its burrows during field collection (see also Songtham et al. 2007: fig. 3). Although remains of marine molluscs were found throughout the sequence of dark clay deposits, they were most abundant in the middle part of the layer (Fig. 2D–F), at a depth of ~ 2 m below the upper contact that separates it from the topsoil. Numerous wood fragments, plant remains, and giant oyster shells were found from the lower part of the dark clay unit below the shell-rich layer.

## ﻿﻿Materials and methods

Species identification of specimens is mainly based on the existing literature specified under the treatment of each taxon. The specimens are kept at the Department of Geology, Faculty of Science, Chulalongkorn University, and coded as the name of the collection (**CUF**: Chulalongkorn fossil collection), followed by the locality (**NKNY**: Nakhon Nayok) and the catalogue number. Fish remains including teeth and otolith as well as some small shells were photographed with a scanning electron microscope (SEM; JEOL, JSM-5410 LV) or a Leica M205C stereo light microscope with fusion optics and the Leica Application Suite Image System. L and R designate left and right valves of bivalve shells, respectively.

We selected six samples (shells and charcoal) collected from the marine clay deposits at different depths in the clay pit for carbon-14 dating (Table 1). The samples were pretreated and analysed at the DirectAMS (USA), using an accelerated mass spectrometer (AMS). According to the convention outlined by Stuiver and Polach (1977), the ^14^C age ± one standard deviation (SD) was calculated using the Libby half-life of 5,568 years and an isotopic fractionation correction based on *δ*^13^C measurements obtained from the AMS. The DirectAMS results were calibrated and corrected for the marine reservoir effect, using the Calib 8.2 program with IntCal20.14c for charcoal (Reimer et al. 2020) and with Marine20 (Heaton et al. 2020) for shells.

**Table 1.** AMS^14^C ages of shells and charcoal collected from the clay pit of Ongkharak in Nakhon Nayok, central Thailand.

Lab Code	Sample type	Depths (m) below the uppermost part of a marine clay layer	^14^C age (yr BP)	Calibrated ^14^C (2-sigma) age (cal yr BP)
D-AMS 044190	Shell (*Tellinidesconspicuus*)	2.2	5303 ± 26	5882–5464
D-AMS 044191	Shell (*Pholasorientalis*)	2.2	5202 ± 28	5757–5318
D-AMS 044193	Charcoal	2.2	5047 ± 26	5899–5726
D-AMS 044195	Charcoal	3.4	6186 ± 29	7166–6983
D-AMS 044194	Shell (Magallanacf.gigas)	4	7241 ± 32	7901–7510
D-AMS 044192	Charcoal	4.6	7913 ± 26	8784–8600

## ﻿﻿Results

The ages of the Bangkok Clay deposits in the clay pit of Ongkharak range from 8,784 calibrated years before the present (cal yr BP) to 5,318 cal yr BP (mid-Holocene) based on the radiocarbon dating of several shell and charcoal fragments collected along the stratigraphic section of the layer (Table 1). The majority of marine faunas are molluscs, where a total of 63 species were identified, including 35 gastropod species from 20 families, 27 bivalve species from 15 families, and one scaphopod species. The most common gastropods are *Architectonicaperdix*, *Naticastellata*, and *Indothaislacera*, whereas the most common bivalves are *Corbulafortisulcata*, Magallanacf.gigas, and *Joannisiellaoblonga*. Other invertebrate remains include one species of stony corals (Oulangiacf.stokesiana), one unidentified species of mud lobsters (*Thalassina* sp.), two species of barnacles (*Fistulobalanuskondakovi* and Megabalanuscf.tintinnabulum), and one species of sea urchins (*Temnotremasiamense*).

The vertebrate remains include at least nine species (two families) of chondrichthyan fishes and at least two species (two families) of actinopterygian taxa. Altogether, 100 cartilaginous fish fossils were recovered, including 97 remains identified as belonging to the family Carcharhinidae and three to the family Dasyatidae. The teeth of the latter belong to the stingray genus *Pastinachus*. Among the carcharhinids, the genus *Glyphis* dominates the shark fauna (61%). There are also at least six species of *Carcharhinus*, representing 26% of the shark fauna. The accurate identification of *Carcharhinus* species is often hindered by the preservation of teeth or by the morphological similarities of tooth positions among these taxa. A few teeth among the carcharhinids belong to the genus *Scoliodon*. Few actinopterygian remains were also found, including three teeth that belong to the family Trichiuridae and one sciaenid otolith that represents the genus *Johnius*.

### ﻿﻿Systematic palaeontology

#### ﻿Phylum Cnidaria

﻿**Class Anthozoa Ehrenberg, 1834**

**Subclass Hexacorallia Haeckel, 1896**

**Order Scleractinia Bourne, 1900**

##### ﻿Family Rhizangiidae d’Orbigny, 1851

***Oulangia* Milne Edwards & Haime, 1848**

###### ﻿ Oulangia cf. stokesiana

Taxon classificationAnimaliaScleractiniaRhizangiidae

Milne Edwards & Haime, 1848

82A36A65-B8AD-5D71-B95C-D568CD552B02

Figs 3A , 5A

cf. Oulangia stokesiana Milne Edwards & Haime, 1848: pl. 7, fig. 4, 4a. Type locality: Philippines. Milne Edwards and Haime 1849: 183. Lam et al. 2008: 742, fig. 4e.
cf. Oulangia stokesiana stokesiana . Cairns et al. 1999: 39.

####### Referred material.

**Figure 3.**
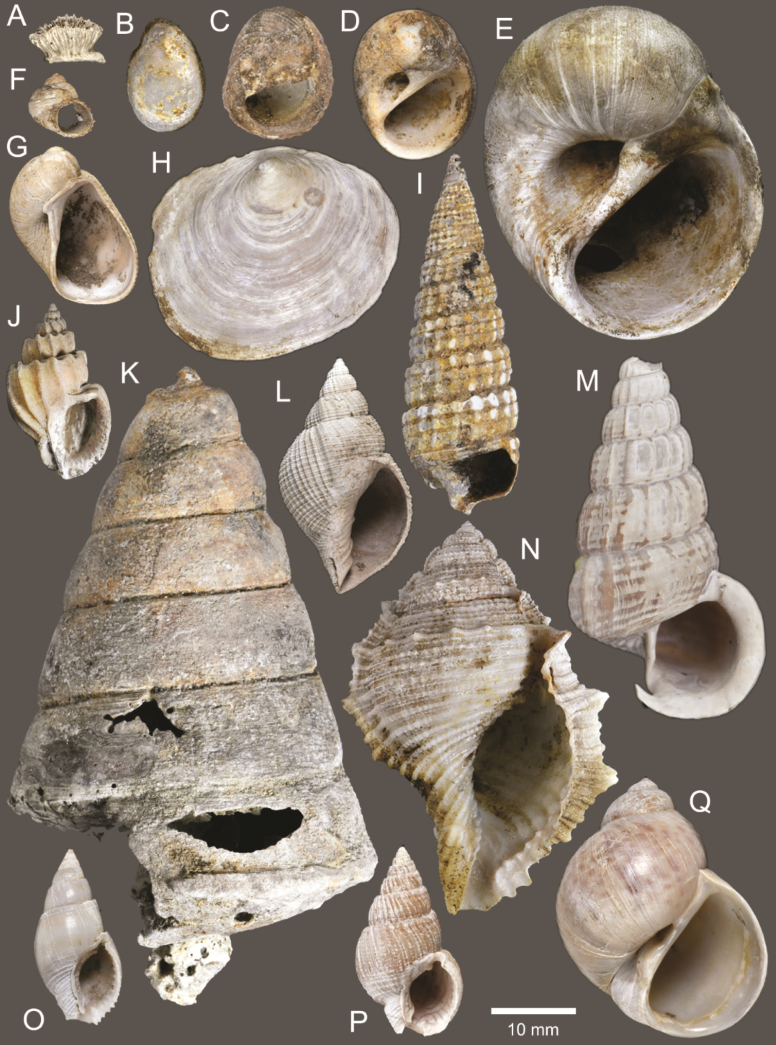
Size comparison of coral and gastropods found in this study **A**Oulangiacf.stokesiana**B***Neripteronviolaceum***C***Neritaarticulata***D***Naticastellata***E***Naticavitellus***F**Homalopomacf.sangarense**G***Eunaticinapapilla***H***Ergaeawalshi***I***Pirenellaincisa***J***Scalptiascalariformis***K***Telescopiumtelescopium***L***Mericaelegans***M***Cerithideaobtusa***N***Bufonariarana***O***Nassariusmicans***P***Nassariussiquijorensis***Q***Paratectonaticatigrina*.

CUF-NKNY-O07 (1 specimen; Figs 3A, 5A).

####### Habitat.

Shallow waters as well as in submarine caves (Lam et al. 2008).

####### Distribution.

Indian Ocean; Indo-West Pacific, from Japan to the Philippines, and central Pacific (Cairns et al. 1999; Lam et al. 2008).

####### Taxonomic remarks and comparisons.

This specimen is classified into the genus *Oulangia* based on the descriptions and figures of Cairns and Kitahara (2012) and Baron-Szabo and Cairns (2016). We tentatively identify this specimen as belonging to *O.stokesiana* according to Lam et al. (2008).

#### ﻿Phylum Mollusca

﻿**Class Gastropoda Cuvier, 1795**

**Subclass Vetigastropoda Salvini-Plawen, 1980**

**Order Trochida Cox & Knight, 1960**

**Superfamily Trochoidea Rafinesque, 1815**

##### ﻿Family Colloniidae Cossmann, 1917

***Homalopoma* Carpenter, 1864**

###### ﻿ Homalopoma cf. sangarense

Taxon classificationAnimaliaTrochidaColloniidae

(Schrenck, 1861)

25601CB2-3DD8-5417-A75C-447A704D810E

Figs 3F , 5B

cf. Turbo sangarensis Schrenck, 1861: 409–410. Type locality: the Sangaric strait near the shore of the island of Jesso [Sangar (Tsugaru) Strait, Hakodate Bay, Hokkaido, Japan].
cf. Homalopoma sangarense . Habe 1958: 5, pl. 2, fig. 1. Golikov and Kussakin 1978: 65–66, fig. 40a, b. Egorov 2000: 66, with in-text fig. Kantor and Sysoev 2006: 42, pl. 18h. Lebedev 2014: 57.

####### Referred material.

CUF-NKNY-G24 (1 shell; Figs 3F, 5B).

####### Habitat.

On silty-sandy or rarely on rocky substrates at a depth from 2 to 22 m (Egorov 2000; Kantor and Sysoev 2006).

####### Distribution.

Sea of Japan and southern Kuril Islands (Kantor and Sysoev 2006; Lebedev 2014).

####### Record in Thailand.

This is the first record of this species in Thailand.

####### Taxonomic remarks and comparisons.

Although there is only one incomplete specimen, it is tentatively identified as *H.sangarense* based on shell size and shape and several strong spiral cords on the surface as in the descriptions and figures in Habe (1958), Golikov and Kussakin (1978), and Egorov (2000).

**Subclass Neritimorpha Golikov & Starobogatov, 1975**

**Order Cycloneritida Frýda, 1998**

**Superfamily Neritoidea Rafinesque, 1815**

##### ﻿Family Neritidae Rafinesque, 1815

***Neripteron* Lesson, 1831**

###### ﻿ Neripteron violaceum

Taxon classificationAnimaliaCycloneritidaNeritidae

(Gmelin, 1791)

2574E2D3-BDFB-5185-8E6E-156DBC876B57

Figs 3B , 5C

**Figure 4.**
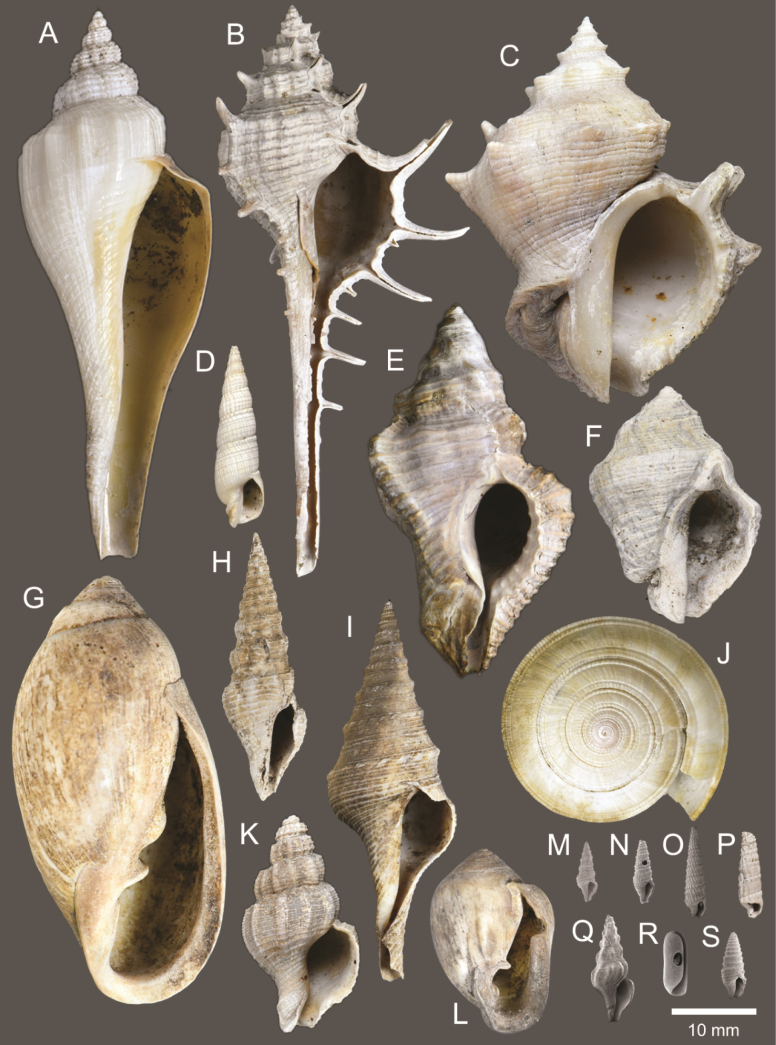
Size comparison of gastropods found in this study **A***Brunneifususternatanus***B***Murextrapa***C***Indothaislacera***D***Pristiterebramiranda***E***Chicoreuscapucinus***F***Indothaisgradata***G***Ellobiumaurisjudae***H***Inquisitorvulpionis***I***Turriculajavana***J***Architectonicaperdix***K***Pseudoneptuneavaricosa***L***Cassidulanucleus***M***Pseudoetremafortilirata***N***Paradrilliamelvilli***O***Duplicariatricincta***P***Granuliterebrabathyrhaphe***Q***Comitasilariae***R***Cylichnamodesta***S***Maoritomellavallata*.

Nerita violacea Gmelin, 1791: 3686. Type locality: unknown.
Neritina violacea . Tantanasiriwong 1978: 6, fig. 52. Nateewathana et al. 1981: 56. Wilson 1993: 41, pl. 2, figs 18a, b, 19a–d. Hylleberg and Kilburn 2003: 31. Robba et al. 2004: 24–25, pl. 2, fig. 5a, b. Robba et al. 2007: 89 (appendix). Printrakoon et al. 2008: table 1. Tan and Clements 2008: 490–491, fig. 3-33, 3-34. Nabhitabhata 2009: 39. Sanpanich and Duangdee 2013: 53. Ng et al. 2016: fig. 1-28. Yang et al. 2017: 20, fig. 75. Tudu et al. 2018: table 1.
Dostia violacea . Way and Purchon 1981: 314. Subba Rao and Dey 2000: 35. Sri-aroon et al. 2004: table 1, fig. 2-1. Dechruksa et al. 2014: fig. 2k.
Neripteron violacea . Swennen et al. 2001: 52, 108, text-fig. 275, fig. 275.
Neritina (Dostia) violacea . Gemert 2003: 103. Sri-aroon et al. 2005: tables 2, 3, 5, 6. Dey 2006: 22, figs 11, 12. Ramakrishna et al. 2007: 5, 37. Kesavan et al. 2009: 382, with in-text fig. Eichhorst 2016: 392–395, pl. 102, figs 1–15.
Neritina (Neripteron) violacea . Thach 2005: 38, pl. 6, figs 8, 9.
Neripteron violaceus . Eichhorst 2008: 268, pl. 79, fig. 7a, b.
Neripteron violaceum . Baharuddin et al. 2017: fig. 3o. BEDO 2017b: 97, with in-text fig. Kantharajan et al. 2017: table 1, fig. 4-34. Yadav et al. 2019: table 2, fig. 1h. Mustapha et al. 2021: 52–53, figs 3e, f, 4i. Wells et al. 2021: 154–155.

####### Referred material.

**Figure 5.**
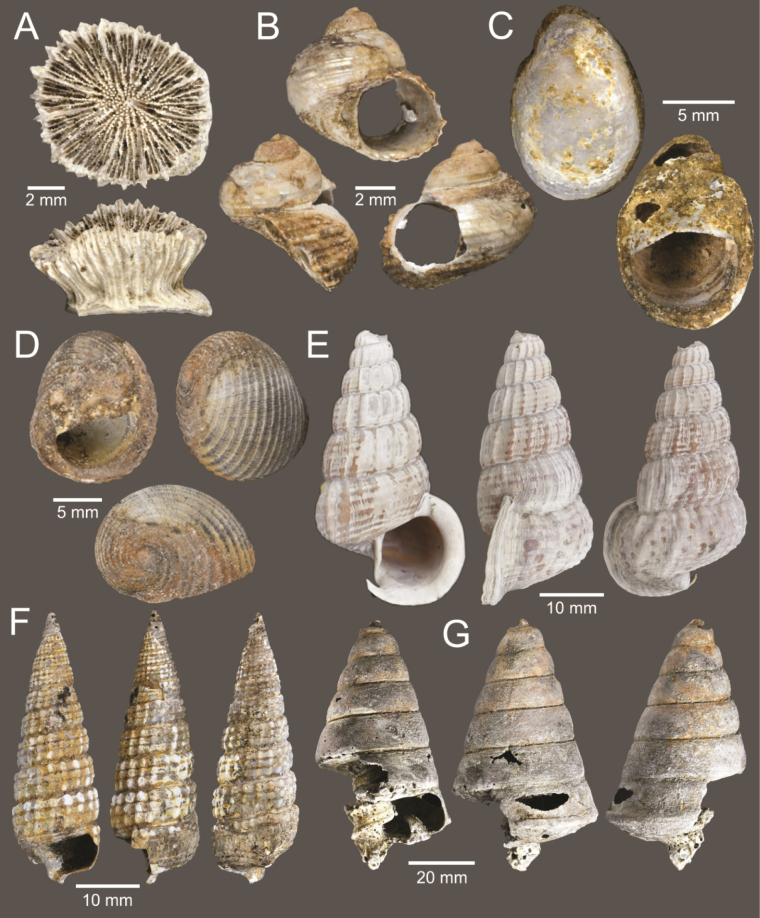
Coral and gastropods **A**Oulangiacf.stokesiana**B**Homalopomacf.sangarense**C***Neripteronviolaceum***D***Neritaarticulata***E***Cerithideaobtusa***F***Pirenellaincisa***G***Telescopiumtelescopium*.

CUF-NKNY-G26 (28 shells; Figs 3B, 5C).

####### Habitat.

On Nipa palms, muddy surfaces, old wood and rocks in brackish habitats and mangrove forests (Swennen et al. 2001; Mustapha et al. 2021) as well as on gravels in high tidal zones (Yang et al. 2017).

####### Distribution.

India (Kantharajan et al. 2017; Yadav et al. 2019); Indo-West Pacific, from Japan to Australia (Wilson 1993; Yang et al. 2017).

####### Record in Thailand.

Gulf of Thailand and Andaman Sea (Wells et al. 2021).

####### Taxonomic remarks and comparisons.

The shell of *N.violaceum* is highly similar to that of *N.cornucopia* (Benson, 1836) but differs in having a purple to orangish red ventral side, and denticulations along the columellar edge often only present in the central part (Huang 1997; Tan and Clements 2008). Although the columella edge of the specimens is corroded, there are still some traces of purple colour at the ventral side. Therefore, these specimens are identified as belonging to *N.violaceum*.

***Nerita* Linnaeus, 1758**

###### ﻿ Nerita articulata

Taxon classificationAnimaliaCycloneritidaNeritidae

Gould, 1847

43B4525F-79FC-5E67-A0F0-19D729587B4C

Figs 3C , 5D

Nerita lineata Gmelin, 1791: 3684 [junior homonym of Neritalineata Müller, 1774]. Type locality: Strait of Malacca. Way and Purchon 1981: 314. Hylleberg and Kilburn 2003: 29–30. Yang et al. 2017: 20, fig. 67.
Nerita (Ritena) lineata . Cernohorsky 1972: 51, pl. 11, fig. 5.
Nerita articulata Gould, 1847: 220. Type locality: Tavoy. Wium-Andersen 1977: 5, fig. 8. Tantanasiriwong 1978: 6, fig. 42. Nateewathana et al. 1981: 56. Tan and Clements 2008: 483–485, fig. 2-3, 2-4. Nabhitabhata 2009: 36. Hamli et al. 2013: tables 2, 3, fig. 2g. Tudu et al. 2018: table 1.
Nerita balteata Reeve, 1855: pl. 6, fig. 28. Type locality: unknown. Wilson 1993: 40, pl. 2, fig. 9a, b. Nateewathana 1995: 95, with in-text fig. Nabhitabhata 2009: 36. Sanpanich and Duangdee 2013: 53. Chen et al. 2015: 75, fig. 1a–c. BEDO 2017b: 99, with in-text fig. Kantharajan et al. 2017: table 1, fig. 4-36. Tudu et al. 2018: table 1. Raven 2019: figs 4–6. Yadav et al. 2019: table 2, fig. 1i. Mustapha et al. 2021: 48, figs 2m, n, 4g. Wells et al. 2021: 155.
Nerita (Ritena) balteata . Cernohorsky 1978: 42, pl. 11, fig. 1. Dharma 2005: 68, pl. 9, fig. 3a, b.
Nerita (Amphinerita) articulata . Subba Rao and Dey 2000: 31. Dey 2006: 20, figs 7, 8.
Nerita (Nerita) articulata . Sri-aroon et al. 2004: table 1.
Nerita (Theliostyla) balteata . Thach 2005: 37, pl. 6, figs 36, 37.
Nerita balteata forma articulata . Eichhorst 2008: 280, pl. 85, fig. 1a, b.
Nerita (Cymostyla) balteata . Eichhorst 2016: 455–457, pl. 113, figs 1–9.

####### Referred material.

CUF-NKNY-G61 (3 shells; Figs 3C, 5D).

####### Habitat.

On tree trunks, branches, roots and on muddy banks and rocky areas or near mangrove forests (Tan and Clements 2008; Mustapha et al. 2021) as well as on rocks in intertidal zones (Yang et al. 2017).

####### Distribution.

India (Kantharajan et al. 2017; Yadav et al. 2019); Indo-West Pacific, from China to Australia (Wilson 1993; Yang et al. 2017). Also reported from the Hawaiian Islands (Cernohorsky 1978).

####### Record in Thailand.

Gulf of Thailand and Andaman Sea (Wells et al. 2021).

####### Taxonomic remarks and comparisons.

This species is recognised by numerous raised spiral cords and a crenulated outer lip (Tan and Clements 2008). Although this species is widely known as *N.balteata* Reeve, 1855, the name *N.articulata* was made available earlier by Gould (1847). Thus, it has a priority over *N.balteata*.

**Subclass Caenogastropoda Cox, 1960**

**Cohort Sorbeoconcha Ponder & Lindberg, 1997**

**Subcohort Cerithiimorpha Golikov & Starobogatov, 1975**

**Superfamily Cerithioidea Fleming, 1822**

##### ﻿Family Potamididae H. Adams & A. Adams, 1854

***Cerithidea* Swainson, 1840**

###### ﻿ Cerithidea obtusa

Taxon classificationAnimaliaCycloneritidaPotamididae

(Lamarck, 1822)

05E8E6F8-ABB7-50FF-A1CC-6987CDD28ADB

Figs 3M , 5E

Cerithium obtusum Lamarck, 1822: 71. Type locality: Timor.
Cerithidea obtusa . Cernohorsky 1972: 60 (in part). Tantanasiriwong 1978: 7, fig. 70. Nateewathana et al. 1981: 57. Way and Purchon 1981: 315. Nateewathana 1995: 97, with in-text fig. Poutiers 1998b: 450, with in-text figs. Gemert 2003: 104. Hylleberg and Kilburn 2003: 37–38. Robba et al. 2004: 31–32, pl. 3, fig. 4a, b. Dharma 2005: 92, pl. 21, fig. 8; 308, pl. 119, fig. 8a, b. Thach 2005: 49, pl. 10, fig. 4. Dey 2006: 35–36, figs 38–40. Robba et al. 2007: 89 (appendix). Printrakoon et al. 2008: table 1. Kesavan et al. 2009: 382, with in-text fig. Hamli et al. 2013: tables 2, 3, fig. 2a. Dechruksa et al. 2014: fig. 2f. Reid 2014: 27–29, 31, figs 2b, 8, 9. BEDO 2017b: 26, with in-text fig. Tudu et al. 2018: table 1. Wells et al. 2021: 125–126.
Cerithidea (Cerithidea) obtusa . Brandt 1974: 192–193, pl. 14, fig. 52. Houbrick 1984: 15–16, fig. 5c. Sri-aroon et al. 2004: table 1, fig. 3-1. Sri-aroon et al. 2005: tables 2–6. Ramakrishna et al. 2007: 6, 47. Nabhitabhata 2009: 95.

####### Referred material.

CUF-NKNY-G14 (23 shells; Figs 3M, 5E).

####### Habitat.

On firm mud and on trunks and stilt roots in mangrove forests as well as in fully marine and estuarine areas (Reid 2014).

####### Distribution.

Bay of Bengal; Indo-West Pacific, from southern Vietnam to Java and East Borneo (Reid 2014). Records of fossils from the Late Pliocene in Central and West Java, Indonesia, and from the Holocene in Thailand (Robba et al. 2004; Dharma 2005).

####### Record in Thailand.

Chanthaburi River, Gulf of Thailand and Andaman Sea (Nabhitabhata 2009; Wells et al. 2021).

####### Taxonomic remarks and comparisons.

This species has a more thickened and flared aperture compared to two similar species, *Cerithideaanticipata* Iredale, 1929 and *C.reidi* Houbrick, 1986. These two species are also common in Sahul (New Guinea and Australia), whereas *C.obtusa* is present in Sunda (Reid 2014). See also comprehensive taxonomic remarks in Reid (2014).

***Pirenella* Gray, 1847**

###### ﻿ Pirenella incisa

Taxon classificationAnimaliaCycloneritidaPotamididae

(Hombron & Jacquinot, 1848)

72DEB650-7D61-5444-92F2-C856D72E27B4

Figs 3I , 5F

**Figure 6.**
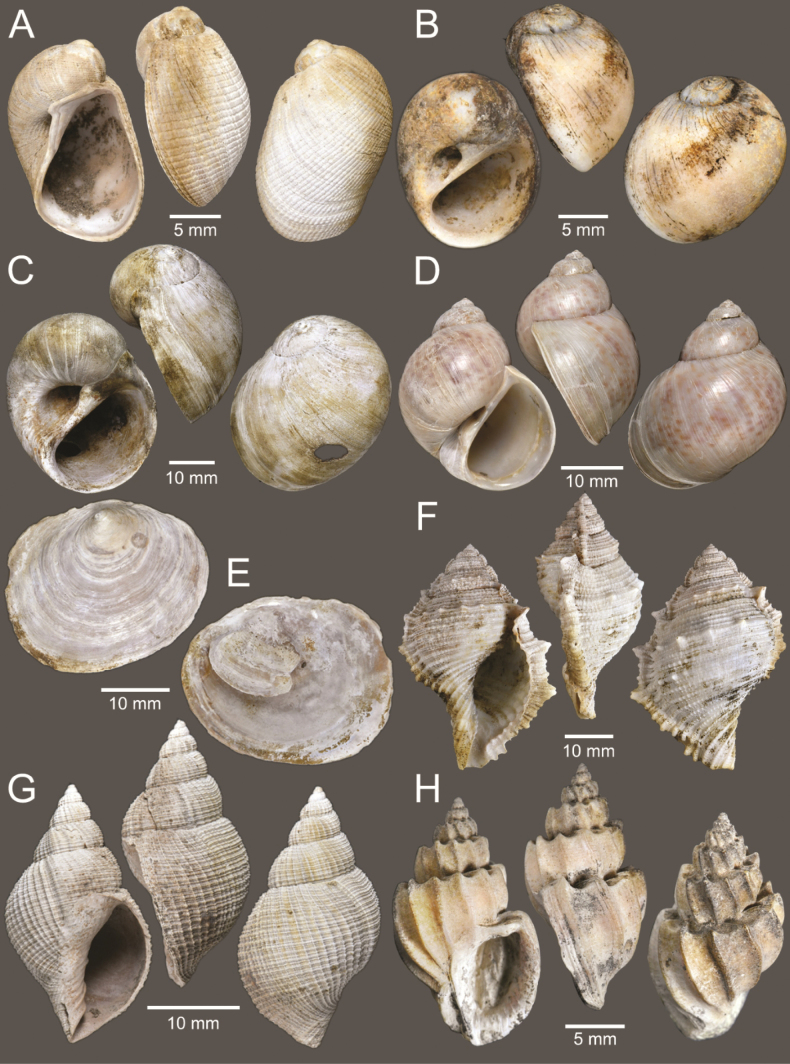
Gastropods **A***Eunaticinapapilla***B***Naticastellata***C***Naticavitellus***D***Paratectonaticatigrina***E***Ergaeawalshi***F***Bufonariarana***G***Mericaelegans***H***Scalptiascalariformis*.

**Figure 7.**
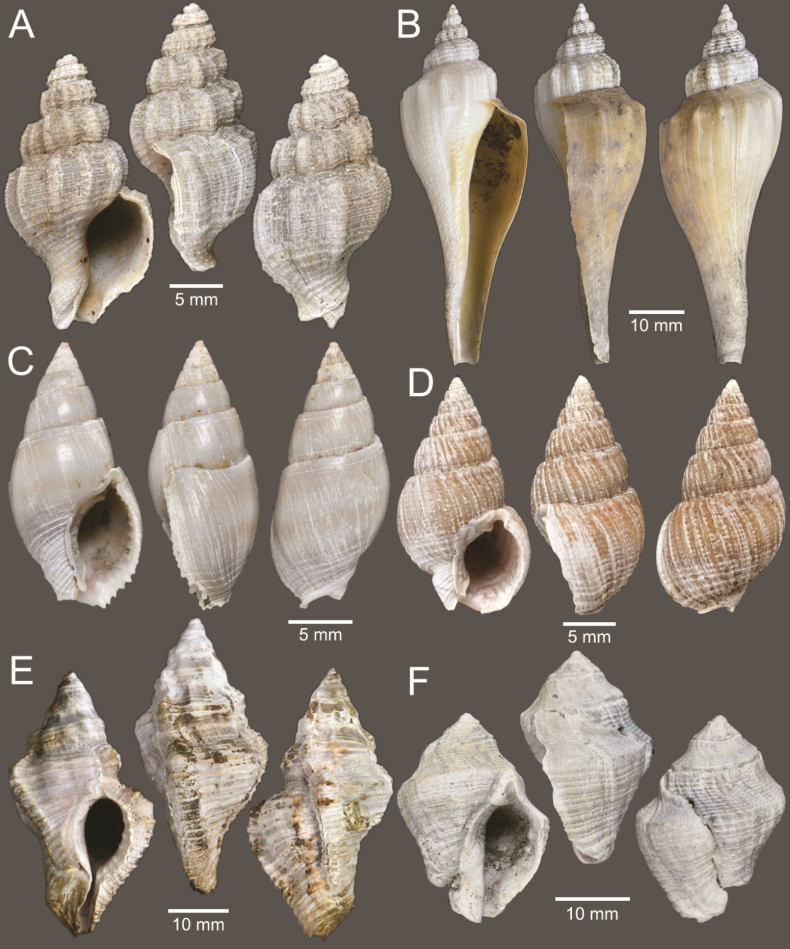
Gastropods **A***Pseudoneptuneavaricosa***B***Brunneifususternatanus***C***Nassariusmicans***D***Nassariussiquijorensis***E***Chicoreuscapucinus***F***Indothaisgradata*.

Cerithium incisum Hombron & Jacquinot, 1848: 97, pl. 23, figs 8, 9. Type locality: Borneo.
Cerithidea (Cerithideopsilla) djadjariensis [non Martin]. Sri-aroon et al. 2004: table 1, fig. 2-7.
Cerithideopsilla djadjariensis [non Martin]. Lozouet 2008: 284, pl. 87, fig. 9; pl. 88, fig. 2.
Pirenella incisa . Reid and Ozawa 2016: 30–32, figs 1, 2g, h, 4i, 10, 11a. Zvonareva and Kantor 2016: 415, fig. 5a–d. BEDO 2017b: 28, with in-text fig. Wells et al. 2021: 126.

####### Referred material.

CUF-NKNY-G10 (47 shells; Figs 3I, 5F).

####### Habitat.

On damp mud in mangrove forests. Also found on open surface areas of intertidal mudflats and on muddy shores of shrimp ponds (Reid and Ozawa 2016; Zvonareva and Kantor 2016).

####### Distribution.

East India; Indo-West Pacific, from southern China, the Philippines to West Sulawesi, and Flores Island (Reid and Ozawa 2016).

####### Record in Thailand.

Gulf of Thailand and Andaman Sea (Wells et al. 2021).

####### Taxonomic remarks and comparisons.

This species differs from the other two similar species, *Pirenellapupiformis* Ozawa & Reid, 2016 and *P.caiyingyai* (Qian, Fang & He, 2013), by the presence of an almost columnar appearance, a more oblique and more flared aperture with the projection of a lip next to a deep anterior canal, and three equal cords on each spire whorl with strong square nodules on axial ribs (Reid and Ozawa 2016). See comprehensive taxonomic remarks in Reid and Ozawa (2016).

***Telescopium* Montfort, 1810**

###### ﻿ Telescopium telescopium

Taxon classificationAnimaliaCycloneritidaPotamididae

(Linnaeus, 1758)

C44BE73B-E2B1-5465-9538-4EA9F3AD37F7

Figs 3K , 5G

**Figure 8.**
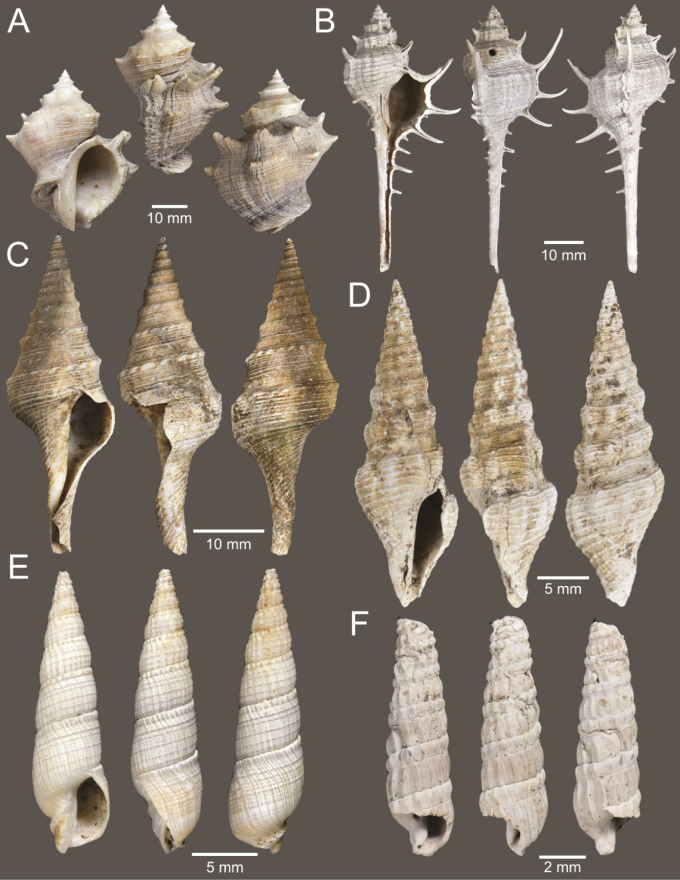
Gastropods **A***Indothaislacera***B***Murextrapa***C***Turriculajavana***D***Inquisitorvulpionis***E***Pristiterebramiranda***F***Granuliterebrabathyrhaphe*.

**Figure 9.**
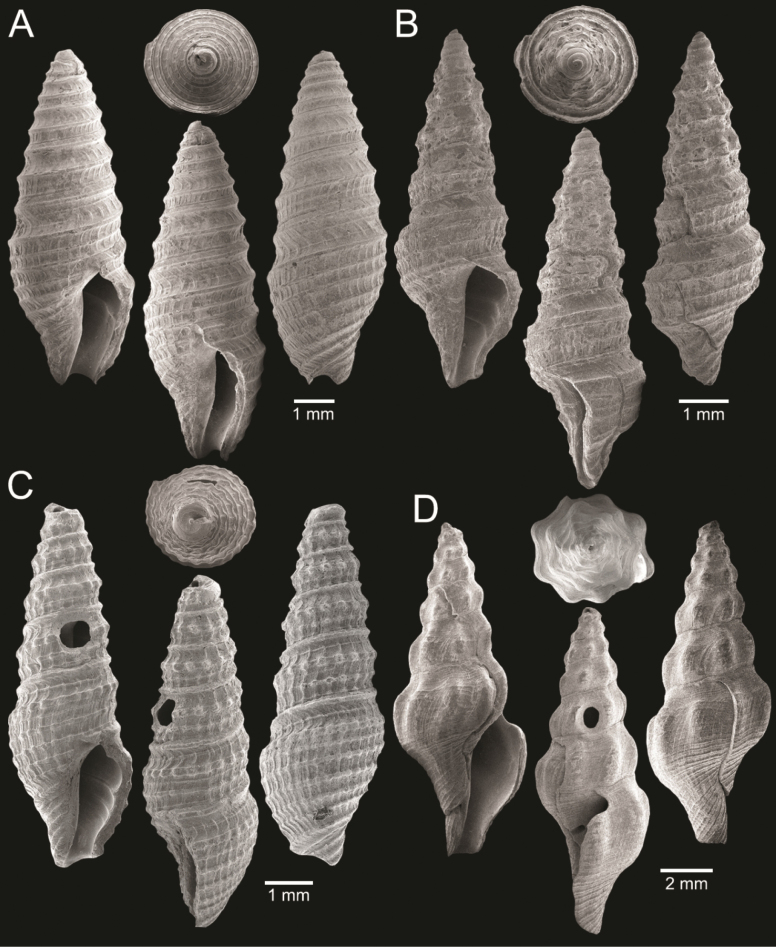
Gastropods **A***Maoritomellavallata***B***Pseudoetremafortilirata***C***Paradrilliamelvilli***D***Comitasilariae*.

**Figure 10.**
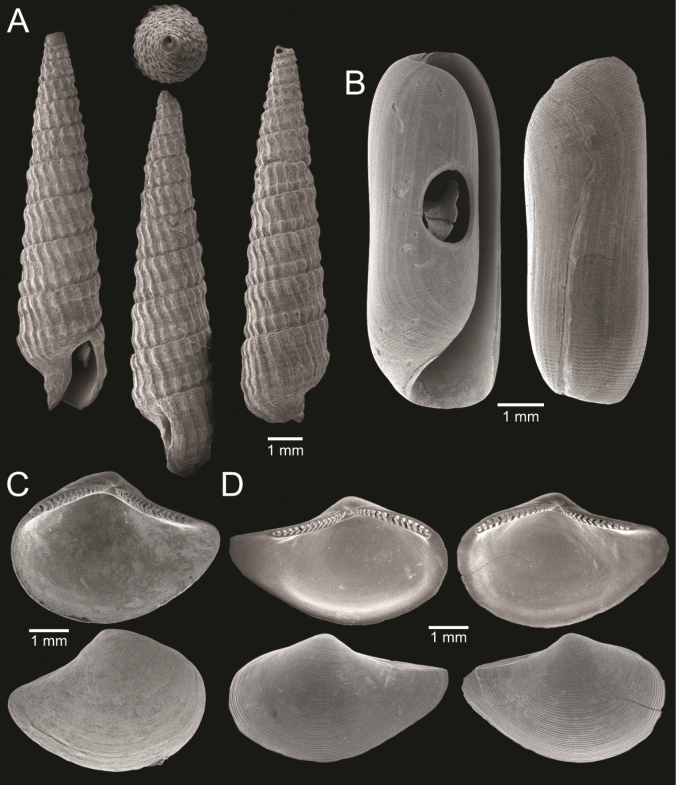
Gastropods and bivalves. **A***Duplicariatricincta***B***Cylichnamodesta***C***Jupiteriapuellata***D***Saccellamauritiana*.

**Figure 11.**
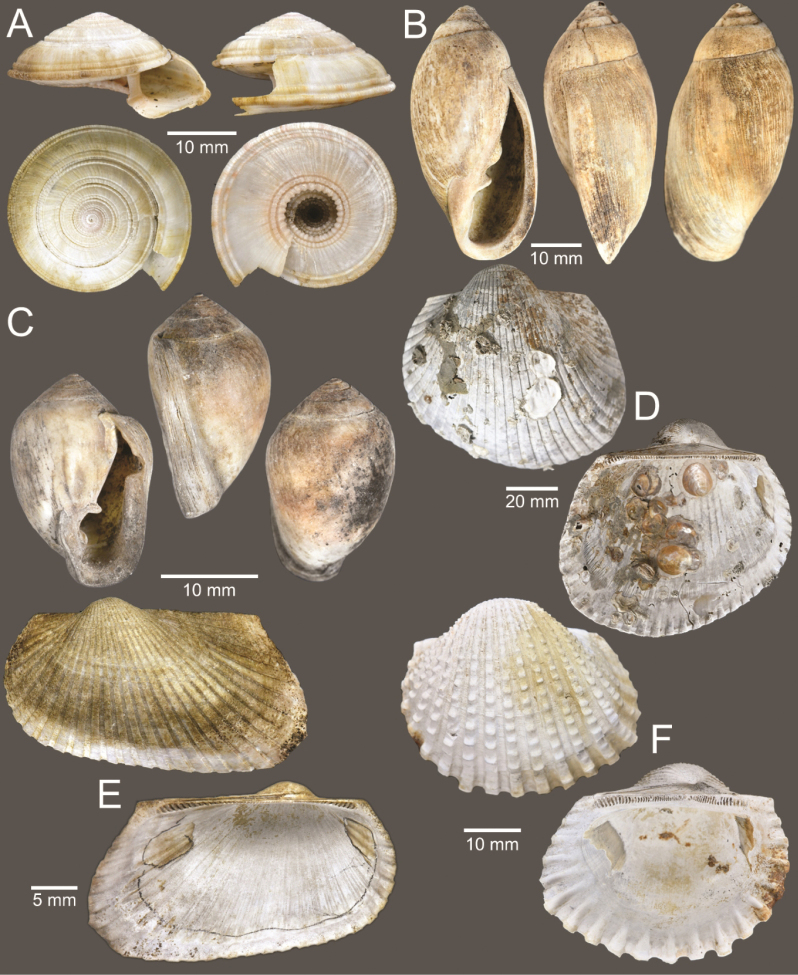
Gastropods and bivalves **A***Architectonicaperdix***B***Ellobiumaurisjudae***C***Cassidulanucleus***D***Anadarainaequivalvis***E***Anadaraindica***F***Tegillarcagranosa*.

**Figure 12.**
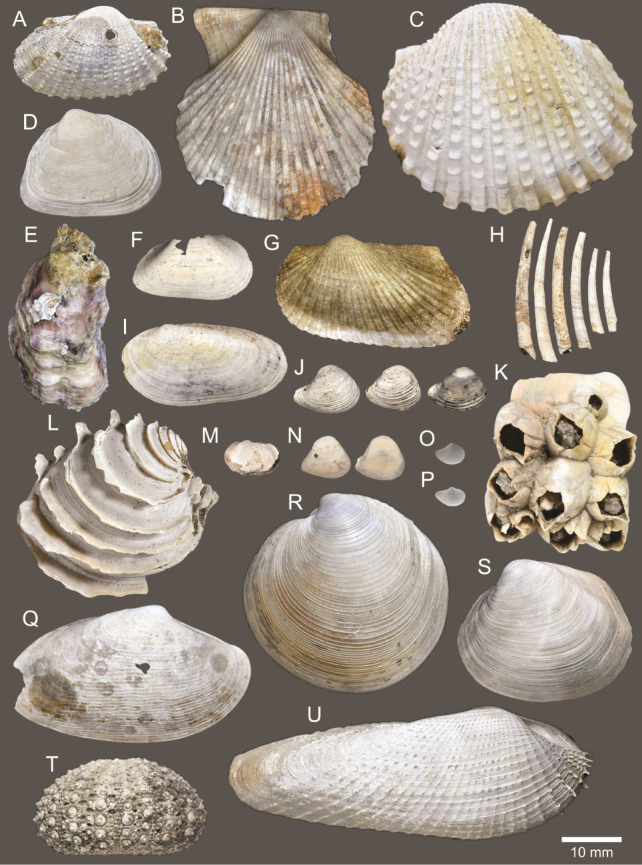
Size comparison of bivalves, scaphopod and other invertebrates found in this study **A***Tegillarcanodifera***B***Volachlamyssingaporina***C***Tegillarcagranosa***D***Noetiellapectunculiformis***E**Magallanacf.gigas**F***Estellacarolivacea***G***Anadaraindica***H***Dentaliumvariabile***I***Siliquaminima***J***Corbulafortisulcata***K***Fistulobalanuskondakovi***L***Placamenlamellatum***M***Martesiastriata***N***Potamocorbula* sp. **O***Jupiteriapuellata***P***Saccellamauritiana***Q***Paratapesundulatus***R***Dosiniadilecta***S***Joannisiellaoblonga***T***Temnotremasiamense***U***Pholasorientalis*.

**Figure 13.**
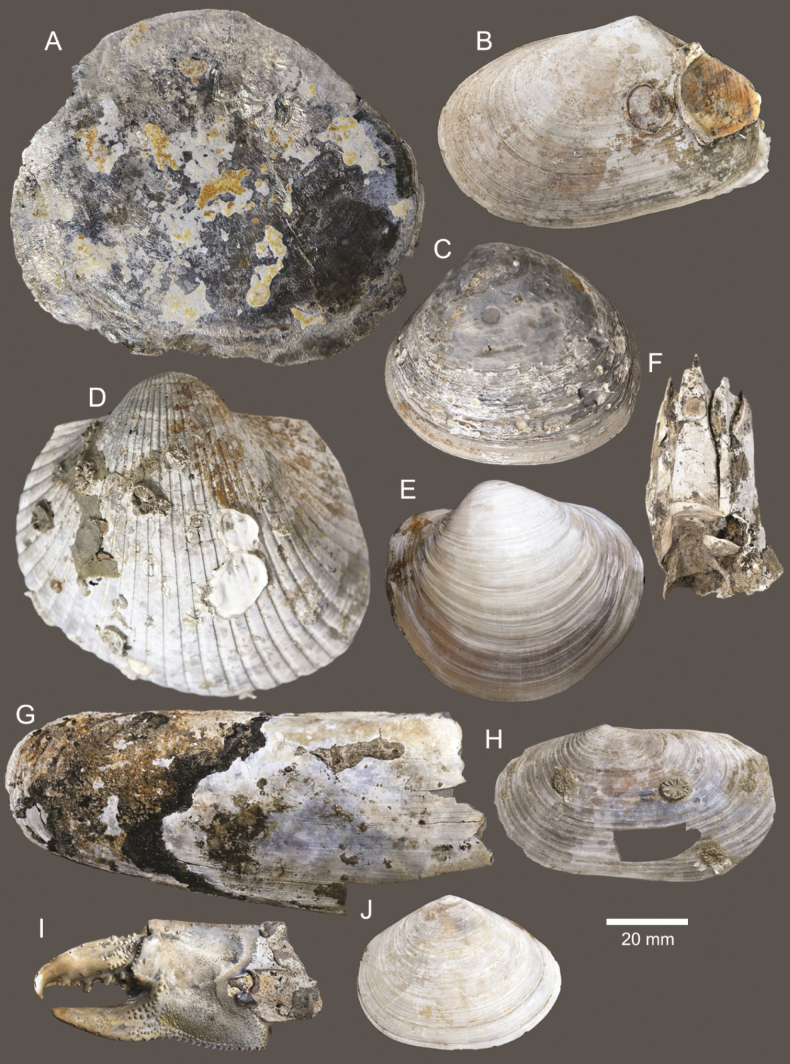
Size comparison of bivalves and other invertebrates found in this study **A***Placunaplacenta***B***Standellapellucida***C***Geloinabengalensis***D***Anadarainaequivalvis***E***Pegophysemabialata***F**Megabalanuscf.tintinnabulum**G***Cultellusmaximus***H***Lutrariacomplanata***I***Thalassina* sp. **J***Tellinidesconspicuus*.

**Figure 14.**
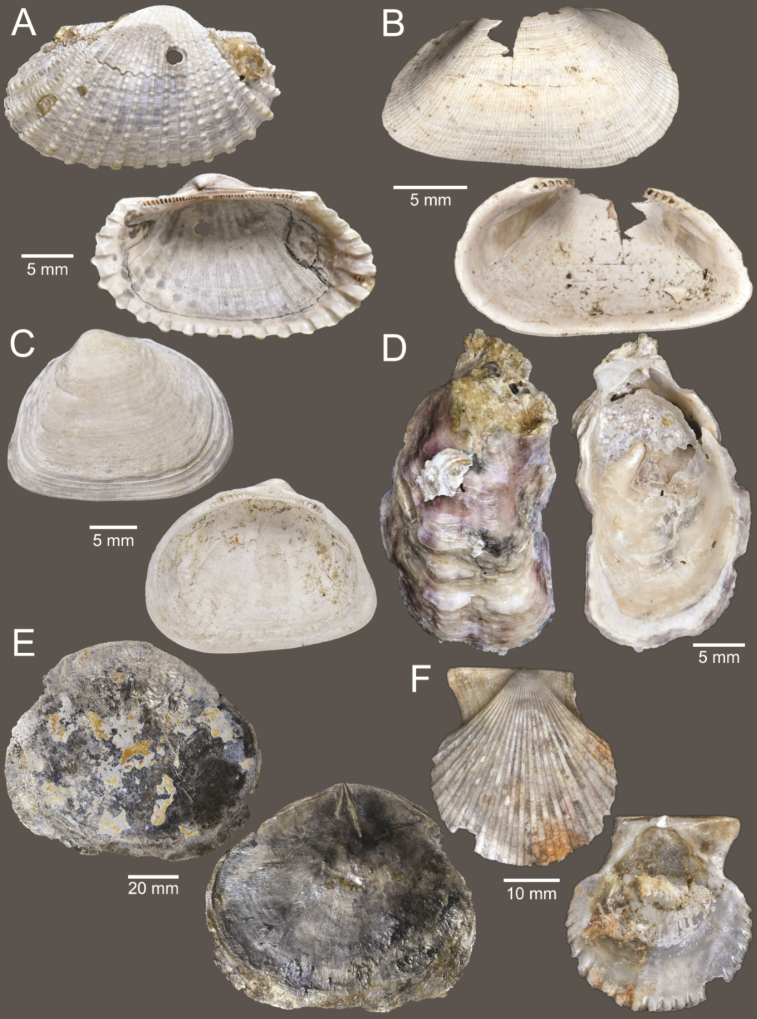
Bivalves **A***Tegillarcanodifera***B***Estellacarolivacea***C***Noetiellapectunculiformis***D**Magallanacf.gigas**E***Placunaplacenta***F***Volachlamyssingaporina*.

**Figure 15.**
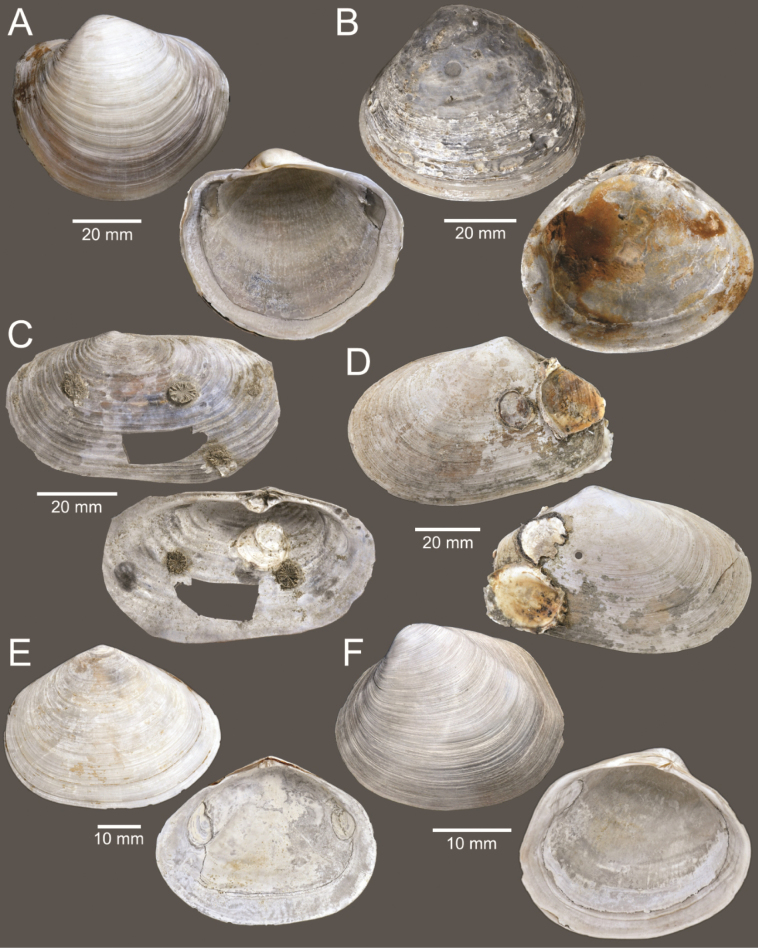
Bivalves **A***Pegophysemabialata***B***Geloinabengalensis***C***Lutrariacomplanata***D***Standellapellucida***E***Tellinidesconspicuus***F***Joannisiellaoblonga*.

**Figure 16.**
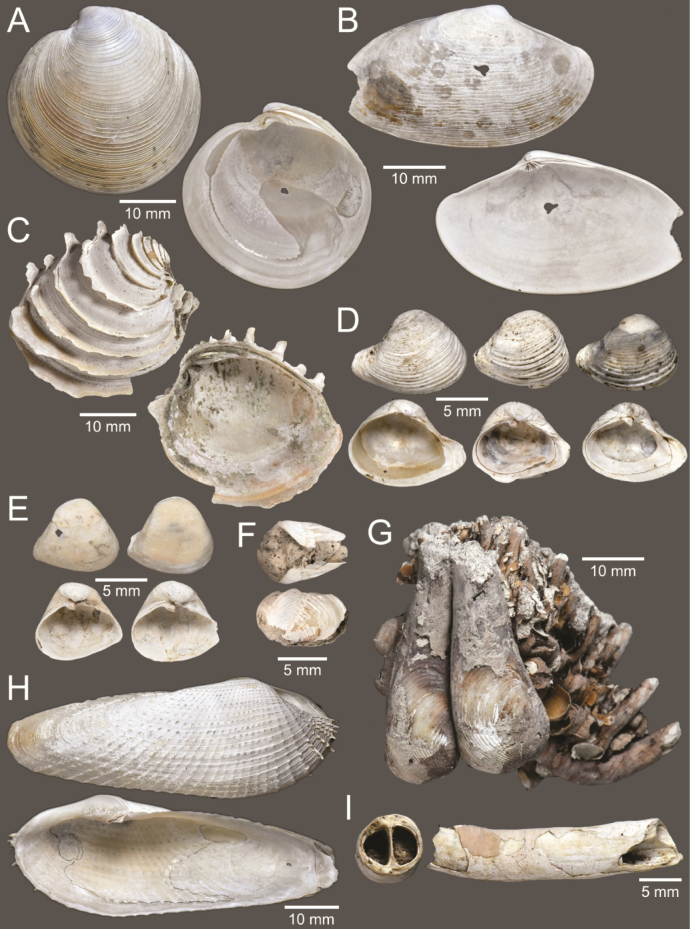
Bivalves **A***Dosiniadilecta***B***Paratapesundulatus***C***Placamenlamellatum***D***Corbulafortisulcata***E***Potamocorbula* sp. **F, G***Martesiastriata***G** showing the shells with burrow casts along with mangrove roots **H***Pholasorientalis***I** calcareous burrows of Teredinidae indet.

**Figure 17.**
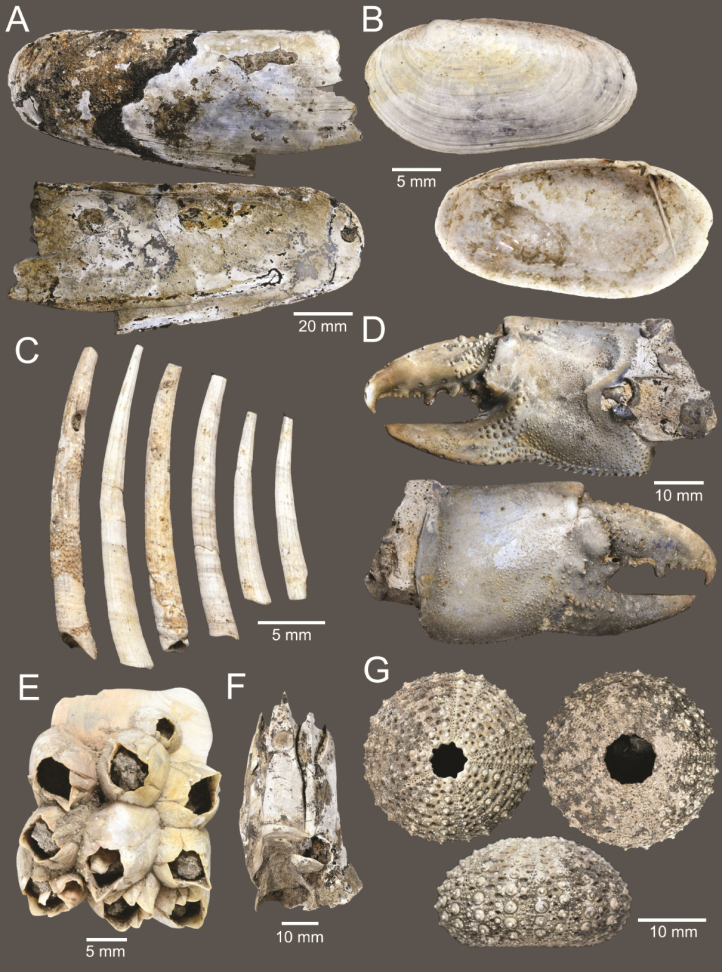
Bivalves and other invertebrates **A***Cultellusmaximus***B***Siliquaminima***C***Dentaliumvariabile***D***Thalassina* sp. **E***Fistulobalanuskondakovi***F**Megabalanuscf.tintinnabulum**G***Temnotremasiamense*.

**Figure 18.**
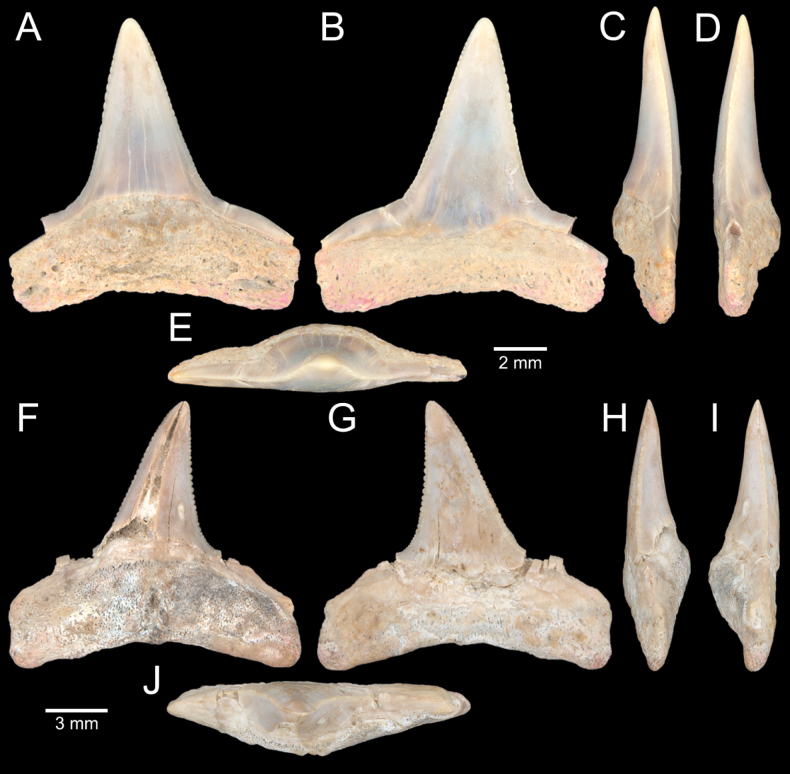
Carcharhinid shark teeth **A–E**Carcharhinuscf.amblyrhynchoides, specimen CUF-NKNY-S3-2 in **A** lingual **B** labial **C** mesial **D** distal and **E** apical views **F–J**Carcharhinuscf.amblyrhynchos, specimen CUF-NKNY-S3-10 in **F** lingual **G** labial **H** mesial **I** distal and **J** apical views.

Trochus telescopium Linnaeus, 1758: 760. Type locality: unknown.
Telescopium telescopium . Tesch 1920: 58–59, pl. 132, fig. 191. Cernohorsky 1972: 61, pl. 13, fig. 6. Brandt 1974: 196, pl. 15, fig. 61. Tantanasiriwong 1978: 7, fig. 69. Nateewathana et al. 1981: 57. Way and Purchon 1981: 315. Houbrick 1991: 291–304, figs 1–6. Wilson 1993: 133, pl. 15, fig. 21. Bosch et al. 1995: 56–57, fig. 186. Nateewathana 1995: 97–98, with in-text fig. Poutiers 1998b: 451, with in-text figs. Subba Rao and Dey 2000: 53. Swennen et al. 2001: 52, 111–112, with in-text fig., fig. 295. Hylleberg and Kilburn 2003: 38. Sri-aroon et al. 2004: table 1, fig. 3-3. Dharma 2005: 92, pl. 21, fig. 1; 308, pl. 119, fig. 4. Thach 2005: 45, pl. 9, figs 4, 7. Dey 2006: 31–32, figs 29–32. Ramakrishna et al. 2007: 48–49. Lozouet 2008: 284, pl. 87, figs 1, 3. Printrakoon et al. 2008: table 1. Kesavan et al. 2009: 382, with in-text fig. Nabhitabhata 2009: 97. Hamli et al. 2013: tables 2, 3, fig. 2e. Sanpanich and Duangdee 2013: 54. Dechruksa et al. 2014: fig. 2h. BEDO 2017b: 29, with in-text fig. Kantharajan et al. 2017: table 1, fig. 3-18. Okutani 2017: 797, pl. 63, fig. 11. Yang et al. 2017: 28, fig. 109. Tudu et al. 2018: table 1. Yadav et al. 2019: table 2, fig. 1o. Palanisamy et al. 2020: 2, fig. 1a–d. George et al. 2021: 1532, fig. 2a, b. Wells et al. 2021: 126.

####### Referred material.

CUF-NKNY-G64 (2 shells; Figs 3K, 5G).

####### Habitat.

On muddy floors of mangrove forests and intertidal mud flats (Poutiers 1998b; Okutani 2017).

####### Distribution.

Indian Ocean; Indo-West Pacific, from Japan to India (Poutiers 1998b; Okutani 2017; Palanisamy et al. 2020). Records of fossils from the Miocene to Quaternary in Indonesia (Tesch 1920; Dharma 2005).

####### Record in Thailand.

Gulf of Thailand and Andaman Sea (Wells et al. 2021).

####### Taxonomic remarks and comparisons.

This species is recognised by its high conical spire with a broad, rather flat base, and a twisted columella with a strong and central spiral ridge (Houbrick 1991; Poutiers 1998b).

**Subcohort Hypsogastropoda Ponder & Lindberg, 1997**

**Superfamily Naticoidea Guilding, 1834**

##### ﻿Family Naticidae Guilding, 1834

***Eunaticina* Fischer, 1885**

###### ﻿ Eunaticina papilla

Taxon classificationAnimaliaCycloneritidaNaticidae

(Gmelin, 1791)

C5C2CC60-BA40-5D03-A009-E5FC9FDF62BD

Figs 3G , 6A

Nerita papilla Gmelin, 1791: 3675. Type locality: Tranquebar [Tharangambadi, Tamil Nadu, India].
Eunaticina papilla . Cernohorsky 1971: 201–202, fig. 69. Cernohorsky 1972: 102, pl. 27, fig. 5. Tantanasiriwong 1978: 11, fig. 151. Nateewathana et al. 1981: 58. Way and Purchon 1981: 317. Majima 1989: 68–69, pl. 10, fig. 16, text-figs 15.36, 23.1a–d. Wilson 1993: 223, pl. 36, fig. 7. Bosch et al. 1995: 87, fig. 326. Swennen et al. 2001: 54, 122, fig. 378. Gemert 2003: 105. Hylleberg and Kilburn 2003: 56. Beu et al. 2004: 206, 208–211, fig. 22d–f, h. Robba et al. 2004: 75–76, pl. 9, fig. 7a, b. Dharma 2005: 176, pl. 63, fig. 25a–d; 342, pl. 136, fig. 12a, b. Ramakrishna et al. 2007: 9, 69–70. Robba et al. 2007: 91 (appendix). Hollmann 2008: 500, pl. 195, figs 1, 2. Nabhitabhata 2009: 123. Beu 2010: 152, fig. 21a. Torigoe and Inaba 2011: 55–56, pl. 2, fig. 17. Gopalakrishnan et al. 2012: 73. Öztürk and Bitlis 2013: 7, with in-text fig. Sanpanich and Duangdee 2013: 56. BEDO 2017b: 159, with in-text fig. Okutani 2017: 862, pl. 148, fig. 6. Yang et al. 2017: 52, fig. 215. Tudu et al. 2018: table 1. Albano et al. 2021: 12, fig. 5. Wells et al. 2021: 82.
Eunaticina papilla papilla . Thach 2005: 90, pl. 26, figs 3, 5.

####### Referred material.

CUF-NKNY-G19 (5 shells; Figs 3G, 6A).

####### Habitat.

Fine sandy bottoms in intertidal zones down to 30 m depth (Robba et al. 2004; Okutani 2017).

####### Distribution.

Red Sea to Indian Ocean; Indo-West Pacific, from Japan to Australia and Fiji Islands (Robba et al. 2004; Okutani 2017), as well as the Israeli Mediterranean shelf (Albano et al. 2021). Several records of fossils during the Late Miocene to Holocene in Indonesia, Japan, Taiwan, and Thailand (Beu et al. 2004; Robba et al. 2004; Dharma 2005).

####### Record in Thailand.

Gulf of Thailand and Andaman Sea (Wells et al. 2021).

####### Taxonomic remarks and comparisons.

This species is recognised by a regularly oval shell with a small spire and a large body whorl with low and wide spiral cords, which are broader than their interspaces and crossed by prominent axial ridges (Öztürk and Bitlis 2013). See also comprehensive taxonomic remarks in Beu et al. (2004).

***Natica* Scopoli, 1777**

###### ﻿ Natica stellata

Taxon classificationAnimaliaCycloneritidaNaticidae

Hedley, 1913

B9AEF19A-7946-5C43-8384-682C698BB82B

Figs 3D , 6B

Natica stellata Hedley, 1913: 299–300. Type locality: unknown. Cernohorsky 1972: 94–95, pl. 24, fig. 6. Wilson 1993: 217, pl. 36, fig. 24. Poutiers 1998b: 513, with in-text figs. Robba et al. 2004: 71, pl. 9, fig. 2a, b. Dharma 2005: 176, pl. 63, fig. 18. Robba et al. 2007: 91 (appendix). Hollmann 2008: 492, pl. 191, figs 9, 10a, b. Nabhitabhata 2009: 125. Torigoe and Inaba 2011: 76–77. Mukhopadhyay et al. 2013: 152–153, fig. 6. Sanpanich and Duangdee 2013: 57. Okutani 2017: 862, pl. 149, fig. 7. Wells et al. 2021: 83.
Natica (Natica) stellata . Cernohorsky 1971: 176–177, figs 6, 8–13.

####### Referred material.

CUF-NKNY-G05 (126 shells; Figs 3D, 6B).

####### Habitat.

Sandy gravel bottoms in sublittoral zones down to 20 m depth (Hollmann 2008; Okutani 2017).

####### Distribution.

Indo-West Pacific, from Japan to Australia (Cernohorsky 1972; Wilson 1993; Okutani 2017). Probably present in the Indian Ocean (Poutiers 1998b). Records of fossils from the Holocene in central Thailand (Robba et al. 2003, 2004).

####### Record in Thailand.

Gulf of Thailand and Andaman Sea (Wells et al. 2021).

####### Taxonomic remarks and comparisons.

This species differs from its similar species, *Naticavitellus*, in having a parietal callus forming a tongue-shaped extension over the posterior part of the umbilicus (Cernohorsky 1971). See also comprehensive taxonomic remarks in Cernohorsky (1971).

###### ﻿ Natica vitellus

Taxon classificationAnimaliaCycloneritidaNaticidae

(Linnaeus, 1758)

74D84E28-DA08-5DF3-9ADD-D924D9A1A024

Figs 3E , 6C

Nerita vitellus Linnaeus, 1758: 776. Type locality: Asiatic Ocean.
Natica vitellus . Tesch 1920: 70–71, pl. 132, fig. 207a, b. Cernohorsky 1972: 94, pl. 24, fig. 5. Tantanasiriwong 1978: 11, fig. 141. Nateewathana et al. 1981: 58. Way and Purchon 1981: 317. Majima 1989: 74–76, pl. 10, figs 1–12, text-figs 4.1, 15.38. Wilson 1993: 217, pl. 36, fig. 28. Bosch et al. 1995: 87, fig. 322. Poutiers 1998b: 515, with in-text figs. Subba Rao and Dey 2000: 78. Swennen et al. 2001: 54, 121, fig. 371. Hylleberg and Kilburn 2003: 57. Robba et al. 2003: table 5. Robba et al. 2004: 71–73, pl. 9, fig. 3a, b. Dharma 2005: 176, pl. 63, fig. 17; 342, pl. 136, fig. 5a, b. Ramakrishna et al. 2007: 8, 67. Robba et al. 2007: 91 (appendix). Hollmann 2008: 492, pl. 191, fig. 7. Nabhitabhata 2009: 125. Torigoe and Inaba 2011: 69–70. Sanpanich and Duangdee 2013: 57. BEDO 2017b: 162, with in-text fig. Okutani 2017: 862, pl. 149, fig. 4. Yang et al. 2017: 56, fig. 230. Harzhauser et al. 2018: 7– 8, pl. 1, figs 7–15. Tudu et al. 2018: table 1. Wells et al. 2021: 83.
Natica (Natica) vitellus . Cernohorsky 1971: 173–174, 176, figs 2–5. Ladd 1982: 39, pl. 6, fig. 7.
Natica vitellus vitellus . Thach 2005: 86, pl. 26, fig. 8.

####### Referred material.

CUF-NKNY-G04 (5 shells; Figs 3E, 6C).

####### Habitat.

Fine sandy or muddy bottoms in intertidal zones down to ~ 120 m depth (Robba et al. 2004); in clean coral-sand pockets and weedy-sand lagoons (Cernohorsky 1971).

####### Distribution.

Persian Gulf to Indian Ocean; Indo-West Pacific, from Japan to Australia (Bosch et al. 1995; Poutiers 1998b; Okutani 2017). Several records of fossils from the Early Miocene to Holocene in India, Indonesia, Japan, Malaysia, Myanmar, the Philippines, Polynesia, Taiwan, Vanuatu, and Thailand (Ladd 1982; Robba et al. 2004; Dharma 2005).

####### Record in Thailand.

Gulf of Thailand and Andaman Sea (Wells et al. 2021).

####### Taxonomic remarks and comparisons.

This species differs from its similar species, *Naticastellata*, in lacking a tongue-shaped extension of a parietal callus over the posterior part of the umbilicus (Cernohorsky 1971). See also comprehensive taxonomic remarks in Cernohorsky (1971).

***Paratectonatica* Azuma, 1961**

###### ﻿ Paratectonatica tigrina

Taxon classificationAnimaliaCycloneritidaNaticidae

(Röding, 1798)

FFE9A380-2885-5E72-A55D-CE5A10C74AD1

Figs 3Q , 6D

Cochlis tigrina Röding, 1798: 147. Type locality: unknown.
Natica tigrina . Tantanasiriwong 1978: 11, fig. 142. Nateewathana et al. 1981: 58. Dheeradilok et al. 1984: pl. 3, figs 9, 10. Wilson 1993: 217, pl. 36, fig. 10. Nateewathana 1995: 98, with in-text fig. Poutiers 1998b: 514, with in-text figs. Swennen et al. 2001: 54, 121, fig. 369. Hylleberg and Kilburn 2003: 57. Dharma 2005: 176, pl. 63, fig. 14. Ramakrishna et al. 2007: 8, 66–67, pl. 4, figs 45, 46. Nabhitabhata 2009: 125. Okutani 2017: 864, pl. 150, fig. 10. Yang et al. 2017: 56, fig. 229. Surakiatchai et al. 2018: table 5, pl. 1, fig. 6a, b.
Paratectonatica tigrina . Majima 1989: pl. 14, fig. 18. Robba et al. 2003: tables 2–5. Robba et al. 2004: 73, pl. 9, fig. 4a, b. Robba et al. 2007: 91 (appendix). Torigoe and Inaba 2011: 101–102, pl. 3, fig. 30. Kang et al. 2018: 357–358, 360, fig. 4.
Tectonatica tigrina . Thach 2005: 87, pl. 26, fig. 24.
Notocochlis tigrina . Gopalakrishnan et al. 2012: 73. Sanpanich and Duangdee 2013: 57. BEDO 2017b: 165, with in-text fig. Tudu et al. 2018: table 1. Yadav et al. 2019: table 2, fig. 1a. Wells et al. 2021: 84.

####### Referred material.

CUF-NKNY-G03 (70 shells; Figs 3Q, 6D).

####### Habitat.

Sandy mud in intertidal zones down to 30 m depth (Thach 2005; Okutani 2017).

####### Distribution.

India (Yadav et al. 2019); Indo-West Pacific, from Japan to Australia (Wilson 1993; Poutiers 1998b; Okutani 2017). Records of fossils from the Late Miocene to Quaternary in Indonesia and from the Quaternary in Thailand (Robba et al. 2003, 2004; Surakiatchai et al. 2018).

####### Record in Thailand.

Gulf of Thailand and Andaman Sea (Wells et al. 2021).

####### Taxonomic remarks and comparisons.

This species is recognised by its tall-spired shell with deeply impressed sutures and spiral rows of reddish-brown spots on a white background (Poutiers 1998b; Robba et al. 2004).

**Superorder Latrogastropoda Riedel, 2000**

**Superfamily Calyptraeoidea Lamarck, 1809**

##### ﻿Family Calyptraeidae Lamarck, 1809

***Ergaea* H. Adams & A. Adams, 1854**

###### ﻿ Ergaea walshi

Taxon classificationAnimaliaCycloneritidaCalyptraeidae

(Reeve, 1859)

82DFEB72-5505-56A1-AA48-78BFABD9A08D

Figs 3H , 6E

Crepidula walshi Reeve, 1859: Crepidula, pl. 3, sp. 17. Type locality: Singapore; Ceylon [Sri Lanka]. Tantanasiriwong 1978: 9. Bosch et al. 1995: 69, fig. 230. Subba Rao and Dey 2000: 64. Swennen et al. 2001: 54, 117, text-fig. 344, fig. 344. Gemert 2003: 104. Robba et al. 2003: tables 3–5. Robba et al. 2004: 66–67, pl. 8, fig. 8a, b. Ramakrishna et al. 2007: 7, 55–56. Robba et al. 2007: 91 (appendix).
Ergaea walshi . Nateewathana et al. 1981: 57. Nabhitabhata 2009: 109. Low and Tan 2014: 11–13, fig. 1. BEDO 2017b: 117, with in-text fig. Tudu et al. 2018: table 1. Wells et al. 2021: 75.
Crepidula walshii [sic]. Way and Purchon 1981: 316.
Siphopatella walshi . Hylleberg and Kilburn 2003: 47. Sanpanich and Duangdee 2013: 55. Okutani 2017: 838, pl. 114, fig. 3. Yang et al. 2017: 32, fig. 117.
Crepidula (Siphopatella) walshi . Dharma 2005: 78, pl. 14, fig. 13a, b; 342, pl. 136, fig. 15a, b.
Crepidula (Ergaea) walshi . Thach 2005: 66, pl. 10, fig. 27.

####### Referred material.

CUF-NKNY-G25 (10 shells; Figs 3H, 6E).

####### Habitat.

Attached to the aperture of other shelled marine organisms, in intertidal zones down to 40 m depth (Robba et al. 2004; Thach 2005; Low and Tan 2014).

####### Distribution.

Persian Gulf; Indo-West Pacific, from Japan to the Arafura Sea (Robba et al. 2004; Okutani 2017). Several records of fossils from the Late Miocene to Holocene in India, Indonesia, Japan, Taiwan, and Thailand (Robba et al. 2004; Dharma 2005).

####### Record in Thailand.

Gulf of Thailand and Andaman Sea (Wells et al. 2021).

####### Taxonomic remarks and comparisons.

This species is recognised by an irregularly subrectangular and flattened shell, with a small apex close to the posterior margin, and a shelf-like internal septum attached just inside the posterior margin (Robba et al. 2004). See also comprehensive taxonomic remarks in Low and Tan (2014).

**Superfamily Tonnoidea Suter, 1913**

##### ﻿Family Bursidae Thiele, 1925

***Bufonaria* Schumacher, 1817**

###### ﻿ Bufonaria rana

Taxon classificationAnimaliaCycloneritidaBursidae

(Linnaeus, 1758)

FFAEB65F-C743-5237-9AE5-D2D16681DD25

Figs 3N , 6F

Murex rana Linnaeus, 1758: 748. Type locality: Asiatic Ocean.
Bursa (Bufonaria) rana . Cernohorsky 1972: 119, pl. 32, fig. 8.
Bursa rana . Tantanasiriwong 1978: 12, fig. 170. Nateewathana et al. 1981: 59. Way and Purchon 1981: 317. Swennen et al. 2001: 55, 122, fig. 380.
Bufonaria rana . Wilson 1993: 226, pl. 43, fig. 1a, b. Aungtonya and Hylleberg 1998: 319. Poutiers 1998b: 551, with in-text figs. Subba Rao and Dey 2000: 92. Hylleberg and Kilburn 2003: 59. Robba et al. 2004: 76–77, pl. 9, fig. 9a, b. Dharma 2005: 194, pl. 72, fig. 1a–e; 352, pl. 141, fig. 7a–c. Ramakrishna et al. 2007: 10, 81, pl. 6, figs 69, 70. Robba et al. 2007: 91 (appendix). Beu 2008: 618, pl. 254, figs 6, 7a, b. Nabhitabhata 2009: 137. Sanpanich and Duangdee 2013: 58. BEDO 2017b: 112, with in-text fig. Okutani 2017: 867–868, pl. 154, fig. 5. Yang et al. 2017: 64, fig. 266. Harzhauser et al. 2018: 13–14, pl. 3, figs 6–9. Tudu et al. 2018: table 1. Yadav et al. 2019: table 2, fig. 1b. Wells et al. 2021: 89.
Bufonaria (Bufonaria) rana . Cossignani 1994: 33–35, with in-text figs. Bosch et al. 1995: 102, fig. 372. Gemert 2003: 105. Thach 2005: 96, pl. 28, fig. 8.

####### Referred material.

CUF-NKNY-G06, G07 (18 shells; Figs 3N, 6F).

####### Habitat.

Muddy or sandy bottoms at a depth from 20 to 100 m (Okutani 2017; Yang et al. 2017).

####### Distribution.

Red Sea to Indian Ocean; Indo-West Pacific, from Japan to Australia (Robba et al. 2004; Okutani 2017; Yadav et al. 2019). Several records of fossils from the Miocene to Quaternary in Indonesia, Japan, the Philippines, and Taiwan (Robba et al. 2004; Dharma 2005; Harzhauser et al. 2018).

####### Record in Thailand.

Gulf of Thailand and Andaman Sea (Wells et al. 2021).

####### Taxonomic remarks and comparisons.

This species is recognised based on the descriptions and figures in Robba et al. (2004) and Harzhauser et al. (2018), specifically in having two prominent varices placed at either periphery, running vertically uninterrupted, or slightly staggered, from apex to base, and bearing three short spines.

**Order Neogastropoda Wenz, 1938**

**Superfamily Volutoidea Rafinesque, 1815**

##### ﻿Family Cancellariidae Forbes & Hanley, 1851

***Merica* H. Adams & A. Adams, 1854**

###### ﻿ Merica elegans

Taxon classificationAnimaliaNeogastropodaCancellariidae

(Sowerby I, 1822)

C46A9300-6085-5B46-BB04-D5C52350F960

Figs 3L , 6G

Cancellaria elegans Sowerby I, 1822: Cancellaria, pl. 218, fig. 3. Type locality: unknown. Tantanasiriwong 1978: 17. Nateewathana et al. 1981: 61. Robba et al. 2007: 51–52, fig. 19d, e; 93 (appendix).
Cancellaria asperella [sic]. Cernohorsky 1972: 179, pl. 50, fig. 3 right.
Merica elegans . Verhecken 1986: 40–41, fig. 9. Verhecken 1997: 307–308, fig. 36. Swennen et al. 2001: 57, 133, fig. 461. Hylleberg and Kilburn 2003: 100. Dharma 2005: 134, pl. 42, fig. 1. Thach 2005: 188, pl. 58, figs 6, 10. Hemmen 2007: 128–129, with in-text fig. Verhecken 2008: 818, pl. 704, fig. 5a, b. BEDO 2017b: 221, with in-text fig. Tudu et al. 2018: table 1. Wells et al. 2021: 121.

####### Referred material.

CUF-NKNY-G18 (3 shells; Figs 3L, 6G).

####### Habitat.

Sandy and muddy bottoms at a depth from 10 to 30 m (Robba et al. 2004; Thach 2005).

####### Distribution.

Indo-West Pacific, from Japan to the Philippines. Records of fossils from the Miocene in Indonesia, and from the Quaternary in Japan (Robba et al. 2007).

####### Record in Thailand.

Gulf of Thailand and Andaman Sea (Wells et al. 2021).

####### Taxonomic remarks and comparisons.

This species differs from its similar species, *Mericaasperella* (Lamarck, 1822), in having a slenderer and more fusiform shell with a narrower aperture and a sculptured shell with finer and more numerous ribs crossed by spiral ridges nearly of the same strength, showing a rectangular reticulated pattern (Robba et al. 2007).

***Scalptia* Jousseaume, 1887**

###### ﻿ Scalptia scalariformis

Taxon classificationAnimaliaNeogastropodaCancellariidae

(Lamarck, 1822)

950BC205-86C6-52AE-AD86-F80C2281C7ED

Figs 3J , 6H

Cancellaria scalariformis Lamarck, 1822: 115. Type locality: unknown.
Cancellaria (Trigonostoma) scalariformis . Tesch 1915: 39, pl. 79, fig. 81a, b.
Trigonostoma scalariformis . Cernohorsky 1972: 180, pl. 50, fig. 2, 2a. Garrard 1975: 27–29, figs 4 (3, 4). Way and Purchon 1981: 320. Wilson 1994: 178, pl. 37, fig. 7a, b.
Scalptia scalariformis . Verhecken 1986: 53–55, figs 28–35. Swennen et al. 2001: 57, 133, fig. 462. Hylleberg and Kilburn 2003: 101. Robba et al. 2004: 129–130, pl. 17, fig. 6. Dharma 2005: 134, pl. 42, fig. 12a, b. Hemmen 2007: 277–278, with in-text figs. Robba et al. 2007: 93 (appendix). Okutani 2017: 1054, pl. 344, fig. 4. Yang et al. 2017: 102, fig. 415. Tudu et al. 2018: table 1. Wells et al. 2021: 121. Chan and Lau 2022: 1–2, figs 1–3.
Scalptia (Scalptia) scalariformis . Thach 2005: 188, pl. 58, figs 25, 27, 29.

####### Referred material.

CUF-NKNY-G64 (1 shell; Figs 3J, 6H).

####### Habitat.

Sand and muddy bottoms at a depth from 20 to 40 m in sublittoral zones (Thach 2005; Okutani 2017; Yang et al. 2017).

####### Distribution.

Indian Ocean; Indo-West Pacific, from Japan to Australia (Robba et al. 2004; Okutani 2017). Several records of fossils from the Pliocene to Holocene in the Indo-Pacific area, including Indonesia and Thailand (Robba et al. 2004).

####### Record in Thailand.

Gulf of Thailand (Wells et al. 2021).

####### Taxonomic remarks and comparisons.

This species differs from its similar species, *Scalptiabicolor* (Hinds, 1843), by having a higher and narrower body whorl and a less widely open umbilicus (Robba et al. 2007).

**Superfamily Buccinoidea Rafinesque, 1815**

##### ﻿Family Buccinidae Rafinesque, 1815

***Pseudoneptunea* Kobelt, 1882**

###### ﻿ Pseudoneptunea varicosa

Taxon classificationAnimaliaNeogastropodaBuccinidae

(Röding, 1798)

6360DA51-1F28-58C1-BA03-2D107FC68E0F

Figs 4K , 7A

Neptunea varicosa Röding, 1798: 116. Type locality: unknown.
Siphonalia (Pseudoneptunea) aff. varicosa . Tesch 1915: 53, pl. 80, fig. 114a, b.
Pseudoneptunea varicosa . Cernohorsky 1975: 217–218, figs 10–12. Way and Purchon 1981: 318. Swennen et al. 2001: 56, 128, fig. 428. Robba et al. 2004: 106–107, pl. 14, fig. 5a, b. Dharma 2005: 100, pl. 25, fig. 7a, b; 312, pl. 121, fig. 9a, b. Thach 2005: 135, pl. 39, figs 12, 15. Robba et al. 2007: 93 (appendix). Nabhitabhata 2009: 148. Sanpanich and Duangdee 2013: 63. Wells et al. 2021: 95.

####### Referred material.

CUF-NKNY-G13, G16, G17 (25 shells; Figs 4K, 7A).

####### Habitat.

Shallow water at a depth from 10 to 15 m (Thach 2005).

####### Distribution.

Indo-West Pacific, from Vietnam to Indonesia (Robba et al. 2004; Dharma 2005; Thach 2005). Records of fossils from the Pliocene and Quaternary in Indonesia and from the Holocene in Thailand (Robba et al. 2004).

####### Record in Thailand.

Gulf of Thailand and Andaman Sea (Wells et al. 2021).

####### Taxonomic remarks and comparisons.

This species is recognised based on the descriptions and figures in Cernohorsky (1975) and Robba et al. (2004), specifically in having subangulate whorls and a sculpture of crisp main spirals with an occasional intermediate spiral thread.

##### ﻿Family Melongenidae Gill, 1871

***Brunneifusus* Dekkers, 2018**

###### ﻿ Brunneifusus ternatanus

Taxon classificationAnimaliaNeogastropodaMelongenidae

(Gmelin, 1791)

40733EDF-336E-5008-A673-1C188F768D92

Figs 4A , 7B

Murex ternatanus Gmelin, 1791: 3554. Type locality: Ternate Island, North Maluku, Indonesia.
Pugilina ternatana . Tantanasiriwong 1978: 15. Nateewathana et al. 1981: 60. Poutiers 1998b: 586, with in-text figs.
Hemifusus ternatanus . Yokoyama 1927: 393. Way and Purchon 1981: 318. Swennen et al. 2001: 56, 130, fig. 445. Gemert 2003: 106, with in-text fig. Hylleberg and Kilburn 2003: 88. Robba et al. 2004: 122–123, pl. 16, fig. 7a, b. Dharma 2005: 108, pl. 29, fig. 2a, b; 316, pl. 123, fig. 7a, b. Robba et al. 2007: 93 (appendix). Nabhitabhata 2009: 162. Sanpanich and Duangdee 2013: 61. BEDO 2017b: 288, with in-text fig. Tudu et al. 2018: table 1.
Brunneifusus ternatanus . Dekkers 2018: 41–42, pl. 2, figs 5, 6. Alf and Thach 2021: pl. 1, figs 1–9, pl. 2, figs 1–3. Wells et al. 2021: 97.

####### Referred material.

CUF-NKNY-G21, G22 (71 shells; Figs 4A, 7B).

####### Habitat.

Mud and muddy sand bottoms from the low tide mark to ~ 10–50 m depth (Poutiers 1998b; Swennen et al. 2001; Dekkers 2018).

####### Distribution.

Eastern Indian Ocean; Indo-West Pacific, from Taiwan to Indonesia and the Philippines (Poutiers 1998b; Robba et al. 2004). Records of fossils from the Pliocene and Quaternary in Indonesia, Japan, and Taiwan (Robba et al. 2004; Dharma 2005).

####### Record in Thailand.

Gulf of Thailand and Andaman Sea (Wells et al. 2021).

####### Taxonomic remarks and comparisons.

This species is the sole member of the genus *Brunneifusus*, characterised by a determinate outer lip, compared to the indeterminate growth of the outer lip in *Hemifusus* Swainson, 1840 (Alf and Thach 2021). See also comprehensive taxonomic remarks in Alf and Thach (2021).

##### ﻿Family Nassariidae Iredale, 1916

***Nassarius* Duméril, 1805**

###### ﻿ Nassarius micans

Taxon classificationAnimaliaNeogastropodaNassariidae

(A. Adams, 1852)

9B957AFA-F0BC-5718-A323-001E8232D76F

Figs 3O , 7C

Nassa micans A. Adams, 1852: 106. Type locality: Cagayan, Misamis, Mindanao.
Nassarius (Zeuxis) micans . Cernohorsky 1984: 147–148, pl. 29, figs 8–11. Robba et al. 2003: table 5. Robba et al. 2004: 120, pl. 16, fig. 4a, b. Dharma 2005: 106, pl. 28, fig. 16a, b. Robba et al. 2007: 93 (appendix). Okutani 2017: 914, pl. 202, fig. 11
Nassarius micans . Gopalakrishnan et al. 2012: 77. Wells et al. 2021: 99.

####### Referred material.

CUF-NKNY-G27 (53 shells; Figs 3O, 7C).

####### Habitat.

Sandy bottoms in subtidal zones down to 50 m depth (Robba et al. 2004; Okutani 2017).

####### Distribution.

Gulf of Oman to India; Indo-West Pacific, from Japan to Papua New Guinea (Cernohorsky 1984; Robba et al. 2004; Okutani 2017). Records of fossils from the Holocene in Thailand (Robba et al. 2004).

####### Record in Thailand.

Gulf of Thailand (Wells et al. 2021).

####### Taxonomic remarks and comparisons.

This species differs from its similar species, *Nassariuscomptus* (A. Adams, 1852), by having a longer body whorl with narrowly subcanaliculate sutures, a more prominent varix, and a narrower and more elongate aperture (Cernohorsky 1984). See also comprehensive taxonomic remarks in Cernohorsky (1984).

###### ﻿ Nassarius siquijorensis

Taxon classificationAnimaliaNeogastropodaNassariidae

(A. Adams, 1852)

ED5D1426-F5AE-54F5-9583-968FBA7A03F0

Figs 3P , 7D

Nassa siquijorensis A. Adams, 1852: 97. Type locality: Island of Siquijor, Philippines.
Nassa (Zeuxis) siquijorensis . Tesch 1915: 59, pl. 81, figs 128a, b, 129a, b.
Nassarius (Zeuxis) siquijorensis . Cernohorsky 1978: 88, pl. 27, fig. 8. Cernohorsky 1984: 134–136, pl. 25, figs 12–14, pl. 26, figs 1–5. Robba et al. 2003: table 5. Robba et al. 2004: 120–121, pl. 16, fig. 5a, b. Dharma 2005: 106, pl. 28, fig. 14a–c. Robba et al. 2007: 93 (appendix).
Nassarius siquijorensis . Nobuhara 1993: fig. 8-1a, 8-1b. Swennen et al. 2001: 56, 130, fig. 442. Hylleberg and Kilburn 2003: 87. Kool 2007: figs 17–20. Martin 2008: 120, pl. 355, fig. 13; 128, pl. 359, fig. 13. Surakiatchai et al. 2018: table 5, pl. 1, fig. 9a, b. Wells et al. 2021: 99.
Zeuxis siquijorensis . Thach 2005: 148, pl. 44, fig. 25.
Nassarius siquinjorensis [sic]. Yang et al. 2017: 90, fig. 371.

####### Referred material.

CUF-NKNY-G01 (10 shells; Figs 3P, 7D).

####### Habitat.

Sandy and muddy bottoms from subtidal zones to 450 m depth (Cernohorsky 1984; Thach 2005; Yang et al. 2017).

####### Distribution.

Red Sea to India; Indo-West Pacific, from Japan to Indonesia and New Caledonia (Cernohorsky 1984; Dharma 2005; Yang et al. 2017). Records of fossils from the Pliocene in Indonesia and from the Holocene in Thailand (Cernohorsky 1984; Surakiatchai et al. 2018).

####### Record in Thailand.

Gulf of Thailand and Andaman Sea (Wells et al. 2021).

####### Taxonomic remarks and comparisons.

This species differs from its similar species, *Nassariuscastus* (Gould, 1850), in having a more tapering spire, canaliculate sutures, and considerably more numerous axial ribs (Cernohorsky 1984). See also comprehensive taxonomic remarks in Cernohorsky (1984) and Kool (2007).

**Superfamily Muricoidea Rafinesque, 1815**

##### ﻿Family Muricidae Rafinesque, 1815

***Chicoreus* Montfort, 1810**

###### ﻿ Chicoreus capucinus

Taxon classificationAnimaliaNeogastropodaMuricidae

(Lamarck, 1822)

6C0CB311-FAE6-5519-ABF2-46CD21585408

Figs 4E , 7E

Murex capucinus Lamarck, 1822: 164. Type locality: unknown.
Naquetia capucina . Tantanasiriwong 1978: 13. Nateewathana et al. 1981: 59. Way and Purchon 1981: 318. Bussarawit 1991: 31.
Chicoreus (Rhizophorimurex) capucinus . Houart 1992: 106–109, fig. 217. Tan 2000: 504. Dharma 2005: 164, pl. 57, fig. 20; 336, pl. 133, fig. 9a–c. Thach 2005: 114, pl. 36, fig. 2. Merle et al. 2011: 99, text-fig. 39.
Chicoreus capucinus . Wilson 1994: 27, pl. 3, fig. 12a, b. Middelfart 1997: 358, pl. 2, fig. 7. Hylleberg and Kilburn 2003: 69. Sri-aroon et al. 2005: tables 2, 3, 5, 6. Houart 2008: 154, pl. 372, fig. 1. Printrakoon et al. 2008: table 1. Nabhitabhata 2009: 141. BEDO 2017b: 300, with in-text fig. Sanpanich and Duangdee 2013: 62. Dechruksa et al. 2014: fig. 4d. Wells et al. 2021: 113.
Chicoreus (Naquetia) capucinus . Subba Rao and Dey 2000: 100.
Chicoreus copucinus [sic]. Sri-aroon et al. 2004: table 1.

####### Referred material.

CUF-NKNY-G11, G12 (30 shells; Figs 4E, 7E).

####### Habitat.

Muddy sand and rocks in mangrove forests (Thach 2005).

####### Distribution.

Indo-West Pacific, from the Philippines to Australia to Fiji Islands (Houart 1992). Records of fossils from the Middle Miocene to Late Pliocene in Indonesia (Dharma 2005).

####### Record in Thailand.

Gulf of Thailand and Andaman Sea (Wells et al. 2021).

####### Taxonomic remarks and comparisons.

This species is recognised based on the descriptions and figures in Houart (1992) and Merle et al. (2011), specifically in having a rounded last whorl usually associated with three spineless varices, each with a short and webbed expansion on adapical section, an aperture with a characteristically small callus with straight, adherent columellar lip, and a broad siphonal canal. See also comprehensive taxonomic remarks in Houart (1992).

***Indothais* Claremont et al., 2013**

###### ﻿ Indothais gradata

Taxon classificationAnimaliaNeogastropodaMuricidae

(Jonas, 1846)

063B82E5-7814-5FE0-A1F5-8386BFC59B1E

Figs 4F , 7F

Purpura gradata Jonas, 1846: 14–15. Type locality: Indian Ocean, near Singapore.
Thais gradata . Bussarawit 1991: 33. Middelfart 1997: 367–368, pl. 4, fig. 1. Tan 2000: 499. Hylleberg and Kilburn 2003: 76. Dharma 2005: 170, pl. 60, fig. 11a, b; 340, pl. 135, fig. 1a–e. Printrakoon et al. 2008: table 1. Yang et al. 2017: 76, fig. 316.
Stramonita gradata . Wilson 1994: 47, pl. 4, fig. 5a, b. Thach 2005: 128, pl. 37, figs 33, 37.
Indothais gradata . Okutani 2017: 963, pl. 255, fig. 9. Wells et al. 2021: 114.

####### Referred material.

CUF-NKNY-G29 (1 shell; Figs 4F, 7F).

####### Habitat.

Rocky bottom at a depth from 1 to 5 m (Thach 2005; Okutani 2017).

####### Distribution.

Indo-West Pacific, from Japan to Australia (Okutani 2017; Yang et al. 2017). Records of fossils from the Middle to Late Pliocene in Indonesia (Dharma 2005).

####### Record in Thailand.

Andaman Sea (Wells et al. 2021).

####### Taxonomic remarks and comparisons.

This species is recognised based on the descriptions and figures in Middlefart (1997), specifically in having an erect and crenulate outer lip, which has a pronounced indentation in the subsutural ramp area, and an aperture with four denticles with proceeding lirae present inside.

###### ﻿ Indothais lacera

Taxon classificationAnimaliaNeogastropodaMuricidae

(Born, 1778)

EFF8136B-5771-52BB-B19F-58EDF92F0F8F

Figs 4C , 8A

Murex lacerus Born, 1778: 107–108. Type locality: unknown.
Cymia lacera . Bussarawit 1991: 32. Poutiers 1998b: 561, with in-text figs. Thach 2005: 123, pl. 36, fig. 1.
Thais lacera . Bosch et al. 1995: 123, fig. 491. Middlefart 1997: 369, pl. 4, fig. 4. Subba Rao and Dey 2000: 113. Tan 2000: 499. Swennen et al. 2001: 56, 127, fig. 422. Hylleberg and Kilburn 2003: 76–77. Robba et al. 2003: table 2. Robba et al. 2004: 102, pl. 13, fig. 6a, b. Dharma 2005: 170, pl. 60, fig. 12a–c; 338, pl. 134, fig. 15a–h. Dey 2006: 40–41, figs 45, 46. Ramakrishna et al. 2007: 11, 93, pl. 7, figs 79, 80. Robba et al. 2007: 92 (appendix). Nolf 2009: 9–10, figs 1–14. Kumar et al. 2017: 1101, fig. 1a, b.
Cuma lacera . Nabhitabhata 2009: 143. Yang et al. 2017: 78, fig. 324.
Indothais lacera . Sanpanich and Duangdee 2013: 63. BEDO 2017b: 305, with in-text fig. Kantharajan et al. 2017: table 1, fig. 3-11. Tudu et al. 2018: table 1. Wells et al. 2021: 114.

####### Referred material.

CUF-NKNY-G08 (106 shells; Figs 4C, 8A).

####### Habitat.

Mangrove forests (Kantharajan et al. 2017) and rocky bottom at a depth of 1–25 m (Thach 2005).

####### Distribution.

South Africa to India; Indo-West Pacific, from Japan to New Caledonia (Poutiers 1998b; Robba et al. 2004; Kantharajan et al. 2017; Yang et al. 2017) as well as from western Mediterranean Sea (Nolf 2009). Records of fossils from the Middle Miocene to Quaternary in Indonesia and from the Holocene in Thailand (Robba et al. 2004; Dharma 2005).

####### Record in Thailand.

Gulf of Thailand and Andaman Sea (Wells et al. 2021).

####### Taxonomic remarks and comparisons.

This species is recognised based on the descriptions and figures in Middlefart (1997) and Poutiers (1998b), specifically in having a body whorl bearing one to two spiral ridges, in which the prominent one bears eight spines, and an elaborate crenulate outer lip with a strong incision where the spiral ridge(s) and the anal sulcus are positioned.

***Murex* Linnaeus, 1758**

###### ﻿ Murex trapa

Taxon classificationAnimaliaNeogastropodaMuricidae

Röding, 1798

4222CE6F-5979-5D85-87C4-FB132348C856

Figs 4B , 8B

Murex trapa Röding, 1798: 145. Type locality: Tranquebar, India. Tantanasiriwong 1978: 13, fig. 174. Nateewathana et al. 1981: 59. Way and Purchon 1981: 318. Ponder and Vokes 1988: 41–45, figs 17–19, 67g, h, 71b, c, 73d, 83g, h. Bussarawit 1991: 31. Middelfart 1997: 352, pl. 1, fig. 1. Poutiers 1998b: 565, with in-text figs. Subba Rao and Dey 2000: 102. Tan 2000: 503. Swennen et al. 2001: 55, 126, fig. 410. Hylleberg and Kilburn 2003: 72–73. Robba et al. 2003: table 5. Robba et al. 2004: 96–97, pl. 12, fig. 9a, b. Dharma 2005: 158, pl. 54, fig. 4a, b; 334, pl. 132, fig. 3a–c. Ramakrishna et al. 2007: 10, 85–86. Robba et al. 2007: 92 (appendix). Houart 2008: 136, pl. 636, fig. 2. Nabhitabhata 2009: 147. BEDO 2017b: 312, with in-text fig. Yang et al. 2017: 70, fig. 288. Surakiatchai et al. 2018: table 5, pl. 1, fig. 7a, b. Tudu et al. 2018: table 1. Wells et al. 2021: 116.
Murex cf. trapa . Aungtonya and Hylleberg 1998: 319.
Murex (Murex) trapa . Gemert 2003: 105. Thach 2005: 119, pl. 34, fig. 9. Merle et al. 2011: 59, text-fig. 27e; pl. 4, fig. 7; pl. 5, figs 8–10. Gopalakrishnan et al. 2012: 77. Sanpanich and Duangdee 2013: 63. Okutani 2017: 947, pl. 238, fig. 6.

####### Referred material.

CUF-NKNY-G09 (33 shells; Figs 4B, 8B).

####### Habitat.

Fine sand and muddy sand bottoms at a depth of 5–60 m (Swennen et al. 2001; Robba et al. 2004).

####### Distribution.

Indian Ocean; Indo-West Pacific, from Japan to Fiji Islands (Dharma 2005; Okutani 2017). Records of fossils from the Middle Miocene to Holocene in Indonesia, Malaysia, the Philippines, Taiwan, and Thailand (Ponder and Vokes 1988; Robba et al. 2004; Dharma 2005; Surakiatchai et al. 2018).

####### Record in Thailand.

Gulf of Thailand and Andaman Sea (Wells et al. 2021).

####### Taxonomic remarks and comparisons.

This species differs from other related species in having a higher spire, angulated whorls, and shorter spines (Ponder and Vokes 1988). See also comprehensive taxonomic remarks in Ponder and Vokes (1988).

**Superfamily Conoidea Fleming, 1822**

##### ﻿Family Borsoniidae Bellardi, 1875

***Maoritomella* Powell, 1942**

###### ﻿ Maoritomella vallata

Taxon classificationAnimaliaNeogastropodaBorsoniidae

(Gould, 1860)

B55DD85D-BDFD-5B72-9D3B-3B3591A33277

Figs 4S , 9A

Drillia vallata Gould, 1860: 336. Type locality: Hong Kong. Johnson 1964: 164, pl. 7, fig. 6.
Asthenotoma vallata . Yen 1944: 575, pl. 51, figs 6, 7.
Maoritomella vallata . Robba et al. 2003: table 5. Robba et al. 2004: 134–135, pl. 18, fig. 5a, b. Robba et al. 2007: 94 (appendix). Wells et al. 2021: 100.

####### Referred material.

CUF-NKNY-G30 (5 shells; Figs 4S, 9A).

####### Habitat.

Probably present in shallow sublittoral areas (Robba et al. 2004).

####### Distribution.

Indo-West Pacific. Records of fossils from the Holocene in Thailand (Robba et al. 2004).

####### Record in Thailand.

Gulf of Thailand (Wells et al. 2021).

####### Taxonomic remarks and comparisons.

This species is recognised based on the descriptions and figures in Yen (1944), Johnson (1964), and Robba et al. (2004), specifically in having a high-spired and claviform shell, with the sculpture of one rather strong and peripheral spiral cord, a weaker one abapical to the spiral cord, and one or two spiral threads on the shoulder slope, resulting in a total of 12–14 spirals occurring along the body whorl. This species is poorly known and has been assigned to *Maoritomella* due to a blunt, smooth, and paucispiral protoconch (1.5 whorls in this species), which is the diagnostic character of the genus (Powell 1942).

##### ﻿Family Clathurellidae H. Adams & A. Adams, 1858

***Pseudoetrema* Oyama, 1953**

###### ﻿ Pseudoetrema fortilirata

Taxon classificationAnimaliaNeogastropodaClathurellidae

(Smith, 1879)

B2D38AC1-8810-560B-A11C-E9579E07C259

Figs 4M , 9B

Drillia fortilirata Smith, 1879b: 194, pl. 19, fig. 22. Type locality: Station 14, east of Goto Islands, 32°48'N, 129°6'E; 47 fathoms, and station 21, between south-western extremity of Niphon and the island of Shikoku, 33°45'N, 132°30'E, 30 fathoms. Yokoyama 1927: 393, 410, pl. 46, fig. 20.
Pseudoetrema fortilirata . Oyama 1953: 154. Robba et al. 2004: 141, pl. 19, fig. 6. Robba et al. 2007: 94 (appendix). Okutani 2017: 1017, pl. 311, fig. 7. Wells et al. 2021: 101.

####### Referred material.

CUF-NKNY-G38 (5 shells; Figs 4M, 9B).

####### Habitat.

Sand and sandy mud in sublittoral areas to 50 m depth (Robba et al. 2004; Okutani 2017).

####### Distribution.

Japan and Thailand. Records of fossils from the Pliocene to Holocene in Japan, Taiwan, and Thailand (Robba et al. 2004).

####### Record in Thailand.

Gulf of Thailand (Wells et al. 2021).

####### Taxonomic remarks and comparisons.

This species is recognised based on the descriptions and figures in Smith (1879b), Yokoyama (1927), and Robba et al. (2004), specifically in having a high-spired, claviform shell with the sculpture of nine broad and low collabral ribs fading away shortly before the subsutural cord and overriding spirals, three closely set spiral cords: adapical, peripheral, and abapical cords, and a weaker more adapical one on the penultimate whorl, resulting in a total of 14 spirals occurring along the body whorl. This species is the type species of *Pseudoetrema* (Oyama 1953).

##### ﻿Family Clavatulidae Gray, 1853

***Turricula* Schumacher, 1817**

###### ﻿ Turricula javana

Taxon classificationAnimaliaNeogastropodaClavatulidae

(Linnaeus, 1767)

528B152C-7570-571D-AC15-F15F050211B7

Figs 4I , 8C

Murex javanus Linnaeus, 1767: 1221. Type locality: Java.
Turricula (Vulpecula) javana . Tesch 1915: 48–49, pl. 80, fig. 104a, b.
Turricula javana . Powell 1969: 235–237, pl. 192, figs 10, 11; pl. 201, fig. 11. Tantanasiriwong 1978: 17. Nateewathana et al. 1981: 61. Way and Purchon 1981: 320. Poutiers 1998b: 630, with in-text figs. Swennen et al. 2001: 57, 133, fig. 463. Hylleberg and Kilburn 2003: 104. Robba et al. 2003: tables 3, 5. Robba et al. 2004: 136, pl. 18, fig. 8a, b. Dharma 2005: 130, pl. 40, fig. 8a, b. Thach 2005: 214, pl. 59, fig. 16; pl. 60, fig. 24. Ramakrishna et al. 2007: 15, 133, 134. Robba et al. 2007: 93 (appendix). Nabhitabhata 2009: 196. Sanpanich and Duangdee 2013: 60. BEDO 2017b: 354, with in-text fig. Yang et al. 2017: 114, fig. 472. Surakiatchai et al. 2018: table 5, pl. 1, fig. 11a, b. Tudu et al. 2018: table 1. Wells et al. 2021: 101.

####### Referred material.

CUF-NKNY-G15, G20 (53 shells; Figs 4I, 8C).

####### Habitat.

Intertidal sand and rocks and muddy bottoms at a depth from 20 to 80 m (Robba et al. 2004; Thach 2005).

####### Distribution.

Indian Ocean; Indo-West Pacific, from Japan to Australia (Robba et al. 2004; Yang et al. 2017). Records of fossils from the Late Miocene to Holocene in India, Indonesia, and Thailand (Robba et al. 2004; Surakiatchai et al. 2018).

####### Record in Thailand.

Gulf of Thailand and Andaman Sea (Wells et al. 2021).

####### Taxonomic remarks and comparisons.

This species is recognised based on the descriptions and figures in Powell (1969) and Robba et al. (2004), specifically in having an elongate-fusiform shell with the sculpture of oblique nodes on the peripheral angulation and finer spiral cords over the shoulder slope as well as a twin cord forming a weak subsutural margin. See also comprehensive taxonomic remarks in Powell (1969).

##### ﻿Family Horaiclavidae Bouchet et al., 2011

***Paradrillia* Makiyama, 1940**

###### ﻿ Paradrillia melvilli

Taxon classificationAnimaliaNeogastropodaHoraiclavidae

Powell, 1969

B5B4B526-7D2A-576D-BB6A-57A407E67372

Figs 4N , 9C

Paradrillia melvilli Powell, 1969: 314–315, pl. 242, fig. 2; pl. 245, figs 1, 2. Type locality: Persian Gulf. Robba et al. 2004: 137, 139, pl. 19, fig. 2. Robba et al. 2007: 95 (appendix). Wells et al. 2021: 107.

####### Referred material.

CUF-NKNY-G52 (7 shells; Figs 4N, 9C).

####### Habitat.

Sublittoral and upper bathyal zones (Robba et al. 2004).

####### Distribution.

Persian Gulf and Indian Ocean; Gulf of Thailand. Records of fossils from the Holocene in Thailand (Robba et al. 2004).

####### Record in Thailand.

Gulf of Thailand (Wells et al. 2021).

####### Taxonomic remarks and comparisons.

This species is recognised based on the descriptions and figures in Powell (1969) and Robba et al. (2004), specifically in having a light built claviform shell with a medially situated blunt peripheral keel bearing rather weak cog-like axial nodes, approximately 18 or 19 per whorl.

##### ﻿Family Pseudomelatomidae Morrison, 1966

***Inquisitor* Hedley, 1918**

###### ﻿ Inquisitor vulpionis

Taxon classificationAnimaliaNeogastropodaPseudomelatomidae

Kuroda & Oyama, 1971

5CF6D0E1-75EE-5618-97C8-2B621EF8D88D

Figs 4H , 8D

Inquisitor vulpionis Kuroda & Oyama in Kuroda et al., 1971: 215, pl. 56, fig. 4; pl. 110, fig. 15. Type locality: Sagami Bay, Japan. Robba et al. 2004: 133, pl. 18, fig. 2a–c. Robba et al. 2007: 93 (appendix). Wells et al. 2021: 108.

####### Referred material.

CUF-NKNY-G28 (1 shell; Figs 4H, 8D).

####### Habitat.

Sandy bottoms at a depth from 10 to 100 m (Robba et al. 2004).

####### Distribution.

Japan and the Philippines. Records of fossils from the Holocene in Thailand (Robba et al. 2004).

####### Record in Thailand.

Gulf of Thailand (Wells et al. 2021).

####### Taxonomic remarks and comparisons.

This species is recognised based on the descriptions and figures in Robba et al. (2004), specifically in having a claviform shell with the sculpture of nine collabral ribs resulting in a nodose appearance at the periphery and gradually fading away on the shoulder slope as well as the very faint spiral sculpture all over the shell except for the base where more prominent cords are developed.

***Comitas* Finlay, 1926**

###### ﻿ Comitas ilariae

Taxon classificationAnimaliaNeogastropodaPseudomelatomidae

Bozzetti, 1991

BED32315-90AA-534C-8BC5-1DC74271D466

Figs 4Q , 9D

Comitas ilariae Bozzetti, 1991: 26–28, figs 1–3. Type locality: island of Bohol, central Philippines. Olivera and Sysoev 2008: 786, pl. 688, figs 9, 10.

####### Referred material.

CUF-NKNY-G55 (1 shell; Figs 4Q, 9D).

####### Habitat.

Sandy bottoms at a depth from 100 to 150 m (Bozzetti 1991).

####### Distribution.

Known only from the type locality (Bozzetti 1991).

####### Record in Thailand.

New record in Thailand from this study.

####### Taxonomic remarks and comparisons.

This species is recognised based on the descriptions and figures in Bozzetti (1991) and Olivera and Sysoev (2008), specifically in having a slight, fusiform, and high-spired shell with the axial sculpture made of 7–9 very pronounced, long, and thick tubercles positioned obliquely on each whorl as well as numerous dense spiral cords.

##### ﻿Family Terebridae Mörch, 1852

***Duplicaria* Dall, 1908**

###### ﻿ Duplicaria tricincta

Taxon classificationAnimaliaNeogastropodaTerebridae

(Smith, 1877)

073B37AB-CF31-563D-A70B-2CE920D1BD84

Figs 4O , 10A

Terebra tricincta Smith, 1877: 225. Type locality: Persian Gulf. Bratcher and Cernohorsky 1987: 76, pl. 17, fig. 58a, b. Wilson 1994: 229, with in-text fig. Hylleberg and Kilburn 2003: 106. Robba et al. 2004: 157, pl. 21, fig. 8. Robba et al. 2007: 95 (appendix).
Granuliterebra tricincta . BEDO 2017b: 360, with in-text fig. Okutani 2017: 1050, pl. 339, fig. 14. Tudu et al. 2018: table 1. Wells et al. 2021: 109.
Duplicaria tricincta . Fedosov et al. 2019: 365, fig. 3b. Aubry et al. 2021: pl. 36. Bibi et al. 2021: 51, pl. 1, figs 9–11.

####### Referred material.

CUF-NKNY-G33 (5 shells; Figs 4O, 10A).

####### Habitat.

Sandy mud bottoms at a depth from 10 to 50 m (Robba et al. 2004; Okutani 2017).

####### Distribution.

Persian Gulf; Indo-West Pacific, from Japan to Australia (Robba et al. 2004; Okutani 2017). Records of fossils from the Holocene in Thailand (Robba et al. 2004).

####### Record in Thailand.

Gulf of Thailand and Andaman Sea (Wells et al. 2021).

####### Taxonomic remarks and comparisons.

This species is recognised based on the descriptions and figures in Bratcher and Cernohorsky (1987) and Aubry et al. (2021), and differs from its similar species, *Granuliterebrabathyrhaphe* (Smith, 1875), in having a smaller shell and two additional minutely beaded spiral cords abapical to the subsutural margin and on the lower base.

***Granuliterebra* Oyama, 1961**

###### ﻿ Granuliterebra bathyrhaphe

Taxon classificationAnimaliaNeogastropodaTerebridae

(Smith, 1875)

A0E99B36-6B62-5736-8490-46F67B9BDE50

Figs 4P , 8F

Terebra (Myurella) bathyrhaphe Smith, 1875: 415. Type locality: Gulf of Yedo [Edo Bay, Honshu, Japan].
Terebra sp. Dheeradilok et al. 1984: pl. 3, fig. 11.
Terebra bathyrhaphe . Bratcher and Cernohorsky 1987: 75, pl. 17, fig. 57a–e. Wilson 1994: 223, with in-text fig. Bosch et al. 1995: 171, fig. 765. Robba et al. 2004: 155, pl. 21, fig. 5. Robba et al. 2007: 95 (appendix).
Granuliterebra bathyrhaphe . Okutani 2017: 1049–1050, pl. 339, fig. 13. Fedosov et al. 2019: 379, fig. 10a. Aubry et al. 2021: pl. 73. Bibi et al. 2021: 50, pl. 1, figs 14–17. Wells et al. 2021: 109.

####### Referred material.

CUF-NKNY-G66 (1 shell; Figs 4P, 8F).

####### Habitat.

Muddy and sandy bottoms from intertidal zones to 200 m depth (Robba et al. 2004; Aubry et al. 2021).

####### Distribution.

Persian Gulf and Indian Ocean; Indo-West Pacific, from Japan to Australia (Robba et al. 2004; Okutani 2017; Aubry et al. 2021). Records of fossils from the Quaternary in Japan and Thailand (Robba et al. 2004).

####### Record in Thailand.

Gulf of Thailand (Wells et al. 2021).

####### Taxonomic remarks and comparisons.

This species is recognised based on the descriptions and figures in Bratcher and Cernohorsky (1987) and Aubry et al. (2021), and differs from its similar species, *Duplicariatricincta* (Smith, 1877), by lacking the band anterior to the subsutural band and by having the other two bands that are connected by well-developed ribs rather than by thin cords.

***Pristiterebra* Oyama, 1961**

###### ﻿ Pristiterebra miranda

Taxon classificationAnimaliaNeogastropodaTerebridae

(Smith, 1873)

49A0E1DA-35C4-54E5-9A89-E41F271E48E8

Figs 4D , 8E

Myurella miranda Smith, 1873: 267–268. Type locality: Malacca.
Terebra miranda . Bratcher and Cernohorsky 1987: 78, pl. 18, fig. 63a, b; colour pl. D, fig. 13. Swennen et al. 2001: 57, 134, fig. 471. Robba et al. 2004: 155–156, pl. 21, fig. 6a, b. Dharma 2005: 114, pl. 32, fig. 5a, b. Robba et al. 2007: 95 (appendix).
Pristiterebra miranda . Aubry et al. 2021: pl. 296. Wells et al. 2021: 109.

####### Referred material.

CUF-NKNY-G23 (17 shells; Figs 4D, 8E).

####### Habitat.

At sea depth of 6–10 m (Bratcher and Cernohorsky 1987; Robba et al. 2004).

####### Distribution.

Indo-West Pacific, from Thailand to Indonesia (Bratcher and Cernohorsky 1987; Robba et al. 2004). Records of fossils from the Holocene in Thailand (Robba et al. 2004).

####### Record in Thailand.

Gulf of Thailand (Wells et al. 2021).

####### Taxonomic remarks and comparisons.

This species is recognised based on the descriptions and figures in Bratcher and Cernohorsky (1987) and Aubry et al. (2021), specifically in having a turreted and slightly cyrtoconoid shell with the sculpture completely cancellate throughout and with numerous and unevenly spaced axial cords crossed by ~ 6 spiral cords forming small bead-like nodes at intersections.

**Subclass Heterobranchia Burmeister, 1837**

**Grade “Lower Heterobranchia**”

**Superfamily Architectonicoidea Gray, 1850**

##### ﻿Family Architectonicidae Gray, 1850

***Architectonica* Röding, 1798**

###### ﻿ Architectonica perdix

Taxon classificationAnimaliaNeogastropodaArchitectonicidae

(Hinds, 1844)

A85976DD-52A3-5682-B9C6-0D4B78648F67

Figs 4J , 11A

Solarium perdix Hinds, 1844: 22–23. Type locality: Ceylon; north-west coast of Australia.
Architectonica perdix . Bieler 1993: 48–52, figs 35–38. Swennen et al. 2001: 57, 135, fig. 477. Hylleberg and Kilburn 2003: 113. Robba et al. 2004: 158, pl. 21, fig. 10a, b. Dharma 2005: 204, pl. 77, fig. 2a, b; 358, pl. 144, fig. 7a, b. Thach 2005: 224. Robba et al. 2007: 95 (appendix). Nabhitabhata 2009: 200. BEDO 2017b: 73, with in-text fig. Yang et al. 2017: 118, 120, fig. 493. Surakiatchai et al. 2018: table 5, pl. 1, fig. 12a, b. Tudu and Balakrishnan 2018: 199–200, fig. 1a–f. Wells et al. 2021: 131.

####### Referred material.

CUF-NKNY-G02, G31 (155 shells; Figs 4J, 11A).

####### Habitat.

Sandy and muddy bottoms at a depth from 10 to 60 m (Bieler 1993; Thach 2005; Yang et al. 2017).

####### Distribution.

Indian Ocean; Indo-West to Central Pacific, from China to Australia and Polynesia (Robba et al. 2004; Tudu and Balakrishnan 2018). Records of fossils from the Middle Pliocene in Indonesia and from the Holocene in Thailand (Dharma 2005; Surakiatchai et al. 2018).

####### Record in Thailand.

Gulf of Thailand and Andaman Sea (Wells et al. 2021).

####### Taxonomic remarks and comparisons.

This species differs from its similar species, *Adelphotectonicareevei* (Hanley, 1862), by having a much smaller protoconch and a much higher upper point of attachment of the whorls (Bieler 1993). See also comprehensive taxonomic remarks in Bieler (1993).

**Cohort Tectipleura Schrödl et al., 2011**

**Subcohort Euopisthobranchia Jörger et al., 2010**

**Order Cephalaspidea Fischer, 1883**

**Superfamily Cylichnoidea H. Adams & A. Adams, 1854**

##### ﻿Family Cylichnidae H. Adams & A. Adams, 1854

***Cylichna* Lovén, 1846**

###### ﻿ Cylichna modesta

Taxon classificationAnimaliaCephalaspideaCylichnidae

Thiele, 1925

3A594080-4580-582C-8F78-962675991117

Figs 4R , 10B

Cylichna modesta Thiele, 1925: 241–242 [275–276], pl. 44 [32], fig. 7. Type locality: “Neu-Amsterdam”, Station 167 (37° 47’ südl. Br., 77° 33.7’ östl. L., 496 m). Swennen et al. 2001: 137, fig. 497. Wells et al. 2021: 144.
Adamnestia modesta . Robba et al. 2004: 230, pl. 34, fig. 5a, b. Robba et al. 2007: 97 (appendix). Negri et al. 2014: table 3.

####### Referred material.

CUF-NKNY-G39 (11 shells; Figs 4R, 10B).

####### Habitat.

Sublittoral in muddy or fine sandy bottoms (Robba et al. 2004).

####### Distribution.

Southwest Pacific. Records of fossils from the Holocene in Thailand (Robba et al. 2004).

####### Record in Thailand.

Gulf of Thailand (Wells et al. 2021).

####### Taxonomic remarks and comparisons.

This species differs from its similar species, *Cylichnasibogae* Schepman, 1913, by having a less slender shell and an adapical umbilicus unbounded by sharp angulation (Robba et al. 2004). The generic assignment to either *Adamnestia* Iredale, 1936 or *Cylichna* requires further investigation.

**Subcohort Panpulmonata Jörger et al., 2010**

**Superorder Eupulmonata Haszprunar & Huber, 1990**

**Order Ellobiida Van Mol, 1967**

**Superfamily Ellobioidea Pfeiffer, 1854**

##### ﻿Family Ellobiidae Pfeiffer, 1854

***Ellobium* Röding, 1798**

###### ﻿ Ellobium aurisjudae

Taxon classificationAnimaliaEllobiidaEllobiidae

(Linnaeus, 1758)

77F43895-4FC3-542E-BA83-D8FF10DADB1D

Figs 4G , 11B

Bulla aurisjudae Linnaeus, 1758: 728. Type locality: unknown.
Ellobium aurisjudae . Cernohorsky 1972: 211, pl. 60, fig. 7. Brandt 1974: 227–228, pl. 16, fig. 94. Tantanasiriwong 1978: 20, fig. 256. Nateewathana et al. 1981: 62. Way and Purchon 1981: 321. Bosch et al. 1995: 183, fig. 852. Nateewathana 1995: 100, with in-text fig. Poutiers 1998b: 643, with in-text figs. Subba Rao and Dey 2000: 187. Swennen et al. 2001: 60, 143, fig. 524. Gemert 2003: 107, with in-text fig. Hylleberg and Kilburn 2003: 133. Robba et al. 2003: tables 1, 2. Robba et al. 2004: 241–242, pl. 35, fig. 5. Sri-aroon et al. 2004: table 1. Dharma 2005: 206, pl. 78, fig. 18; 360, pl. 145, fig. 11. Sri-aroon et al. 2005: tables 2, 3, 5, 6. Thach 2005: 232, pl. 71, figs 11, 14. Dey 2006: 56, figs 75, 76. Raven and Vermeulen 2007: 37, 39, pl. 2, figs 10–13. Robba et al. 2007: 98 (appendix). Printrakoon et al. 2008: table 1. Kesavan et al. 2009: 382, with in-text fig. Nabhitabhata 2009: 227. Groh 2010: 446, pl. 914, figs 8, 9. Hamli et al. 2013: tables 2, 3, fig. 2t. Hylleberg and Aungtonya 2013: 97. Sanpanich and Duangdee 2013: 64. Dechruksa et al. 2014: fig. 3e. BEDO 2017b: 81, with in-text fig. Tudu et al. 2018: table 1. Wells et al. 2021: 146.
Ellobium (Ellobium) cf. aurisjudae . Matsubara and Komori 2007: 326–328, fig. 2a–d.
Ellobium (Ellobium) aurisjudae . Matsubara and Komori 2007: fig. 2e–h.

####### Referred material.

CUF-NKNY-G65 (25 shells; Figs 4G, 11B).

####### Habitat.

In estuaries, mangrove and coastal forests, on salt marshes above the normal high tide line in or under rotting wood, and on sandy soil bordering a sandy beach (Raven and Vermeulen 2007).

####### Distribution.

Arabian Gulf and Indian Ocean; Indo-West Pacific, from South China Sea to Australia (Poutiers 1998b; Robba et al. 2004). Records of fossils from the Early Miocene to Holocene in Indonesia, Japan, Malaysia, and Thailand (Robba et al. 2004; Dharma 2005; Matsubara and Komori 2007; Raven and Vermeulen 2007).

####### Record in Thailand.

Gulf of Thailand and Andaman Sea (Wells et al. 2021).

####### Taxonomic remarks and comparisons.

This species is recognised based on the descriptions and figures in Brandt (1974), Poutiers (1998b), and Robba et al. (2004), specifically in having an elongate-oval and unshouldered shell with the sculpture of numerous axial grooves and fine spiral lines.

***Cassidula* Férussac, 1821**

###### ﻿ Cassidula nucleus

Taxon classificationAnimaliaEllobiidaEllobiidae

(Gmelin, 1791)

B7603609-8F63-52D8-8588-865F9B7E0021

Figs 4L , 11C

**Figure 19.**
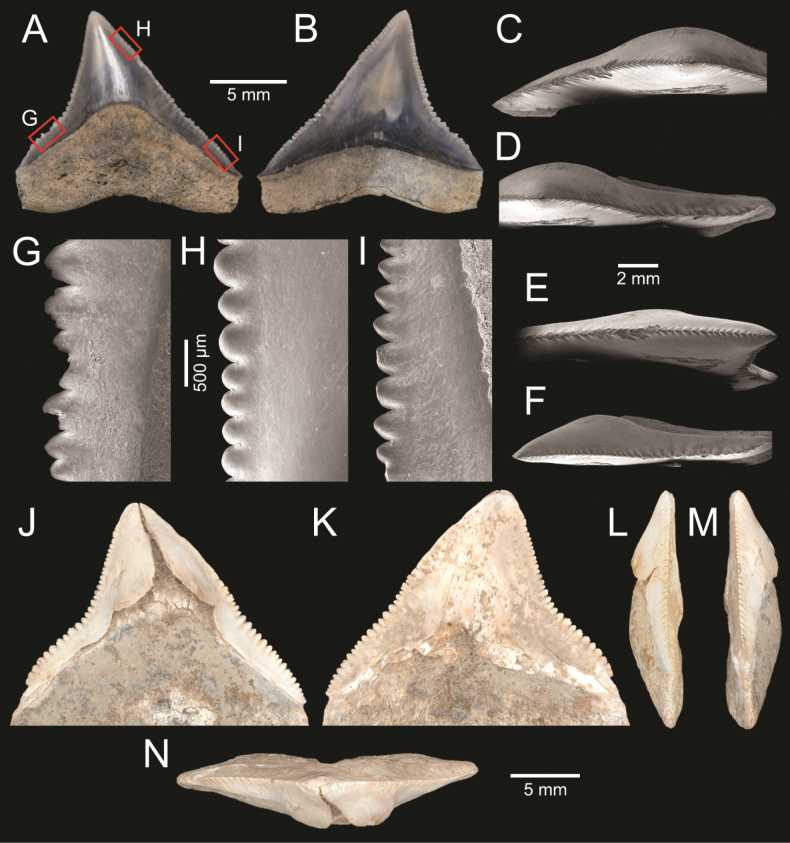
Carcharhinid shark teeth - Carcharhinuscf.leucas**A–I** specimen CUF-NKNY-Q04 in **A** lingual **B** labial and **C–F** different apical views **G–I** close-ups on the serrations of the distal cutting edge at the level of **G** distal heel and mesial cutting edge at the level of **H** upper part of the cusp and **I** heel. **J–N** specimen CUF-NKNY-SA-1 in **J** lingual **K** labial **L** mesial **M** distal and **N** apical views.

**Figure 20.**
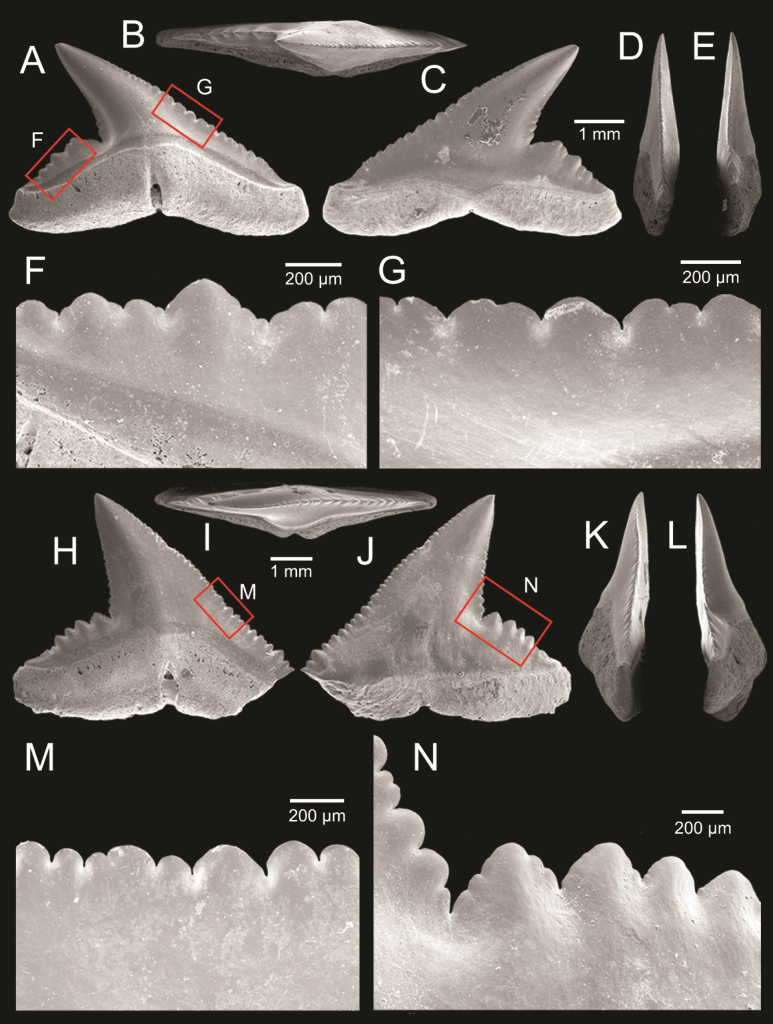
Carcharhinid shark teeth - Carcharhinuscf.sorrah**A–G** specimen CUF-NKNY-3.1 **A** lingual **B** apical **C** labial **D** mesial and **E** distal views **F, G** close-ups of the serration **F** on the distal heel and **G** on the middle part of the cusp mesial cutting-edge **H–N** specimen CUF-NKNY-3.2 **H** lingual **I** apical **J** labial **K** mesial and **L** distal views **M** close-up of the serration on the lower part of the mesial cutting-edge and **N** close-up of the serration between the heel and the base of the distal cutting edge.

**Figure 21.**
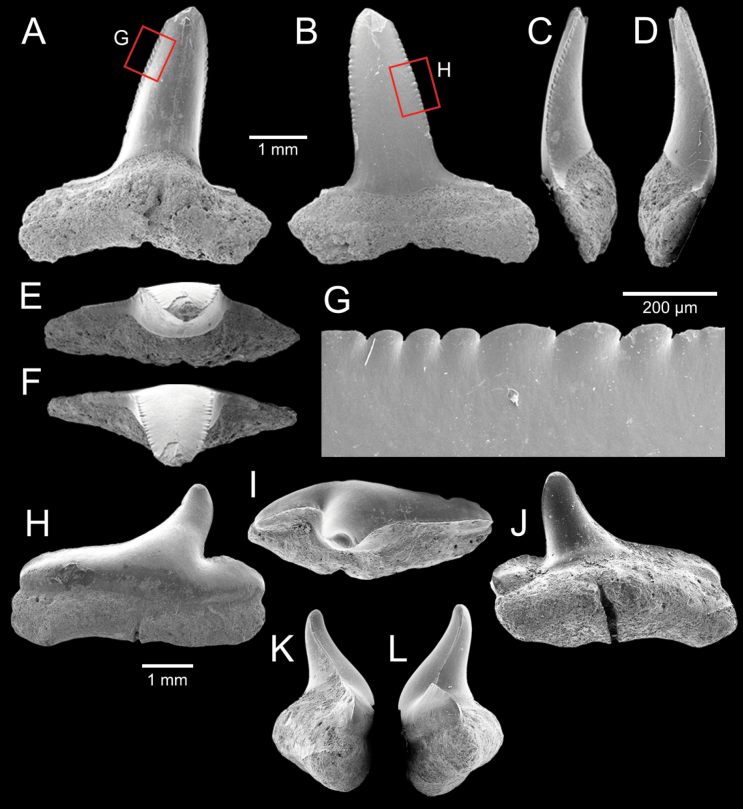
Carcharhinid shark teeth - *Carcharhinus* spp. **A–G** specimen CUF-NKNY-16.1 in **A** lingual **B** labial **C** mesial **D** distal and **E, F** apical views **G** close-ups of the serration on the upper part of the cusp mesial cutting edge in lingual view **H–L** specimen CUF-NKNY-S2 in **H** labial **I** apical **J** lingual **K** mesial and **L** distal views.

**Figure 22.**
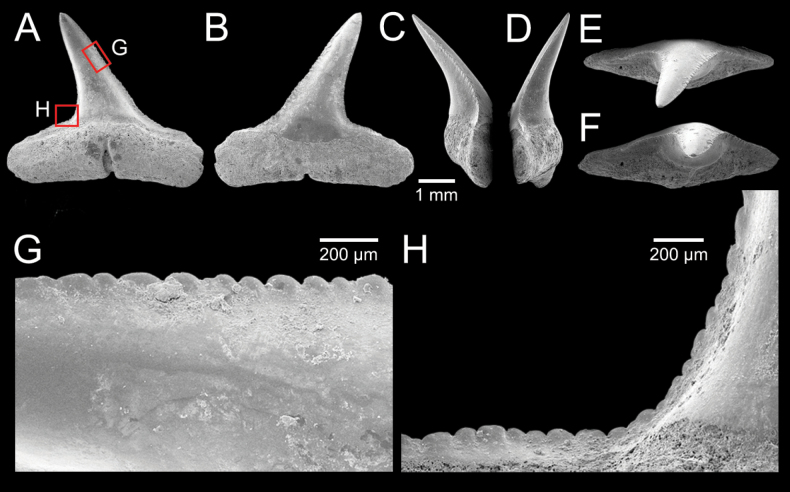
Carcharhinid shark teeth - *Carcharhinus* sp. **A–H** specimen CUF-NKNY-S1 in **A** lingual **B** labial **C** mesial **D** distal and **E, F** apical views **G, H** close-ups on the serration **G** at mid-height of the cusp mesial cutting-edge and **H** at the level of the distal heel and base of the cusp.

**Figure 23.**
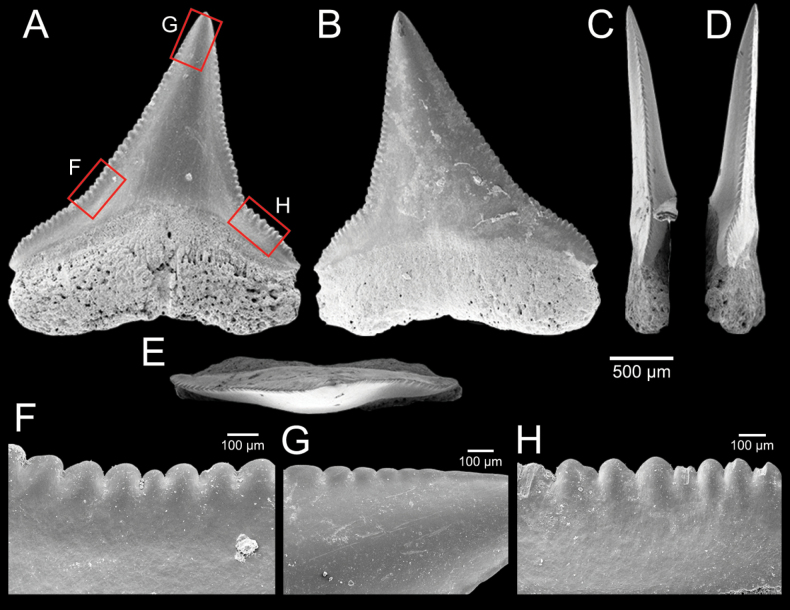
Carcharhinid shark teeth - *Glyphis* sp. specimen CUF-NKNY-8.2, upper lateral tooth in **A** lingual **B** labial **C** mesial **D** distal and **E** apical views **F–H** close-ups of the serration **F** at the base of the cusp mesial cutting-edge, **G** at the tip of the cusp and **H** on the distal heel.

**Figure 24.**
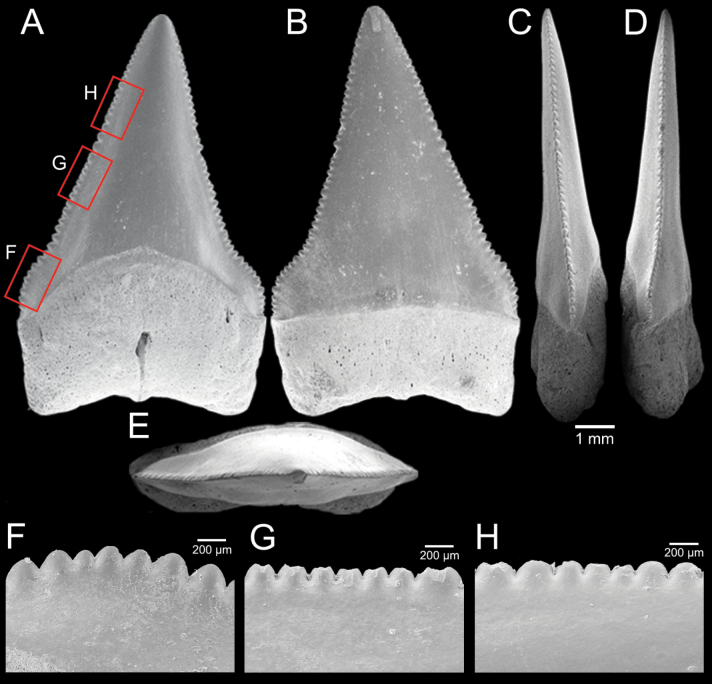
Carcharhinid shark teeth - *Glyphis* sp. specimen CUF-NKNY-Q03, upper anterior tooth in **A** lingual **B** labial **C** mesial **D** distal and **E** apical views **F–H** close-ups of the serration **F** at the base, **G** in the middle and **H** in the upper part of the cusp mesial cutting-edge.

Helix nucleus Gmelin, 1791: 3193. Type locality: “Tahiti” [Cooktown, Queensland, Australia].
Auricula mustelina Deshayes, 1830: 92. Type locality: “New Zealand”.
Cassidula nucleus . Cernohorsky 1972: 212, pl. 60, fig. 8. Bosch et al. 1995: 183, fig. 851. Subba Rao and Dey 2000: 188. Swennen et al. 2001: 60, 143, fig. 526. Hylleberg and Kilburn 2003: 133. Robba et al. 2004: 242–243, pl. 35, fig. 6. Dharma 2005: 208, pl. 79, fig. 1a, b. Thach 2005: 233, pl. 72, fig. 8. Dey 2006: 55–56, figs 73, 74. Raven and Vermeulen 2007: 50–51, pl. 4, figs 35, 36. Robba et al. 2007: 98 (appendix). Kesavan et al. 2009: 382, with in-text fig. Nabhitabhata 2009: 227. Groh 2010: 448, pl. 915, figs 9–11. Zvonareva and Kantor 2016: 429–430, fig. 8s, t. BEDO 2017b: 80, with in-text fig. Kantharajan et al. 2017: table 1, fig. 4-32. Yang et al. 2017: 134, fig. 545. Tudu et al. 2018: table 1. Wells et al. 2021: 145.
Cassidula mustelina . Brandt 1974: 221, pl. 16, fig. 88. Tantanasiriwong 1978: 20, fig. 259. Nateewathana et al. 1981: 62. Way and Purchon 1981: 321. Hylleberg and Kilburn 2003: 133. Sri-aroon et al. 2004: table 1, fig. 2-9. Sri-aroon et al. 2005: tables 2–6. Dechruksa et al. 2014: fig. 3c. BEDO 2017b: 80, with in-text fig. Okutani 2017: 1128, pl. 427, fig. 4.

####### Referred material.

CUF-NKNY-G63 (1 shell; Figs 4L, 11C).

####### Habitat.

On mud in shaded areas and on tree trunks in mangrove and nipa palm forests, sometimes present in salt marsh or on muddy tidal flats (Brandt 1974; Raven and Vermeulen 2007; Zvonareva and Kantor 2016).

####### Distribution.

Arabian Gulf and Indian Ocean; Indo-West Pacific, from Japan to Australia (Brandt 1974; Bosch et al. 1995; Subba Rao and Dey 2000; Okutani 2017). Records of fossils from the Holocene in Thailand (Robba et al. 2004).

####### Record in Thailand.

Gulf of Thailand and Andaman Sea (Brandt 1974; Swennen et al. 2001; Wells et al. 2021).

####### Taxonomic remarks and comparisons.

This species differs from its similar species, *Cassidulaaurisfelis* (Bruguière, 1789), by having a normal and non-bifurcated columellar fold, a less rounded outline with a less convex shoulder, a straighter aperture, and a thinner palatal ridge (Raven and Vermeulen 2007). See also comprehensive taxonomic remarks in Raven and Vermeulen (2007).

﻿**Class Bivalvia Linnaeus, 1758**

**Subclass Protobranchia Pelseneer, 1889**

**Order Nuculanida Carter et al., 2000**

**Superfamily Nuculanoidea H. Adams & A. Adams, 1858**

##### ﻿Family Nuculanidae H. Adams & A. Adams, 1858

***Jupiteria* Bellardi, 1875**

###### ﻿ Jupiteria puellata

Taxon classificationAnimaliaNuculanidaNuculanidae

(Hinds, 1843)

6BC88CB8-4043-57EB-A694-B0F8FE685371

Figs 10C , 12O

Nucula puellata Hinds, 1843: 100. Type locality: Malacca; from 10 to 17 fathoms, coarse sand.
Nuculana puellata . Lynge 1909: 105. Hylleberg and Kilburn 2003: 139.
Nuculana (Jupiteria) puellata . Robba et al. 2002: 53, pl. 1, fig. 4a, b. Robba et al. 2003: tables 1, 2, 4–5. Robba et al. 2007: 83 (appendix). Nabhitabhata 2009: 279. Negri et al. 2014: table 3. Huber 2015: C666.
Jupiteria puellata . Wells et al. 2021: 50.

####### Referred material.

CUF-NKNY-B25 (37 shells; Figs 10C, 12O).

####### Habitat.

Subtidal on coarse sand, soft clay, and mud bottoms at a depth from 5 to 40 m (Huber 2015).

####### Distribution.

Indo-West Pacific, from southern China to New Guinea (Huber 2015). Records of fossils from the Holocene in Thailand (Robba et al. 2002).

####### Record in Thailand.

Gulf of Thailand (Wells et al. 2021).

####### Taxonomic remarks and comparisons.

This species is recognised based on the descriptions and figures in Robba et al. (2002), specifically in having an unsculptured surface bearing only faint growth markings.

***Saccella* Woodring, 1925**

###### ﻿ Saccella mauritiana

Taxon classificationAnimaliaNuculanidaNuculanidae

(Sowerby I, 1833)

2926F175-C064-5BAF-BB63-6ACA28A5252B

Figs 10D , 12P

Nucula mauritiana Sowerby I, 1833: 15, fig. 7. Type locality: Mauritius.
Nuculana mauritiana . Lynge 1909: 105. Swennen et al. 2001: 43, 62, fig. 4. Gopalakrishnan et al. 2012: 61.
Nuculana (Scaeoleda) caspidata [non Gould]. Robba et al. 2002: 53, pl. 1, fig. 3a, b.
Nuculana (Scaeoleda) mauritiana . Robba et al. 2003: tables 1, 3, 5. Robba et al. 2004: 245. Robba et al. 2007: 83 (appendix). Negri et al. 2014: table 3.
Sacella mauritiana [sic]. Nabhitabhata 2009: 278.
Nuculana (Saccella) mauritiana . Huber 2010: 97, with in-text fig. Huber 2015: C612.
Saccella mauritiana . Wells et al. 2021: 50.

####### Referred material.

CUF-NKNY-B24 (20 shells; Figs 10D, 12P).

####### Habitat.

Sublittoral on mainly mud and clay with sand and shells at a depth from 11 to 92 m (Huber 2015).

####### Distribution.

Indian Ocean; Indo-West Pacific, from China to Indonesia (Huber 2015). Records of fossils from the Holocene in Thailand (Robba et al. 2002).

####### Record in Thailand.

Gulf of Thailand and Andaman Sea (Swennen et al. 2001; Nabhitabhata 2009; Wells et al. 2021).

####### Taxonomic remarks and comparisons.

This species is recognised based on the descriptions and figures in Robba et al. (2002) and Huber (2010), specifically in having strong commarginal ridges in the umbonal area then changing into flat and somewhat imbricate low rugae which are more raised on crossing the postero-dorsal keel. This species often co-occurs with *Jupiteriapuellata*, which has an unsculptured shell (Robba et al. 2002).

**Superorder Pteriomorphia Beurlen, 1944**

**Order Arcida Stoliczka, 1871**

**Superfamily Arcoidea Lamarck, 1809**

##### ﻿Family Arcidae Lamarck, 1809

***Anadara* Gray, 1847**

###### ﻿ Anadara inaequivalvis

Taxon classificationAnimaliaArcidaArcidae

(Bruguière, 1789)

6A228285-CA59-5F53-BBBF-6003393E7DFD

Figs 11D , 13D

Arca inaequivalvis Bruguière, 1789: 106–107. Type locality: East India. Reeve 1844: Arca, pl. 8, sp. 54.
Scapharca inaequivalvis . Tantanasiriwong 1979: 4. Morris and Purchon 1981: 322. Nateewathana et al. 1981: 63. Poutiers 1998a: 152, with in-text figs. Subba Rao and Dey 2000: 205. Robba et al. 2002: 58–59, pl. 2, fig. 8. Robba et al. 2003: tables 4, 5. Robba et al. 2007: 83 (appendix). Nabhitabhata 2009: 294. Negri et al. 2014: table 3. Okutani 2017: 1168, pl. 468, fig. 3. Yang et al. 2017: 148, fig. 580.
Anadara (Scapharca) inaequivalvis . Vongpanich 1996: 182, figs 21–23. Aungtonya et al. 1999: 372. Dharma 2005: 242, pl. 96, fig. 10. Dey and Ramakrishna 2007: 151, 174. Huber 2010: 137, with in-text fig. Huber 2015: C3218.
Anadara cf. inaequivalvis . Swennen et al. 2001: 44, 65–66, fig. 29.
Anadara inaequivalvis . Hylleberg and Kilburn 2003: 148. Poppe 2010a: 474, pl. 928, figs 5, 6. Sanpanich 2011: table 2. Gopalakrishnan et al. 2012: 59. Surakiatchai et al. 2018: table 6, pl. 3, fig. 1a, b. Tudu et al. 2018: table 1. Tudu et al. 2019: 38–40, fig. 3c–e. Wells et al. 2021: 52.

####### Referred material.

CUF-NKNY-B03, B21 (21L+30R shells; Figs 11D, 13D).

####### Habitat.

Sandy and muddy bottoms in intertidal and upper sublittoral zones (Robba et al. 2002; Huber 2015).

####### Distribution.

Red Sea to India Ocean; Indo-West Pacific, from Japan to Australia as well as from Mediterranean and Black Sea (Robba et al. 2002; Tudu et al. 2019). Records of fossils from the Late Miocene and Pliocene in Indonesia and from the Holocene in Thailand (Robba et al. 2002; Surakiatchai et al. 2018).

####### Record in Thailand.

Gulf of Thailand and Andaman Sea (Wells et al. 2021).

####### Taxonomic remarks and comparisons.

This species is recognised based on the descriptions and figures in Poutiers (1998a) and Tudu et al. (2019), specifically in having an inflated, inequilateral, and roughly quadrate shell shape with 30–37 radial ribs.

###### ﻿ Anadara indica

Taxon classificationAnimaliaArcidaArcidae

(Gmelin, 1791)

ECE7E204-64DA-5CEB-99DC-CB76C5756820

Figs 11E , 12G

Arca indica Gmelin, 1791: 3312. Type locality: the Indian Ocean. Reeve 1844: Arca, pl. 9, sp. 56.
Anadara (Scapharca) indica . Lynge 1909: 126–127, pl. 2, figs 5–12.
Scapharca indica . Morris and Purchon 1981: 322. Bosch et al. 1995: 211, fig. 928. Poutiers 1998a: 153, with in-text figs. Robba et al. 2002: 59, pl. 2, fig. 9. Gemert 2003: 107. Robba et al. 2003: tables 1–5. Thach 2005: 243, pl. 75, fig. 17. Robba et al. 2007: 83 (appendix). Nabhitabhata 2009: 294. Yang et al. 2017: 148, fig. 581. Surakiatchai et al. 2018: pl. 3, fig. 3a, b.
Anadara indica . Vongpanich 1996: 183, figs 26, 27. Aungtonya et al. 1999: 372. Tudu et al. 2018: table 1. Wells et al. 2021: 52.
Anadara (Anadara) indica . Huber 2010: 136, with in-text fig. Huber 2015: C3220.

####### Referred material.

CUF-NKNY-B06 (16L+24R shells; Figs 11E, 12G).

####### Habitat.

In soft sand or sandy mud bottoms at bays, river mouths, and intertidal zones down to 15 m depth (Huber 2015).

####### Distribution.

Persian Gulf to India Ocean; Indo-West Pacific, from Japan to Australia (Poutiers 1998a; Robba et al. 2002). Records of fossils from the Holocene in Thailand (Robba et al. 2002; Surakiatchai et al. 2018).

####### Record in Thailand.

Gulf of Thailand and Andaman Sea (Wells et al. 2021).

####### Taxonomic remarks and comparisons.

This species is recognised based on the descriptions and figures in Poutiers (1998a), Robba et al. (2002), and Huber (2010), specifically in having an inequilateral, laterally compressed, and subrectangular shell shape with 30–36 flat radial ribs.

***Tegillarca* Iredale, 1939**

###### ﻿ Tegillarca granosa

Taxon classificationAnimaliaArcidaArcidae

(Linnaeus, 1758)

FFAB3EF3-CB5D-5E5B-A8DA-FE700E258556

Figs 11F , 12C

Arca granosa Linnaeus, 1758: 694. Type locality: southern European ocean. Reeve 1844: Arca, pl. 3, sp. 15. Yokoyama 1927: 403. Lutaenko 2015: 135–136, pl. 1, fig. a–d.
Arca (Anadara) granosa . Lynge 1909: 118. Tesch 1920: 92–93, pl. 137, figs 248a, b, 249a–c.
Anadara granosa . Tantanasiriwong 1979: 4. Morris and Purchon 1981: 322. Nateewathana et al. 1981: 63. Nateewathana 1995: 101, with in-text fig. Poutiers 1998a: 147, with in-text figs. Aungtonya et al. 1999: 372. Subba Rao and Dey 2000: 204. Hylleberg and Kilburn 2003: 148, pl. 1, fig. 3. Robba et al. 2002: 57–58, pl. 2, fig. 4. Robba et al. 2003: tables 1–5. Dey 2006: 61, figs 86, 87. Dey and Ramakrishna 2007: 151, 172. Robba et al. 2007: 83 (appendix). Printrakoon et al. 2008: table 1. Nabhitabhata 2009: 284. Sanpanich 2011: table 2. Hylleberg and Aungtonya 2013: 94. Negri et al. 2014: table 3. Surakiatchai et al. 2018: table 6, pl. 2, fig. 7a, b.
Anadara (Tegillarca) granosa . Vongpanich 1996: 187, 189, figs 47–50. Dharma 2005: 242, pl. 96, fig. 9; 362, pl. 146, fig. 10. Lutaenko et al. 2019: 174, pl. 3, fig. c, d.
Tegillarca granosa . Swennen et al. 2001: 44, 66, fig. 35. Gemert 2003: 107. Thach 2005: 244, pl. 75, fig. 8. Huber 2010: 141, with in-text fig. Poppe 2010a: 486, pl. 934, figs 9, 10. Huber 2015: C3352. BEDO 2017a: 27, with in-text fig. Kantharajan et al. 2017: table 1, fig. 2-10. Okutani 2017: 1168, pl. 468, fig. 9. Yang et al. 2017: 150, fig. 586. Tudu et al. 2018: table 1. Tudu et al. 2019: 42–44, fig. 5c, d. Wells et al. 2021: 54.

####### Referred material.

CUF-NKNY-B20 (15L+15R shells; Figs 11F, 12C).

####### Habitat.

Mud down to 10 m depth in mangrove forests and muddy estuaries (Swennen et al. 2001; Thach 2005; Kantharajan et al. 2017).

####### Distribution.

East Africa to India; Indo-West Pacific, from Japan to Australia and Polynesia (Poutiers 1998a; Kantharajan et al. 2017; Tudu et al. 2019). Records of fossils from the Late Miocene to Holocene in Indonesia, Japan, the Philippines, Taiwan, and Thailand (Robba et al. 2002; Dharma 2005; Surakiatchai et al. 2018).

####### Record in Thailand.

Gulf of Thailand and Andaman Sea (Wells et al. 2021).

####### Taxonomic remarks and comparisons.

This species differs from its similar species, *Tegillarcanodifera*, by having a less elongated shell with a lower number of ribs (15–21) and a lower number of nodules on ribs (Tudu et al. 2019).

###### ﻿ Tegillarca nodifera

Taxon classificationAnimaliaArcidaArcidae

(Martens, 1860)

3DEEC620-6B64-52CF-BD03-1C4C05C2C8D6

Figs 12A , 14A

Arca nodifera Martens, 1860: 17. Type locality: Bankok [Bangkok, Thailand].
Anadara nodifera . Nateewathana et al. 1981: 63. Nateewathana 1995: 101, with in-text fig. Poutiers 1998a: 148, with in-text figs. Aungtonya et al. 1999: 372. Hylleberg and Kilburn 2003: 148–149, pl. 1, fig. 5. Nabhitabhata 2009: 284. Hylleberg and Aungtonya 2013: 94.
Anadara (Tegillarca) nodifera . Vongpanich 1996: 189–190, figs 51–54.
Tegillarca nodifera . Swennen et al. 2001: 44, 67, text-fig. 26, fig. 36. Thach 2005: 244, pl. 75, fig. 18. Huber 2010: 141, with in-text fig. Huber 2015: C3366. BEDO 2017a: 28, with in-text fig. Yang et al. 2017: 150, fig. 587. Tudu et al. 2018: table 1. Tudu et al. 2019: 44, fig. 5e, f. Wells et al. 2021: 54.

####### Referred material.

CUF-NKNY-B01, B02, B15 (26L+31R shells; Figs 12A, 14A).

####### Habitat.

On mud and sand bottoms, in intertidal or near mangrove forests and shallow sublittoral waters down to a depth of 10 m (Poutiers 1998a; Huber 2015).

####### Distribution.

Eastern Indian Ocean; Indo-West Pacific, from East China Sea to Indonesia (Poutiers 1998a; Huber 2015; Tudu et al. 2019).

####### Record in Thailand.

Gulf of Thailand and Andaman Sea (Wells et al. 2021).

####### Taxonomic remarks and comparisons.

This species differs from its similar species, *Tegillarcagranosa*, by having a more elongated shell with a higher number of ribs (19–23) and a higher number of nodules on ribs (Tudu et al. 2019).

##### ﻿Family Noetiidae Stewart, 1930

***Estellacar* Iredale, 1939**

###### ﻿ Estellacar olivacea

Taxon classificationAnimaliaArcidaNoetiidae

(Reeve, 1844)

F44C54E0-CBC1-5858-B257-7A9A78657AC2

Figs 12F , 14B

Arca olivacea Reeve, 1844: Arca, pl. 16, sp. 113. Type locality: San Nicolas, island of Zebu [the Philippines] (found in sandy mud at the depth of four fathoms).
Estellacar olivacea . Poutiers 1998a: 160, with in-text figs. Swennen et al. 2001: 44, 68, fig. 42. Robba et al. 2002: 61, pl. 2, fig. 13. Hylleberg and Kilburn 2003: 153. Robba et al. 2003: table 2. Vongpanich and Matsukuma 2004: 41, pl. 2, figs. r, s. Robba et al. 2007: 84 (appendix). Nabhitabhata 2009: 297. Huber 2010: 146, with in-text fig. Negri et al. 2014: table 3. Huber 2015: C3594. BEDO 2017a: 42, with in-text fig. Wells et al. 2021: 55.
Striarca olivacea . Printrakoon et al. 2008: table 1.

####### Referred material.

CUF-NKNY-B18 (16L+14R shells; Figs 12F, 14B).

####### Habitat.

In mud and sand from intertidal zones, often near mangrove forests, down to 20 m depth (Poutiers 1998a; Robba et al. 2002).

####### Distribution.

India; Indo-West Pacific, from South China to the Philippines (Huber 2015). Records of fossils from the Holocene in Thailand (Robba et al. 2002).

####### Record in Thailand.

Gulf of Thailand and Andaman Sea (Wells et al. 2021).

####### Taxonomic remarks and comparisons.

This species differs from its similar species *Estellacargalactodes* (Benson, 1842) by having a higher number of coarser riblets (Robba et al. 2002).

***Noetiella* Thiele, 1931**

###### ﻿ Noetiella pectunculiformis

Taxon classificationAnimaliaArcidaNoetiidae

(Dunker, 1866)

1790E9B3-2CAE-5800-BDEB-1B26F8BB3DD0

Figs 12D , 14C

Barbatia pectunculiformis Dunker, 1866: 88–89, pl. 28, figs 4–6. Type locality: Borneo.
Arca (Fossularca) pectunculiformis . Lynge 1909: 115.
Striarca pectunculiformis . Tantanasiriwong 1979: 4. Morris and Purchon 1981: 323. Nateewathana et al. 1981: 63. Aungtonya et al. 1999: 373.
Scelidionarca pectunculiformis . Robba et al. 2002: 61, pl. 2, fig. 14q, b. Robba et al. 2007: 84 (appendix). Nabhitabhata 2009: 297.
Noetiella pectunculiformis . Huber 2010: 146, with in-text fig. Huber 2015: C3575. BEDO 2017a: 43, with in-text fig. Kantharajan et al. 2017: table 1, fig. 2-11. Wells et al. 2021: 55.

####### Referred material.

CUF-NKNY-B27 (4L+4R shells; Figs 12D, 14C).

####### Habitat.

In soft clay, mud or sand, in subtidal zones from a depth of 2–43 m, and in mangrove forests (Huber 2015; Kantharajan et al. 2017).

####### Distribution.

India Ocean; Indo-West Pacific, from South China to Indonesia (Huber 2015; Kantharajan et al. 2017). Records of fossils from the Holocene in Thailand (Robba et al. 2002).

####### Record in Thailand.

Gulf of Thailand and Andaman Sea (Wells et al. 2021).

####### Taxonomic remarks and comparisons.

This species is recognised based on the descriptions and figures in Robba et al. (2002) and Huber (2010), specifically in having a roundly trapezoidal and inequilateral shell shape with the sculpture of dense, faint radials, and coarse growth markings.

**Order Ostreida Férussac, 1822**

**Superfamily Ostreoidea Rafinesque, 1815**

##### ﻿Family Ostreidae Rafinesque, 1815

***Magallana* Salvi & Mariottini, 2016**

###### ﻿ Magallana cf. gigas

Taxon classificationAnimaliaOstreidaOstreidae

(Thunberg, 1793)

3FCC57DA-878B-5D73-B7BF-A6CFCED4DF21

Figs 12E , 14D

cf. Ostrea gigas Thunberg, 1793: 140–142, pl. 6, figs 1–3. Type locality: Japan. Yokoyama 1927: 402.
cf. Crassostrea gigas . Tantanasiriwong 1979: 7. Nateewathana et al. 1981: 65. Poutiers 1998a: 233, with in-text figs. Aungtonya et al. 1999: 375. Robba et al. 2002: 69, 71, pl. 5, fig. 3a, b. Hylleberg and Kilburn 2003: 162. Beu et al. 2004: 154–156, fig. 9d. Thach 2005: 257. Robba et al. 2007: 84 (appendix). Printrakoon et al. 2008: table 1. Huber 2010: 180, with in-text fig. Sanpanich 2011: table 2. Huber 2015: C5155. Okutani 2017: 1183, pl. 483, fig. 7. Yang et al. 2017: 176, 178, fig. 685. Surakiatchai et al. 2018: table 6, pl. 3, fig. 6a, b. Tudu et al. 2018: table 1.
cf. Crassostrea cf. gigas . Swennen et al. 2001: 45, 72, text-fig. 62, fig. 62.
cf.Crassostrea (Magallana) gigas . Lutaenko et al. 2019: 187–188, pl. 15, fig. a–f.
cf. Magallana gigas . Wells et al. 2021: 60.

####### Referred material.

CUF-NKNY-B13 (130L+16R shells; Figs 12E, 14D).

####### Habitat.

Attached to rocks from intertidal zones down to 30 m depth, in mud, bays, and sheltered areas that are often brackish with low salinity water (Thach 2005; Huber 2015).

####### Distribution.

Cosmopolitan (Huber 2015). Records of fossils from the Miocene to Holocene in Japan, New Zealand, Taiwan, and Thailand (Robba et al. 2002; Beu et al. 2004; Surakiatchai et al. 2018).

####### Record in Thailand.

Gulf of Thailand and Andaman Sea (Wells et al. 2021).

####### Taxonomic remarks and comparisons.

The cupped oysters in the subfamily Crassostreinae could not be identified at species level based on shell characters or soft tissue alone (Salvi and Mariottini 2021). Therefore, we tentatively identify these specimens of giant cupped oysters as belonging to *Magallanagigas*, due to large and thick shells and the presence of this species in Thailand (Wells et al. 2021).

**Order Pectinida Gray, 1854**

**Superfamily Anomioidea Rafinesque, 1815**

##### ﻿Family Placunidae Rafinesque, 1815

***Placuna* Lightfoot, 1786**

###### ﻿ Placuna placenta

Taxon classificationAnimaliaPectinidaPlacunidae

(Linnaeus, 1758)

604AFB1C-1072-59EB-A4CF-014F14112E38

Figs 13A , 14E

Anomia placenta Linnaeus, 1758: 703. Type locality: unknown.
Placuna placenta . Lynge 1909: 107–108. Tantanasiriwong 1979: 6. Morris and Purchon 1981: 324. Nateewathana et al. 1981: 64. Oliver 1992: 86, pl. 17, fig. 6a, b. Bosch et al. 1995: 234, fig. 1018. Poutiers 1998a: 218, with in-text figs. Aungtonya et al. 1999: 376. Subba Rao and Dey 2000: 230. Swennen et al. 2001: 46, 76, text-fig. 80, fig. 80. Robba et al. 2002: 77, pl. 6, fig. 10a, b. Hylleberg and Kilburn 2003: 169–170. Robba et al. 2003: table 5. Dharma 2005: 246, pl. 98, fig. 17. Dey and Ramakrishna 2007: 154, 197. Robba et al. 2007: 84 (appendix). Nabhitabhata 2009: 20, with in-text fig., 347. Huber 2010: 190, with in-text fig. Sanpanich 2011: table 2. Huber 2015: C5627. Yang et al. 2017: 176, fig. 681. Surakiatchai et al. 2018: table 6, pl. 3, fig. 8a, b. Tudu et al. 2018: table 1. Wells et al. 2021: 64.
Placuna (Placuna) placenta . Dheeradilok et al. 1984: pl. 2, fig. 14. Thach 2005: 266, pl. 81, fig. 4.

####### Referred material.

CUF-NKNY-B22 (19L+18R shells; Figs 13A, 14E).

####### Habitat.

Sandy mud in intertidal zones and shallow waters down to 35 m depth, in quiet waters of lagoons, protected bays, and mangrove areas, or near estuaries (Poutiers 1998a; Thach 2005; Yang et al. 2017).

####### Distribution.

Indian Ocean; Indo-West Pacific, from Japan to Australia (Huber 2015). Records of fossils from the Late Miocene to Holocene in Indonesia, Japan, the Philippines, Taiwan, and Thailand (Robba et al. 2002; Surakiatchai et al. 2018).

####### Record in Thailand.

Gulf of Thailand and Andaman Sea (Wells et al. 2021).

####### Taxonomic remarks and comparisons.

This species differs from its similar species, *Placunaephippium* (Philipsson, 1788), by having an almost circular shell and a right valve bearing a hinge plate with arrowhead-shaped lamellar teeth with the posterior ridge distinctly longer than the anterior one (Poutiers 1998a; Swennen et al. 2001).

**Superfamily Pectinoidea Rafinesque, 1815**

##### ﻿Family Pectinidae Rafinesque, 1815

***Volachlamys* Iredale, 1939**

###### ﻿ Volachlamys singaporina

Taxon classificationAnimaliaPectinidaPectinidae

(Sowerby II, 1842)

7B0D5932-8AF5-5EF2-AF0F-0A68112D2C1A

Figs 12B , 14F

Pecten singaporinus Sowerby II, 1842: 74, pl. 13, fig. 55; pl. 14, fig. 71. Type locality: Singapore. Lynge 1909: 155.
Volachlamys singaporina . Roussy 1991: 21. Poutiers 1998a: 210, with in-text figs. Aungtonya et al. 1999: 376. Swennen et al. 2001: 45, 74, fig. 70. Gemert 2003: 108. Hylleberg and Kilburn 2003: 169. Dharma 2005: 248, pl. 99, fig. 13a–e; 364, pl. 147, fig. 3a, b. Thach 2005: 263, pl. 80, fig. 13. Nabhitabhata 2009: 342. Huber 2010: 203, with in-text fig. Huber 2015: C6118. BEDO 2017a: 236, with in-text fig. Yang et al. 2017: 172, fig. 667. Dijkstra and Beu 2018: 296–298, figs 98, 100j, l, 101d, e. Wells et al. 2021: 67.

####### Referred material.

CUF-NKNY-B07 (32L+32R shells; Figs 12B, 14F).

####### Habitat.

Byssally attached under coral boulders and rocks, on sand and sandy mud in intertidal zones down to 22 m depth (Huber 2015).

####### Distribution.

Indo-West Pacific, from China to Australia (Huber 2015; Dijkstra and Beu 2018). Records of fossils from the Middle to Late Pliocene in Indonesia (Dharma 2005).

####### Record in Thailand.

Gulf of Thailand and Andaman Sea (Wells et al. 2021).

####### Taxonomic remarks and comparisons.

This species differs from its similar species, *Volachlamystranquebaria* (Gmelin, 1791) and *V.hirasei* (Bavay, 1904), by having square and well-defined 18–24 ribs with wider interspaces between the ribs (Dharma 2005; Dijkstra and Beu 2018). See also comprehensive taxonomic remarks in Dijkstra and Beu (2018)

**Superorder Heteroconchia Gray, 1854**

**Order Lucinida Gray, 1854**

**Superfamily Lucinoidea J. Fleming, 1828**

##### ﻿Family Lucinidae J. Fleming, 1828

***Pegophysema* Stewart, 1930**

###### ﻿ Pegophysema bialata

Taxon classificationAnimaliaLucinidaLucinidae

(Pilsbry, 1895)

C9597B25-A590-5CD9-9A71-EDC62B83F472

Figs 13E , 15A

Loripes bialata Pilsbry, 1895: 133–134, pl. 3, figs 13, 14. Type locality: Inland Sea, Japan.
Anodontia (?Pegophysema) bialata. Taylor and Glover 2005: 305–306, figs 11a, 12a, 22, 23.
Pegophysema bialata . Huber 2015: 102, 455–456, with in-text figs; C10244. Wells et al. 2021: 35.

####### Referred material.

CUF-NKNY-B11 (3L+3R shells; Figs 13E, 15A).

####### Habitat.

Muddy bottoms and sandy mud among weed in intertidal zones down to 60 m depth (Huber 2015).

####### Distribution.

Indian Ocean; Indo-West Pacific, from Japan to Malaysia (Taylor and Glover 2005; Huber 2015).

####### Record in Thailand.

Gulf of Thailand (Wells et al. 2021).

####### Taxonomic remarks and comparisons.

This species differs from its similar species, *Pegophysemaphilippiana* (Reeve, 1850), by having a ligament that is shallowly inset and an absence of secondary pallial attachment scars (Taylor and Glover 2005). See also comprehensive taxonomic remarks in Taylor and Glover (2005).

**Order Venerida Gray, 1854**

**Superfamily Cyrenoidea Gray, 1840**

##### ﻿Family Cyrenidae Gray, 1840

***Geloina* Gray, 1842**

###### ﻿ Geloina bengalensis

Taxon classificationAnimaliaVeneridaCyrenidae

(Lamarck, 1818)

55AF5496-4A5C-5553-AE3E-23AA1051DF8E

Figs 13C , 15B

Cyrena bengalensis Lamarck, 1818: 554. Type locality: Bengal.
Polymesoda (Geloina) bengalensis . Brandt 1974: 310–311, pl. 28, fig. 83. Nateewathana 1995: 106, with in-text fig. Aungtonya et al. 1999: 380. Robba et al. 2002: 106, pl. 16, fig. 9. Robba et al. 2003: tables 1, 2. Robba et al. 2007: 87 (appendix). Nabhitabhata 2009: 21, with in-text fig, 471.
Polymesoda bengalensis . Poutiers 1998a: 319, with in-text figs. Dharma 2005: 266, pl. 108, fig. 25. Dey 2006: 74–75, figs 113, 114. Tudu et al. 2018: table 1.
Geloina bengalensis . Huber 2015: 319; C20908. BEDO 2017a: 252, with in-text fig. Wells et al. 2021: 39.

####### Referred material.

CUF-NKNY-B31 (2L+3R shells; Figs 13C, 15B).

####### Habitat.

In intertidal zones, river deltas, estuaries, mud flats, and in mangrove areas (Poutiers 1998a; Huber 2015).

####### Distribution.

Bay of Bengal; Indo-West Pacific, from Taiwan to Indonesia (Huber 2015). Records of fossils from the Holocene in Thailand (Robba et al. 2002).

####### Record in Thailand.

Gulf of Thailand and Andaman Sea (Wells et al. 2021).

####### Taxonomic remarks and comparisons.

This species is recognised based on the descriptions and figures in Robba et al. (2002) and Huber (2015), specifically in having a solid, inflated, pronounced inequilateral, and subtriangular shell shape.

**Superfamily Mactroidea Lamarck, 1809**

##### ﻿Family Mactridae Lamarck, 1809

***Lutraria* Lamarck, 1799**

###### ﻿ Lutraria complanata

Taxon classificationAnimaliaVeneridaMactridae

(Gmelin, 1791)

2158F2C1-D81F-5DD7-AA69-8D85DCA95732

Figs 13H , 15C

Mactra complanata Gmelin, 1791: 3261. Type locality: the Indian Ocean.
Lutraria (Lutrophora) cf. complanata . Dheeradilok et al. 1984: pl. 2, fig. 5.
Lutraria complanata . Swennen et al. 2001: 47, 82, fig. 112. Hylleberg and Kilburn 2003: 186. Yang et al. 2017: 194, fig. 742. Wells et al. 2021: 40.
Lutraria (Lutrophora) complanata . Robba et al. 2002: 91, pl. 12, fig. 4. Robba et al. 2007: 86 (appendix). Huber 2010: 451, with in-text figs. Huber 2015: C21984.
Lutraria (Goniomactra) complanata . Beu 2006: 242.
Lutraria (Lutropbora) complanata [sic]. Nabhitabhata 2009: 412.

####### Referred material.

CUF-NKNY-B29 (1L+2R shells; Figs 13H, 15C).

####### Habitat.

Sandy and muddy sand in subtidal zones from a depth of 8–20 m (Huber 2015).

####### Distribution.

Indian Ocean; Indo-West Pacific, from South China to Indonesia (Huber 2015; Yang et al. 2017). Records of fossils from the Holocene in Thailand (Robba et al. 2002).

####### Record in Thailand.

Gulf of Thailand (Wells et al. 2021).

####### Taxonomic remarks and comparisons.

This species is recognised based on the descriptions and figures in Robba et al. (2002) and Huber (2010), specifically in having an elongate-elliptical and markedly inequilateral thin shell with arched anterior and posterior margins, and an outer surface sculptured with irregular crenulated growth lines.

***Standella* Gray, 1853**

###### ﻿ Standella pellucida

Taxon classificationAnimaliaVeneridaMactridae

(Gmelin, 1791)

ADB00F37-FB59-5988-8CAC-2AD50EA7AF7D

Figs 13B , 15D

Mactra pellucida Gmelin, 1791: 3260–3261. Type locality: “Guinea coast.” Sanpanich 2011: table 2.
Standella pellucida . Lynge 1909: 224. Nabhitabhata 2009: 418. Huber 2010: 451, with in-text figs. Huber 2015: C22028. Tudu et al. 2018: table 1. Wells et al. 2021: 42.
Meropesta pellucida . Bosch et al. 1995: 248, fig. 1100. Poutiers 1998a: 281, with in-text figs. Swennen et al. 2001: 47, 83, text-fig. 116, fig. 116. Robba et al. 2002: 91–92, pl. 12, fig. 6. Dey and Ramakrishna 2007: 211. Robba et al. 2007: 86 (appendix). Meyer et al. 2008: tables 2, 3. Nabhitabhata 2009: 417. Wong 2009: 289, figs 9a, b, 22. BEDO 2017a: 155. Yang et al. 2017: 196, fig. 752.

####### Referred material.

CUF-NKNY-B30 (1L+1R shells; Figs 13B, 15D).

####### Habitat.

Fine sand and mud in intertidal zones down to 4 m depth, in lagoons, and at the fringe of mangrove areas (Poutiers 1998a; Huber 2015).

####### Distribution.

Arabian Gulf and Indian Ocean; Indo-West Pacific, from Japan to Australia (Bosch et al. 1995; Wong 2009; Huber 2015). Records of fossils from the Quaternary in Indonesia and from the Holocene in Thailand (Robba et al. 2002).

####### Record in Thailand.

Gulf of Thailand and Andaman Sea (Wells et al. 2021).

####### Taxonomic remarks and comparisons.

This species is recognised based on the descriptions and figures in Robba et al. (2002) and Huber (2010), specifically in having a longer than high, inequilateral, and elliptical shell with an oval-shaped anterior margin and a somewhat pointed posterior margin, an outer surface sculptured with uneven and fine growth markings, and faint posterior radial striation. An exceedingly shallow radial depression is sometimes observed in the mid-anterior part. Huber (2015) indicated that the type locality in Guinea is erroneous.

**Superfamily Tellinoidea Blainville, 1814**

##### ﻿Family Tellinidae Blainville, 1814

***Tellinides* Lamarck, 1818**

###### ﻿ Tellinides conspicuus

Taxon classificationAnimaliaVeneridaTellinidae

(Hanley, 1846)

B02DCE4C-EBE7-5738-8D09-187C9B75344A

Figs 13J , 15E

Tellina conspicua Hanley, 1846b: 293, pl. 58, fig. 100. Type locality: unknown. Coan and Kabat 2012: 309.
Tellina (Tellinides) sp. Robba et al. 2002: 98, pl. 14, fig. 7a, b.
Tellinides conspicuus . Huber 2015: 220, 639, with in-text figs; C14877. Wells et al. 2021: 30.

####### Referred material.

CUF-NKNY-B04 (33L+40R shells; Figs 13J, 15E).

####### Habitat.

Shallow water (Huber 2015).

####### Distribution.

Indo-West Pacific, from southern China to New Guinea (Huber 2015).

####### Record in Thailand.

Gulf of Thailand (Wells et al. 2021).

####### Taxonomic remarks and comparisons.

Tellina (Tellinides) sp. in Robba et al. (2002) was identified as belonging to this species by Huber (2015). This species differs from its similar species, *Tellinidestimorensis* Lamarck, 1818, by having a higher shell that lacks the posterior truncation with oblique anterior striae fading posteriorly (Robba et al. 2002; Huber 2015).

**Superfamily Ungulinoidea Gray, 1854**

##### ﻿Family Ungulinidae Gray, 1854

***Joannisiella* Dall, 1895**

###### ﻿ Joannisiella oblonga

Taxon classificationAnimaliaVeneridaUngulinidae

(Hanley, 1846)

F5532F7F-795F-5614-8B64-B2E9D943E5EB

Figs 12S , 15F

Cyrenoidea oblonga Hanley, 1846a: 10 (plate explanation). Type locality: Philippines. Hanley 1856: 353, pl. 15, fig. 6. Coan and Kabat 2012: 320.
Diplodonta (Joannisiella) oblonga . Lynge 1909: 176.
Cycladicama oblonga . Dheeradilok et al. 1984: pl. 2, figs 6, 7. Robba et al. 2002: 79, pl. 7, fig. 9a, b. Hylleberg and Kilburn 2003: 174. Robba et al. 2003: tables 3, 4. Robba et al. 2007: 85 (appendix). Nabhitabhata 2009: 389. Yang et al. 2017: 182, fig. 704. Surakiatchai et al. 2018: table 6, pl. 3, fig. 10a, b.
Joannisiella oblonga . Huber 2015: 341, 829, with in-text figs; C21436. Wells et al. 2021: 42.

####### Referred material.

CUF-NKNY-B09, B17 (59L+66R shells; Figs 12S, 15F).

####### Habitat.

Muddy bottom with soft clay, muddy sediments on mud flats, and in the shallow waters from intertidal zones down to 36 m depth (Huber 2015; Yang et al. 2017).

####### Distribution.

Indo-West Pacific, from Japan to Indonesia and the Philippines (Huber 2015; Yang et al. 2017). Records of fossils from the Middle Miocene to Holocene in Indonesia, Japan, the Philippines, Taiwan, and Thailand (Robba et al. 2002; Surakiatchai et al. 2018).

####### Record in Thailand.

Gulf of Thailand (Wells et al. 2021).

####### Taxonomic remarks and comparisons.

This species is characterised by its elongate, ovate-triangular shaped, and posteriorly grooved shell (Robba et al. 2002; Huber 2015).

**Superfamily Veneroidea Rafinesque, 1815**

##### ﻿Family Veneridae Rafinesque, 1815

***Dosinia* Scopoli, 1777**

###### ﻿ Dosinia dilecta

Taxon classificationAnimaliaVeneridaVeneridae

A. Adams, 1856

3DE0B5B4-FC56-5190-AC56-0C96DC121636

Figs 12R , 16A

Dosinia dilecta A. Adams, 1856: 224. Type locality: Malacca. Lynge 1909: 249–250, pl. 5, figs 11–13. Morris and Purchon 1981: 326. Swennen et al. 2001: 49, 95, text-fig. 201, fig. 201. Robba et al. 2002: 115, pl. 19, fig. 4. Dharma 2005: 266, pl. 108, fig. 30. Robba et al. 2007: 88 (appendix). Sartori et al. 2008: table 1. Nabhitabhata 2009: 482. Poppe 2010c: 304, pl. 1147, figs 5, 6. Surakiatchai et al. 2018: table 6, pl. 5, fig. 8a, b. Wells et al. 2021: 44.
Dosinia (Dosinella) dilecta . Fischer-Piette and Delmas 1967: 77, pl. 14, figs 1–3. Dheeradilok et al. 1984: pl. 2, figs 9–12. Huber 2010: 414, with in-text fig. Huber 2015: C19500.

####### Referred material.

CUF-NKNY-B10 (2L+5R shells; Figs 12R, 16A).

####### Habitat.

Soft clay and mud bottoms in subtidal zones at a depth from 5 to 20 m (Huber 2015).

####### Distribution.

Indo-West Pacific, from southern China to Indonesia (Huber 2015). Records of fossils from the Holocene in Thailand (Robba et al. 2002; Surakiatchai et al. 2018).

####### Record in Thailand.

Gulf of Thailand and Andaman Sea (Wells et al. 2021).

####### Taxonomic remarks and comparisons.

This species is recognised based on the descriptions and figures in Fischer-Piette and Delmas (1967), Robba et al. (2002), and Huber (2010), specifically in having deeply demarcated and exceedingly small lunulae and a shell with low concentric ribs. See also comprehensive taxonomic remarks in Fischer-Piette and Delmas (1967).

***Paratapes* Stoliczka, 1870**

###### ﻿ Paratapes undulatus

Taxon classificationAnimaliaVeneridaVeneridae

(Born, 1778)

CA2DCA3D-8D68-5F26-B3BE-42087FAE635D

Figs 12Q , 16B

Venus undulata Born, 1778: 54–55. Type locality: unknown.
Tapes (Paratapes) undulatus . Lynge 1909: 237.
Paphia undulata . Tantanasiriwong 1979: 13. Morris and Purchon 1981: 326. Nateewathana et al. 1981: 67. Dheeradilok et al. 1984: pl. 2, fig. 13. Oliver 1992: 191, pl. 43, fig. 6a, b. Bosch et al. 1995: 273, fig. 1227. Nateewathana 1995: 111, with in-text fig. Poutiers 1998a: 339, with in-text figs. Aungtonya et al. 1999: 382. Subba Rao and Dey 2000: 283. Swennen et al. 2001: 49, 95, text-fig. 198, fig. 198. Hylleberg and Kilburn 2003: 217. Dharma 2005: 270, pl. 110, fig. 16; 368, pl. 149, fig. 13. Thach 2005: 300, pl. 90, fig. 3. Dey and Ramakrishna 2007: 160, 244–245. Nabhitabhata 2009: 492. Poppe 2010c: 296, pl. 1143, figs 7–9. Sanpanich 2011: table 2. Okutani 2017: 1248, pl. 543, fig. 4. Yang et al. 2017: 232, fig. 871. Surakiatchai et al. 2018: table 6, pl. 5, fig. 4a, b.
Paphia cf. undulata . Aungtonya and Hylleberg 1998: 320.
Paphia (Paphia) undulata . Gemert 2003: 109. Robba et al. 2002: 113, pl. 18, fig. 8a, b. Robba et al. 2003: tables 1, 3–5. Robba et al. 2007: 88 (appendix). Negri et al. 2014: table 3.
Neotapes undulata . Sartori et al. 2008: 115, table 1, figs 4l, 5l.
Paphia (Neotapes) undulata . Huber 2010: 423, with in-text fig.
Paratapes undulatus . Huber 2015: C19932. Tudu et al. 2018: table 1. Wells et al. 2021: 47.
Paratapes undulata . BEDO 2017a: 281, with in-text fig.

####### Referred material.

CUF-NKNY-B12 (2L shells; Figs 12Q, 16B).

####### Habitat.

Fine sandy sediments and mud as well as silty clay bottoms in intertidal to subtidal zones at a depth of 1–50 m (Huber 2015).

####### Distribution.

Red Sea and Indian Ocean; Indo-West Pacific, from Japan to Australia (Robba et al. 2002; Huber 2015). Records of fossils from the Late Miocene to Holocene in Indonesia, Japan, the Philippines, Taiwan, and Thailand (Robba et al. 2002; Dharma 2005; Surakiatchai et al. 2018).

####### Record in Thailand.

Gulf of Thailand and Andaman Sea (Wells et al. 2021).

####### Taxonomic remarks and comparisons.

Despite having no colour and patterns, the elongate-oval and inequilateral shell shape with growth lines as well as slightly oblique and somewhat undulating commarginal grooves on outer surface of the specimens conform to the characters of this species (Robba et al. 2002; Huber 2010).

***Placamen* Iredale, 1925**

###### ﻿ Placamen lamellatum

Taxon classificationAnimaliaVeneridaVeneridae

(Röding, 1798)

D5E99E32-893A-51B1-9FBD-C64AE70BB1B7

Figs 12L , 16C

Venus lamellata Röding, 1798: 183. Type locality: the East Indian Seas.
Venus tiara Dillwyn, 1817: 162. Type locality: the East Indian Seas.
Venus calophylla Philippi, 1836: 229–230, pl. 8, fig. 2 Type locality: the Chinese Sea.
Chione (Circomphalus) calophylla . Lynge 1909: 150–151.
Callanaitis calophylla . Tantanasiriwong 1979: 13. Nateewathana et al. 1981: 67.
Placamen tiara . Tantanasiriwong 1979: 13. Nateewathana et al. 1981: 67. Poutiers 1998a: 351, with in-text figs. Aungtonya et al. 1999: 382. Hylleberg and Kilburn 2003: 219. Dharma 2005: 268, pl. 109, fig. 24. Thach 2005: 292. Dey and Ramakrishna 2007: 159, 238. Nabhitabhata 2009: 495. Poppe 2010c: 274, pl. 1132, figs 1–3.
Bassina calophylla . Bosch et al. 1995: 266, fig. 1191. Subba Rao and Dey 2000: 284.
Placamen calophyllum . Kilburn and Hylleberg 1998: 315. Aungtonya et al. 1999: 382. Swennen et al. 2001: 48, 92, fig. 180. Robba et al. 2002: 107, pl. 17, fig. 4. Gemert 2003: 15. Hylleberg and Kilburn 2003: 218–219. Dharma 2005: 268, pl. 109, fig. 25. Thach 2005: 292, pl. 89, fig. 11. Robba et al. 2007: 87 (appendix). Meyer et al. 2008: tables 2, 4. Sartori et al. 2008: table 1. Nabhitabhata 2009: 494. Poppe 2010c: 272, pl. 1131, figs 4–7. Sanpanich 2011: table 2.
Placamen lamellatum . Huber 2010: 369, with in-text figs. Huber 2015: C18043. BEDO 2017a: 287. Tudu et al. 2018: table 1. Wells et al. 2021: 48.
Clausinella calophylla . Yang et al. 2017: 222, 224, fig. 834.
Clausinella tiara . Yang et al. 2017: 224, fig. 836.

####### Referred material.

CUF-NKNY-B28 (1R shell; Figs 12L, 16C).

####### Habitat.

Muddy sand, sand, and shell gravels from intertidal zones down to 100 m depth (Bosch et al. 1995; Robba et al. 2002; Yang et al. 2017).

####### Distribution.

Arabian Gulf and Indian Ocean; Indo-West Pacific, from Japan to Indonesia (Bosch et al. 1995; Huber 2015; Yang et al. 2017). Records of fossils from the Late Neogene to Holocene in Fiji, India, Indonesia, Japan, the Philippines and Taiwan (Robba et al. 2002).

####### Record in Thailand.

Gulf of Thailand and Andaman Sea (Wells et al. 2021).

####### Taxonomic remarks and comparisons.

This species differs from its similar species, *Placamenchloroticum* (Philippi, 1849), by having a somewhat shallower and trigonal pallial sinus as well as more widely spaced and more raised commarginal lamellae (Robba et al. 2002).

**Order Myida Stoliczka, 1870**

**Superfamily Myoidea Lamarck, 1809**

##### ﻿Family Corbulidae Lamarck, 1818

***Corbula* Bruguière, 1797**

###### ﻿ Corbula fortisulcata

Taxon classificationAnimaliaMyidaCorbulidae

Smith, 1879

47A4796C-5F26-5F47-9E8C-3298D557E914

Figs 12J , 16D

Corbula fortisulcata Smith, 1879a: 819–820, pl. 50, fig. 23, 23b. Type locality: the Andaman Islands. Subba Rao and Dey 2000: 286. Swennen et al. 2001: 49, 97, text-fig. 212, fig. 212. Hylleberg and Kilburn 2003: 222. Poppe 2010b: 388, pl. 1189, figs 6, 7a, b. Yang et al. 2017: 240, 242, fig. 899. Surakiatchai et al. 2018: table 6, pl. 5, fig. 10a, b. Wells et al. 2021: 36.
Corbula (Corbula) fortisulcata . Robba et al. 2002: 117–118, pl. 20, fig. 5. Robba et al. 2003: table 3. Robba et al. 2007: 88 (appendix). Nabhitabhata 2009: 506.
Corbula (Notocorbula) cf. fortisulcata . Thach 2005: 303, pl. 91, fig. 4.
Corbula (Notocorbula) fortisulcata . Huber 2010: 467, with in-text fig. Huber 2015: C22606.

####### Referred material.

CUF-NKNY-B19 (154 shells; Figs 12J, 16D).

####### Habitat.

Fine sand, sandy mud and muddy bottoms at intertidal to sublittoral zones at a depth from 1 to 70 m (Huber 2015).

####### Distribution.

Indian Ocean; Indo-West Pacific, from Taiwan to Australia (Huber 2015). Records of fossils from the Late Miocene in the Philippines and from the Holocene in Thailand (Robba et al. 2002; Surakiatchai et al. 2018).

####### Record in Thailand.

Gulf of Thailand and Andaman Sea (Wells et al. 2021).

####### Taxonomic remarks and comparisons.

This species differs from its similar species, *Corbulatunicata* Reeve, 1843, by having a weaker keel on the juvenile shell part (Swennen et al. 2001).

***Potamocorbula* Habe, 1955**

###### ﻿ Potamocorbula

Taxon classificationAnimaliaMyidaCorbulidae

sp.

61011B0B-6CEF-5E4D-8FBB-48E578CD8EFC

Figs 12N , 16E

####### Referred material.

CUF-NKNY-B26 (112 shells; Figs 12N, 16E).

####### Habitat.

Mostly present in intertidal zones, estuarine and brackish habitats (Huber 2015).

####### Distribution.

This genus is mostly distributed in the Indian Ocean and Indo-West Pacific, except for the species *Potamocorbulaadusta* (Reeve, 1844), which is recorded only from West Africa (Huber 2015).

####### Taxonomic remarks and comparisons.

These specimens are assigned to *Potamocorbula* based on the descriptions in Coan (2002), specifically in having smooth shells. Currently, there are a total of nine species in this genus (Huber 2015; MolluscaBase 2023), in which two species, *P.fasciata* (Reeve, 1843) and *P.laevis* (Hinds, 1843), are recorded from Thailand (Wells et al. 2021). However, our specimens have a more quadrate shell shape, probably suggesting the presence of other different species.

**Superfamily Pholadoidea Lamarck, 1809**

##### ﻿Family Pholadidae Lamarck, 1809

***Martesia* Sowerby I, 1824**

###### ﻿ Martesia striata

Taxon classificationAnimaliaMyidaPholadidae

(Linnaeus, 1758)

166079A8-F81B-589B-9BC4-16109CC78B8F

Figs 12M , 16F, G

Pholas striatus Linnaeus, 1758: 669. Type locality: southern Europe.
Pholas (Martesia) striata . Lynge 1909: 283–284.
Martesia striata . Cernohorsky 1978: 188, pl. 68, fig. 4. Morris and Purchon 1981: 327. Oliver 1992: 203, pl. 45, fig. 4a, b. Bosch et al. 1995: 279, fig. 1263. Yoosukh and Jitkaew 1997: 402, fig. 1a, b. Poutiers 1998a: 358, with in-text figs. Subba Rao and Dey 2000: 287. Swennen et al. 2001: 49, 99, fig. 223. Hylleberg and Kilburn 2003: 224. Dey and Ramakrishna 2007: 161, 252. Nabhitabhata 2009: 21, with in-text fig, 509. Haga 2010: 394, pl. 1192, figs 3, 4. Gopalakrishnan et al. 2012: 61. Okutani 2017: 1270, pl. 563, fig. 5. Yang et al. 2017: 246, fig. 914. Tudu et al. 2018: table 1. Wells et al. 2021: 37.
Martesia (Martesia) striata . Robba et al. 2002: 121–122, pl. 21, fig. 7a. Thach 2005: 304, pl. 91, fig. 10. Robba et al. 2007: 23, fig. 9p; 88 (appendix). Huber 2010: 476, with in-text figs. Huber 2015: C22956.

####### Referred material.

CUF-NKNY-B14 (8L+8R shells; Figs 12M, 16F, G).

####### Habitat.

Bored in old wood pilings, waterlogged tree trunks, and in driftwood but rarely in astreid corals, in soft rocks, or mangroves *Rhizophorastylosa*; from intertidal zones down to 20 m depth (Huber 2015).

####### Distribution.

Cosmopolitan (Huber 2015). Records of fossils from the Middle Miocene to Holocene in Japan, Indonesia, New Hebrides, and Thailand (Robba et al. 2002).

####### Record in Thailand.

Gulf of Thailand and Andaman Sea (Wells et al. 2021).

####### Taxonomic remarks and comparisons.

This species is recognised based on the descriptions and figures in Poutiers (1998a), Robba et al. (2002), and Huber (2010), specifically in having an outer surface bearing a radial groove at the anterior one-third of the shell, sculptured with commarginal ridges that are minutely nodose and anterior to the radial groove with nodes arranged into radial rows.

***Pholas* Linnaeus, 1758**

###### ﻿ Pholas orientalis

Taxon classificationAnimaliaMyidaPholadidae

Gmelin, 1791

2AFB4845-4BEE-58AC-A5F1-F3CF05DEABE2

Figs 12U , 16H

Pholas orientalis Gmelin, 1791: 3216. Type locality: Siam [Thailand] and Tranquebar. Morris and Purchon 1981: 327. Nateewathana 1995: 111, with in-text fig. Poutiers 1998a: 356, with in-text figs. Subba Rao and Dey 2000: 286. Swennen et al. 2001: 50, 98, fig. 219. Haga 2010: 396, pl. 1193, figs 2, 3. BEDO 2017a: 168, with in-text fig. Hylleberg and Kilburn 2003: 224. Dey and Ramakrishna 2007: 160, 251–252. Nabhitabhata 2009: 510. Gopalakrishnan et al. 2012: 61. Yang et al. 2017: 246, fig. 916. Tudu et al. 2018: table 1. Wells et al. 2021: 37.
Pholas siamensis Spengler, 1792: 88–89. Type locality: the Gulf of Thailand, the mouth of the river Qweda, where it goes up to Alastav. Knudsen and Jensen 2001: 547–556, figs 1–4.
Pholas (Monothyra) orientalis . Lynge 1909: 282. Aungtonya et al. 1999: 383. Robba et al. 2002: 121, pl. 21, fig. 5. Robba et al. 2003: tables 3–5. Dharma 2005: 270, pl. 110, fig. 25; 368, pl. 149, fig. 18a, b. Thach 2005: 304, pl. 91, fig. 6. Robba et al. 2007: 88 (appendix). Huber 2010: 473, with in-text fig. Huber 2015: C22846.

####### Referred material.

CUF-NKNY-B05 (26L+73R shells; Figs 12U, 16H).

####### Habitat.

Boring down to 50 cm depth in peat, soft rocks, stiff clay, or sticky and soft sandy-mud bottoms rich in silt and detritus, often near river mouths, from intertidal and sublittoral zones at a depth from 1 to 30 m (Poutiers 1998a; Huber 2015).

####### Distribution.

Indian Ocean; Indo-West Pacific, from Taiwan to Australia (Huber 2015). Records of fossils from the Middle Miocene to Holocene in Indonesia, Myanmar, and Thailand (Robba et al. 2002; Dharma 2005).

####### Record in Thailand.

Gulf of Thailand and Andaman Sea (Wells et al. 2021).

####### Taxonomic remarks and comparisons.

This species is recognised based on the descriptions and figures in Poutiers (1998a), Swennen et al. (2001), and Robba et al. (2002), specifically in having antero-dorsal reflection that has axial supports.

**Famliy Teredinidae Rafinesque, 1815**

###### ﻿ Teredinidae

Taxon classificationAnimaliaMyidaTeredinidae

indet.

886EC454-E3D4-5F26-81BD-698BBFF0D2D0

Fig. 16I

####### Referred material.

CUF-NKNY-B14-4 (83 tube pieces; Fig. 16I).

####### Habitat.

Boring into submerged wood and other plant material (Poutiers 1998a).

####### Taxonomic remarks and comparisons.

The calcareous burrows have a septum dividing the cavity in half, indicating that these burrows belong to the shipworm family Teredinidae (e.g., Chan and Lau 2021). Due to the absence of shells, it is impossible to assign the inhabitants to species or even genus level based on these trace fossils.

**Order Adapedonta Cossmann & Peyrot, 1909**

**Superfamily Solenoidea Lamarck, 1809**

##### ﻿Family Pharidae H. Adams & A. Adams, 1856

***Cultellus* Schumacher, 1817**

###### ﻿ Cultellus maximus

Taxon classificationAnimaliaAdapedontaPharidae

(Gmelin, 1791)

16B7560F-A473-5E25-81D9-A64F786F49B1

Figs 13G , 17A

Solen maximus Gmelin, 1791: 3227. Type locality: Nicobar.
Solen lacteus Spengler, 1794: 94–95. Type locality: Nicobar.
Cultellus lacteus . Tantanasiriwong 1979: 11. Morris and Purchon 1981: 325. Nateewathana et al. 1981: 66. Aungtonya et al. 1999: 379. Robba et al. 2002: 92, pl. 12, fig. 10. Robba et al. 2007: 86 (appendix). Nabhitabhata 2009: 423.
Cultellus maximus . Subba Rao and Dey 2000: 251. Swennen et al. 2001: 47, 83, text-fig. 121, fig. 121. Huber 2010: 271, with in-text fig. Negri et al. 2014: table 3. Huber 2015: C12478. BEDO 2017a: 48, with in-text fig. Tudu et al. 2018: table 1. Wells et al. 2021: 18.

####### Referred material.

CUF-NKNY-B23 (6L+3R shells; Figs 13G, 17A).

####### Habitat.

Deeply (down to 40 cm) buried in soft mud at seaward fringes of mangrove forests or intertidal zones down to 3 m depth (Huber 2015).

####### Distribution.

Indian Ocean; Indo-West Pacific, from Taiwan to Borneo Island (Huber 2015). Records of fossils from the Holocene in Thailand (Robba et al. 2002).

####### Record in Thailand.

Gulf of Thailand and Andaman Sea (Wells et al. 2021).

####### Taxonomic remarks and comparisons.

Due to the large size and oblong-elliptical shape of shells (Robba et al. 2002; Huber 2010), we identified these specimens as belonging to *Cultellusmaximus*.

***Siliqua* Megerle von Mühlfeld, 1811**

###### ﻿ Siliqua minima

Taxon classificationAnimaliaAdapedontaPharidae

(Gmelin, 1791)

FD019B7C-72B3-5B9D-B756-4298654E905F

Figs 12I , 17B

Solen minimus Gmelin, 1791: 3227. Type locality: Tranquebar.
Siliqua minima . Lynge 1909: 278. Swennen et al. 2001: 47, 84, text-fig. 126, fig. 126. Robba et al: 2002: 93, pl. 12, fig. 11a, b. Robba et al. 2003: tables 3–5. Robba et al. 2007: 86 (appendix). Nabhitabhata 2009: 424. Negri et al. 2014: table 3. Yang et al. 2017: 218, fig. 822. Wells et al. 2021: 19.
Siliqua cf. minima . Hylleberg and Kilburn 2003: 195–196, pl. 8, fig. 10. Thach 2005: 281.
Siliqua (Neosiliqua) minima . Huber 2010: 273, with in-text fig. Huber 2015: C12603.

####### Referred material.

CUF-NKNY-B16 (6L+6R shells; Figs 12I, 17B).

####### Habitat.

Sand and mud bottoms from intertidal zones down to 30 m depth (Huber 2015).

####### Distribution.

Indian Ocean; Indo-West Pacific, from Japan to the Philippines (Robba et al. 2002; Huber 2015). Records of fossils from the Holocene in Thailand (Robba et al. 2002).

####### Record in Thailand.

Gulf of Thailand (Wells et al. 2021).

####### Taxonomic remarks and comparisons.

This species is recognised based on the descriptions and figures in Robba et al. (2002) and Huber (2010), specifically in having an ovate-rectangular and inequilateral shell with a widely rounded and deep pallial sinus, the lower border of which coincides with the pallial line, and a thin and slightly oblique inner rib.

﻿**Class Scaphopoda Bronn, 1862**

**Order Dentaliida Starobogatov, 1974**

##### ﻿Family Dentaliidae Children, 1834

***Dentalium* Linnaeus, 1758**

###### ﻿ Dentalium variabile

Taxon classificationAnimaliaDentaliidaDentaliidae

Deshayes, 1826

5FA3AAC2-469B-5BF8-8DCF-4AE2AD5A9D8F

Figs 12H , 17C

Dentalium variabile Deshayes, 1826: 352–353, pl. 16, fig. 30. Type locality: possibly India. Scarabino 1995: 200–201, fig. 16d, e. Robba et al. 2003: tables 1, 3–5. Robba et al. 2004: 13, pl. 1, fig. 1. Steiner and Kabat 2004: 660. Chaiwathee et al. 2007: table 1. Robba et al. 2007: 89 (appendix). Sahlmann and Poppe 2010: 406, pl. 1198, fig. 8. Negri et al. 2014: tables 3, 4. Wells et al. 2021: 164.
Dentalium (Lentigodentalium) variabile . Dey and Ramakrishna 2007: 162, 257.

####### Referred material.

CUF-NKNY-O01 (48 shells; Figs 12H, 17C).

####### Habitat.

Mud bottoms from a depth of 10–75 m (Robba et al. 2004).

####### Distribution.

Indo-West Pacific, from Japan to Reunion Island and New Caledonia. Records of fossils from the Quaternary in Thailand (Robba et al. 2004).

####### Record in Thailand.

Gulf of Thailand (Wells et al. 2021).

####### Taxonomic remarks and comparisons.

This species is recognised based on the descriptions and figures in Scarabino (1995) and Robba et al. (2004), specifically in having a polygonal cross-section of apex and a circular aperture. See also comprehensive taxonomic remarks in Scarabino (1995).

#### ﻿Phylum Arthropoda

**Subphylum Crustacea Brünnich, 1772**

﻿**Class Malacostraca Latreille, 1802**

**Order Decapoda Latreille, 1802**

##### ﻿Family Thalassinidae Latreille, 1831

***Thalassina* Latreille, 1806**

###### ﻿ Thalassina

Taxon classificationAnimaliaDecapodaThalassinidae

sp.

69AD01A2-762F-5798-82F7-8EAEDD689F08

Figs 13I , 17D

####### Referred material.

CUF-NKNY-O09 (130 pieces; Figs 13I, 17D).

####### Habitat.

Littoral and sublittoral zones, mangrove swamps and forests, and edges of estuaries (Ngoc-Ho and de Saint Laurent 2009).

####### Distribution.

Indo-West Pacific, from Japan to Australia (Hyžný and de Angeli 2022). Records of fossils from the Miocene to Holocene in Indo-West Pacific, and from the Oligocene in Italy (Hyžný and de Angeli 2022).

####### Taxonomic remarks and comparisons.

These specimens are assigned to *Thalassina* based on descriptions in Ngoc-Ho and de Saint Laurent (2009), Sakai and Türkay (2012), and Hyžný and de Angeli (2022), specifically in having a subchelate pereiopod 1 with its propodus having dorsomesially tuberculated or spinuous carinae. There is a total of 13 species within this genus (Hyžný and de Angeli 2022), in which three species, *T.anomala* (Herbst, 1804), *T.gracilis* Dana, 1852, and *T.squamifera* De Man, 1915, are recorded from Thailand (Ngoc-Ho and de Saint Laurent 2009; Sakai and Türkay 2012) (but see the comment on the record of *T.squamifera* in Thailand in Moh et al. (2013)). However, the pereiopod 1 observed in our specimens is shorter and has a more quadrate shape. The dactylus that has a hooked tip is nearly as long as the fixed finger, suggesting an as yet unidentified species.

﻿**Class Thecostraca Gruvel, 1905**

**Subclass Cirripedia Burmeister, 1834**

**Order Balanomorpha Pilsbry, 1916**

##### ﻿Family Balanidae Leach, 1817

**Subfamily Amphibalaninae Pitombo, 2004**

***Fistulobalanus* Zullo, 1984**

###### ﻿ Fistulobalanus kondakovi

Taxon classificationAnimaliaBalanomorphaBalanidae

(Tarasov & Zevina, 1957)

D8017453-C481-522D-B58C-5272C75BB477

Figs 12K , 17E

Balanus amphitrite var. kondakovi Tarasov & Zevina, 1957: 179, 191, fig. 76a–d. Type locality: Japan.
Balanus kondakovi . Henry and Mclaughlin 1975: 114–123, text-figs 21, 22b, c, f; pl. 2, figs a–m. Yamaguchi 1977a: pl. 19, figs 6, 7; pl. 20, figs 4, 8, 12; pl. 21, fig. 4; pl. 22, figs 13–18. Yamaguchi 1977b: 176, 178, text-fig. 18. Kim and Kim 1980: 176, pl. 6, figs 1–8. Yamaguchi 1980: tables 1, 2. Jones et al. 2000: 279. Puspasari et al. 2000: tables 1, 2. Kim 2011: 119–121, fig. 64.
Balanus amphitrite kondakovi . Rosell 1973: 88, fig. 8a–j. Newman and Ross 1976: 63.
Fistulobalanus kondakovi . Zullo 1984: 1330. Pitombo 2004: 275. Prabowo and Yamaguchi 2005: table 3, fig. 3f. Chan et al. 2009: 248–251, figs 214–217. Jones and Hosie 2016: 287–288. Kepel’ 2018: 50–51, fig. 1. Kim 2020: 75–78, figs 48, 49, table 2. Karasawa and Kobayashi 2022: 71, pl. 5, fig. 10a, b.

####### Referred material.

CUF-NKNY-O06-1 (217 individuals; Figs 12K, 17E).

####### Habitat.

Attached to hard substrates, mainly shells of oysters and gastropod molluscs and stalks of seaweed, as well as wooden piles and supports, bamboo, various objects installed under water on muddy beds for oyster culture, and buoys, in freshened closed inner parts of bays and estuarine coastal areas, from intertidal to subtidal zones (Henry and Mclaughlin 1975; Chan et al. 2009; Kepel’ 2018).

####### Distribution.

Indian Ocean; Indo-West Pacific, from Japan to New Zealand (Henry and Mclaughlin 1975; Puspasari et al. 2000; Jones and Hosie 2016). Records of fossils from the Pleistocene and Holocene in Japan (Yamaguchi 1977b; Karasawa and Kobayashi 2022).

####### Record in Thailand.

Gulf of Thailand (Henry and Mclaughlin 1975; Puspasari et al. 2000).

####### Taxonomic remarks and comparisons.

The subfamily Amphibalaninae was erected to incorporate most of the species under the *Balanusamphitrite* complex/group (Pitombo 2004). Currently, there are a total of six species from this subfamily recorded from Thailand: *Amphibalanusamphitrite* (Darwin, 1854), *A.reticulatus* (Utinomi, 1967), *A.thailandicus* (Puspasari, Yamaguchi & Angsupanich, 2001), *A.variegatus* (Darwin, 1854), *Fistulobalanuskondakovi* (Tarasov & Zevina, 1957), and *F.patelliformis* (Bruguière, 1789) (Henry and Mclaughlin 1975; Puspasari et al. 2000, 2001; Pochai et al. 2017). The presence of two or more rows of parietal tubes on shell walls assigns these specimens to the genus *Fistulobalanus* (Zullo 1984; Chan et al. 2009). We attribute these specimens to *F.kondakovi* because of the smooth outer surface of shells without any longitudinal ribs (Henry and Mclaughlin 1975; Chan et al. 2009; Kim 2011; Kepel’ 2018).

**Subfamily Megabalaninae Leach, 1817**

***Megabalanus* Hoek, 1913**

###### ﻿ Megabalanus cf. tintinnabulum

Taxon classificationAnimaliaBalanomorphaBalanidae

(Linnaeus, 1758)

DD1F6532-D76E-5BCF-94C8-3115E08B5FC0

Figs 13F , 17F

cf. Lepas tintinnabulum Linnaeus, 1758: 668. Type locality: Amboina, Indonesia [lectotype designation by Henry and McLaughlin (1986)].
cf.Balanus (Megabalanus) tintinnabulum tintinnabulum . Pilsbry 1916: 55–57, pl. 10, fig. 1, 1e. Hiro 1939: 258, fig. 7a–b.
cf. Balanus tintinnabulum var. tintinnabulum . Oliveira 1941: 11–14, text-fig. 1, pl. 2, figs. 1, 2; pl. 4, fig. 1; pl. 5, fig. 3; pl. 8, fig. 6.
cf. Balanus tintinnabulum . Holthuis and Heerebout 1972: 24–31, pl. 1, fig. a–e.
cf. Megabalanus tintinnabulum . Newman and Ross 1976: 68. Henry and McLaughlin 1986: 17–21, figs 1e, 2a, g, h, 3a–c, 5a–l. Jones et al. 2000: 282. Pitombo 2004: 275. Lozano-Cortés and Londoño-Cruz 2013: 466–467. Jones and Hosie 2016: 289–290. Pochai et al. 2017: 28–29, fig. 11

####### Referred material.

CUF-NKNY-O06-2 (2 individuals; Figs 13F, 17F).

####### Habitat.

Attached on low exposed rocky shores in littoral areas, ship bottoms, and floating pontoons (Chan et al. 2009; Jones and Hosie 2016)

####### Distribution.

Cosmopolitan (Jones and Hosie 2016).

####### Record in Thailand.

Andaman Sea (Pochai et al. 2017). This is the first record of this species from Gulf of Thailand.

####### Taxonomic remarks and comparisons.

These specimens are tentatively identified as belonging to *Megabalanustintinnabulum* based on the descriptions and figures in Henry and McLaughlin (1986), Chan et al. (2009), and Pochai et al. (2017), specifically in having large cylindrical shells with smooth parietal surfaces and without spines.

#### ﻿Phylum Echinodermata

﻿**Class Echinoidea Schumacher, 1817**

**Order Camarodonta Jackson, 1912**

##### ﻿Family Temnopleuridae Agassiz, 1872

***Temnotrema* Agassiz, 1864**

###### ﻿ Temnotrema siamense

Taxon classificationAnimaliaBalanomorphaTemnopleuridae

(Mortensen, 1904)

4AE1C2E0-56B4-5B3A-8695-C152F62CE1C0

Figs 12T , 17G

Pleurechinus siamensis Mortensen, 1904: 79–82, pl. 1, figs 2, 7, 11, 20; pl. 2, figs 2, 9, 14, 15, 22; pl. 6, figs 16, 36; pl. 7, figs 14, 44, 53. Type locality: Koh Mesan, 3–15 fathoms; Koh Chuen, 15–38 faths.; Koh Kram, 20–30 faths.; Koh Kahdat, 10 faths.
Temnotrema siamense . Clark 1925: 92. Mortensen 1940: 45. Mortensen and Gislén 1940: 103. Mortensen 1943: 259–262, fig. 139. Clark and Rowe 1971: 155. Price 1981: 8. Liao 1998: table 1. Lane et al. 2000: 485. Samyn 2003: table 2. Marsh and Morrison 2004: tables 1, 2, 5. Gopalakrishnan et al. 2012: 32. Filander and Griffiths 2014: 53. Schultz 2015: 258, 263–264, fig. 4.169b, c. Arachchige et al. 2017: table 3. Putchakarn 2018: 57, table 1. Arachchige et al. 2019: table 1. Köhler 2020: 9, 72–73, pl. 14, figs 11, 14. Mucharin and Sumitrakij 2022: 6, pl. 4, fig. 1.
Temnotrema siamensis . Putchakarn and Sonchaeng 2004: table 1.

####### Referred material.

CUF-NKNY-O02 (23 shells; Figs 12T, 17G).

####### Habitat.

Coarse and high energy subtidal sand from a depth of 5–350 m (Price 1981; Schultz 2015).

####### Distribution.

Persian Gulf and Indian Ocean; Indo-West Pacific, from South China Sea to Australia (Mortensen 1904; Price 1981; Schultz 2015; Arachchige et al. 2019).

####### Record in Thailand.

Gulf of Thailand and Andaman Sea (Putchakarn and Sonchaeng 2004).

####### Taxonomic remarks and comparisons.

This species is recognised based on the descriptions and figures in Mortensen (1904), Clark and Rowe (1971) and Schultz (2015), specifically in having deep horizontal grooves in ambulacral and interambulacral plates and in lacking a horizontal series of tubercles.

#### ﻿Phylum Chordata

**Subphylum Vertebrata Lamarck, 1801**

**Class Chondrichthyes Huxley, 1880**

**Subcohort Selachimorpha Nelson, 1984**

**Superorder Galeomorphii Compagno, 1973**

**Order Carcharhiniformes Compagno, 1977**

##### ﻿Family Carcharhinidae Jordan & Evermann, 1896

***Carcharhinus* Blainville, 1816**

###### ﻿ Carcharhinus cf. amblyrhynchoides

Taxon classificationAnimaliaCarcharhiniformesCarcharhinidae

(Whitley, 1934)

8B09989A-9F12-503D-8C03-619A738CCDF3

Fig. 18A–E

cf. Gillisqualus amblyrhynchoides Whitley, 1934: 189–191, text-fig. 4. Type locality: Cape Bowling Green, Queensland.
cf. Carcharhinus amblyrhynchoides . Compagno 1984: 458–459, with in-text figs. Krajangdara et al. 2022: 49, with in-text figs.

####### Referred material.

CUF-NKNY-S3-2 (Fig. 18A–E) (1 upper tooth).

####### Description.

The crown of CUF-NKNY-S3-2 is triangular and erect with fine serrations and displays well-developed heels mesially and distally. Its lingual face is distinctly more convex than the labial one. Its base presents a damaged lingual face.

####### Habitat.

Tropical, inshore and offshore, coastal-pelagic species, found over the continental and insular shelves (Compagno 1984).

####### Distribution.

Gulf of Aden and Indian Ocean; Indo-West Pacific, from southern China to Australia (Compagno 1984).

####### Record in Thailand.

Gulf of Thailand and Andaman Sea (Compagno 1984; Krajangdara et al. 2022).

####### Taxonomic remarks and comparisons.

The specimen CUF-NKNY-S3-2 represents an anterior upper tooth. Several species of *Carcharhinus* have similar upper teeth in terms of morphology. Additional teeth and larger assemblages are needed for a more precise identification. Nevertheless, the tooth most resembles the upper teeth of *C.amblyrhynchoides* (Garrick 1982: fig. 20). Male individuals of *C.brachyurus* have somewhat similar upper teeth (Garrick 1982: fig. 51), but the mesial cutting edge of their crown is often more convex. *Carcharhinuslimbatus* also shows the same characters, but the serration on the cusps at the base of the crown is much finer (Bass et al. 1973: pl. 5; Garrick 1982: fig. 18). The pattern of serration also resembles that of the lower teeth of *C.sorrah*, although in the latter, the base of the root is more concave and the heels of the crown are better developed, the teeth being longer mesio-distally than high baso-apically (Voigt and Weber 2011). Regarding the fossil record in Southeast Asia, similar teeth have been reported from the Late Miocene deposits of Brunei in Borneo (Kocsis et al. 2019).

###### ﻿ Carcharhinus cf. amblyrhynchos

Taxon classificationAnimaliaCarcharhiniformesCarcharhinidae

(Bleeker, 1856)

D06B8C38-5391-5FB5-99A3-61708ABF306E

Fig. 18F–J

cf.Carcharias (Prionodon) amblyrhynchos Bleeker, 1856: 467–468. Type locality: Java Sea near Solombo Island.
cf. Carcharhinus amblyrhynchos . Compagno 1984: 459–461, with in-text figs. Krajangdara et al. 2022: 49, with in-text figs.

####### Referred material.

CUF-NKNY-S3-10 (Fig. 18F–J) (1 upper tooth).

####### Description.

The crown of CUF-NKNY-S3-10 displays a rather narrow main cusp with well-developed heels. The main cusp is regularly serrated, with an almost straight mesial edge and a distal one that is slightly concave. It is thus inclined distally. The labial side of the main cusp is nearly flat, whereas the lingual one is convex. Both the mesial and distal heels of the crown are poorly preserved and their serration pattern cannot thus be observed. The base of the root is concave and the nutritive groove on the bulged lingual face is poorly developed or heavily worn. The labial face of the root is nearly flat.

####### Habitat.

Continental and insular shelves and adjacent oceanic waters (Compagno 1984).

####### Distribution.

Indo-western to Central Pacific (Compagno 1984).

####### Record in Thailand.

Gulf of Thailand and Andaman Sea (Compagno 1984; Krajangdara et al. 2022).

####### Taxonomic remarks and comparisons.

The general shape of the crown, the regular serration of the main cusp, and the arched root are reminiscent of *C.amblyrhynchos* (Bass et al. 1973; Garrick 1982; Kocsis et al. 2019; Malyshkina et al. 2023), but the poorly preserved heels of the teeth make a definite identification difficult to ascertain.

###### ﻿ Carcharhinus cf. leucas

Taxon classificationAnimaliaCarcharhiniformesCarcharhinidae

(Valenciennes, 1839)

E7690BD5-42BA-5654-9AA1-C756636609DE

Fig. 19

cf.Carcharias (Prionodon) leucas Valenciennes in Müller & Henle, 1839: 42–43. Type locality: Antilles.
cf. Carcharhinus leucas . Compagno 1984: 478–481, with in-text figs. Krajangdara et al. 2022: 52, with in-text figs.

####### Referred material.

CUF-NKNY-Q04 (Fig. 19A–I), CUF-NKNY-SA-1 (Fig. 19J–N), CUF-NKNY-SB-3, CUF-NKNY-SC-5 (4 upper teeth).

####### Description.

Crowns show well-developed serrations, becoming larger and more complex in the basal part of the distal side of the crown. In labial or lingual view, the mesial side is almost straight, whereas the distal one is concave in its lower third. The outline of the base of the crown is more concave on the lingual face than on the labial one. The root is deep, slightly concave at its base, and does not display a nutritive groove in lingual view.

####### Habitat.

Close inshore in reef-associated marine habitats, mostly in water less than 30 m depth (Compagno 1984; Krajangdara et al. 2022).

####### Distribution.

Cosmopolitan in tropical and subtropical seas, but also reported from estuaries and rivers, tolerant of freshwater conditions (Compagno 1984).

####### Record in Thailand.

Gulf of Thailand and Andaman Sea (Compagno 1984; Krajangdara et al. 2022).

####### Taxonomic remarks and comparisons.

Teeth of *C.amboiensis* are very similar to those of *C.leucas* so that it is very difficult to differentiate them (Kocsis et al. 2019). As a result, ten incomplete teeth may belong either to *C.leucas* or *C.amboiensis*: CUF-NKNY-S2-1, CUF-NKNY-S3-9, CUF-NKNY-S5-1, CUF-NKNY-S5-5, CUF-NKNY-SA-6, CUF-NKNY-SB-2, CUF-NKNY-SC-10, CUF-NKNY-SD-7, CUF-NKNY-SE-3 and CUF-NKNY-SE-9.

###### ﻿ Carcharhinus cf. sorrah

Taxon classificationAnimaliaCarcharhiniformesCarcharhinidae

(Valenciennes, 1839)

3DD7E5A2-F581-585F-8F44-409AF7A277F5

Fig. 20

cf.Carcharias (Prionodon) sorrah Valenciennes in Müller & Henle, 1839: 45–46, pl. 16. Type locality: Java.
cf. Carcharhinus sorrah . Compagno 1984: 500–501, with in-text figs. Krajangdara et al. 2022: 57, with in-text figs.

####### Referred material.

CUF-NKNY-3.1 (Fig. 20A–G), CUF-NKNY-3.2 (Fig. 20H–N) (2 upper teeth).

####### Description.

The crown is compressed labio-lingually. The labial face is slightly flatter than the lingual one. The cusp is inclined distally and presents a notch on its distal part. The serrations do not reach the apex of the main cusp and are enlarged, becoming more complex basally, especially on the distal heel. The mesial edge of the crown is almost straight in labial or lingual view. The base of the root is slightly concave in lingual or labial view. There is a well-developed groove with a nutritive foramen at the base of the root in lingual view.

####### Habitat.

Coastal, shallow-water zones of the continental and insular shelves, primarily around coral reefs at intertidal zones down to 73 m depth (Compagno 1984; Krajangdara et al. 2022).

####### Distribution.

Red Sea and Indian Ocean; Indo-West Pacific, from China to Australia (Compagno 1984).

####### Record in Thailand.

Gulf of Thailand and Andaman Sea (Compagno 1984; Krajangdara et al. 2022).

####### Taxonomic remarks and comparisons.

Some upper teeth of *C.sorrah* appear to display a mesial cutting edge slightly more convex than on our specimens, but this character is known to depend on ontogenetic stages and the position of the teeth in the jaw (e.g., Bass et al. 1973: pl. 13; Garrick 1982: fig. 76). Juveniles of *C.dussumieri* may have upper teeth with similar characteristics, but the distal cutting edge of the main cusp is not serrated. This latter character appears in larger specimens, but the mesial cutting edge of the main cusp becomes slightly concave. In addition, the distal heels and the distal root lobe of the fossil teeth are more elongated than the upper teeth of *C.dussumieri* (White 2012).

###### ﻿ Carcharhinus spp.

Taxon classificationAnimaliaCarcharhiniformesCarcharhinidae

139C1F54-2D6C-59FE-9455-E0F1356CD1BC

Figs 21 , 22

####### Referred material.

CUF-NKNY-16.1 (Fig. 21A–G), CUF-NKNY-S2 (Fig. 21H–L), CUF-NKNY-S3-4, CUF-NKNY-S3-8, CUF-NKNY-S4-7 (5 lower teeth), CUF-NKNY-S1 (Fig. 22A–H), CUF-NKNY-S3-7 (two upper teeth).

####### Description.

The specimens CUF-NKNY-16.1, CUF-NKNY-S3-4, CUF-NKNY-S3-8, and CUF-NKNY-S4-7 display a narrow main cusp that is inclined lingually. Their labial face is flatter than the lingual one. The serrations are well-developed in the upper two-third of the cusp. The serrations appear to be very fine on the mesial and distal heels in CUF-NKNY-16.1, CUF-NKNY-S3-4, and CUF-NKNY-S3-8, but the serrations are damaged in CUF-NKNY-S4-7. The root is well preserved only in CUF-NKNY-S3-4 and CUF-NKNY-S3-8, both of which display a slightly arched base, a moderately bulged lingual face with a nutritive foramen in its centre in CUF-NKNY-S3-4 and a faint nutritive groove in CUF-NKNY-S3-4. The specimen CUF-NKNY-S2 is asymmetric, with a mesial heel almost twice as elongated as the distal one and displays a sigmoid main cusp in labial or lingual view as well as in mesial or distal view, separated from the distal heel by a notch. The heels are serrated, but the main cusp is smooth. This tooth appears quite bulbous in mesial or distal view, being less compressed labio-lingually than the specimens described above. There is a well-developed nutritive groove on the root in lingual view, forming a basal notch in labial view.

A heavily worn tooth (CUF-NKNY-S3-7) is better attributed to an upper tooth. The lingual and distal faces of the crown are strongly damaged and a part of the distal end of the root is missing. The serration is only preserved in the middle part of the mesial cutting edge and appears to be quite regular. The root has a basal face slightly arched with a well-developed nutritive groove on its lingual face, forming a notch at the base of the root in labial view.

Another upper tooth, CUF-NKNY-S1, displays a crown with a quite coarse, irregular serration pattern nearly reaching the apex of the main cusp. The latter is bent lingually and slightly inclined distally. Its root shows a well-developed nutritive groove in lingual view, forming a notch at the base of the root in labial view. Apart from this notch, the base is almost straight.

####### Taxonomic remarks and comparisons.

Lower teeth of *C.amblyrhynchos* (*C.wheeleri* in Garrick (1982): fig 51) display features similar to the specimen CUF-NKNY-16.1. They are characterised by a narrow main cusp that displays serrations ending at mid-height. Some of the lower teeth of *C.longimanus* also have these morphological patterns, though their crown is often more robust and wider at their base (Bass et al. 1973: pl. 17; Garrick 1982: fig. 70). Nevertheless, teeth very similar to the specimens CUF-NKNY-16.1, CUF-NKNY-S3-4, CUF-NKNY-S3-8, and CUF-NKNY-S4-7 from the Pliocene of Italy were attributed to *C.longimanus* (Marsili 2007).

The sigmoid crown of the specimen CUF-NKNY-S2 is reminiscent of the posterior lower teeth of *C.borneensis* and *C.tjutjot* (Voigt and Weber 2011). The dentition attributed to *C.dussumieri* by Voigt and Weber (2011) belongs in fact to *C.tjutjot* (White 2012). A sigmoid cusp, when present, is less developed in *C.sealei* and *C.sorrah* (Voigt and Weber 2011), and the distal heel is coarsely serrated in *C.coatesi* (White 2012). The morphology of the crown recalls that of *Rhizoprionodon*, but in the latter genus, the distal heel is often more convex than in the specimen CUF-NKNY-S2 (Cappetta 2012: fig. 283; Carrillo-Briceño et al. 2015). This set of lower teeth is therefore attributed to the genus *Carcharhinus* but represents more than a single species, whereas the specimen CUF-NKNY-S3-7 is poorly preserved. Moreover, the posterior part is only preserved in the specimen CUF-NKNY-S1 and this does not allow us to reach the species-level identification.

There are 27 species of the genus *Carcharhinus* reported from the Southeast Asian region (Froese and Pauly 2024), and at least 20 taxa were recorded in Thai waters (Krajangdara et al. 2022) including all the ones mentioned above.

***Glyphis* Agassiz, 1843**

###### ﻿ Glyphis

Taxon classificationAnimaliaCarcharhiniformesCarcharhinidae

sp.

44784C07-4B7A-539A-9DC9-DFD6F86D17CD

**Figure 25.**
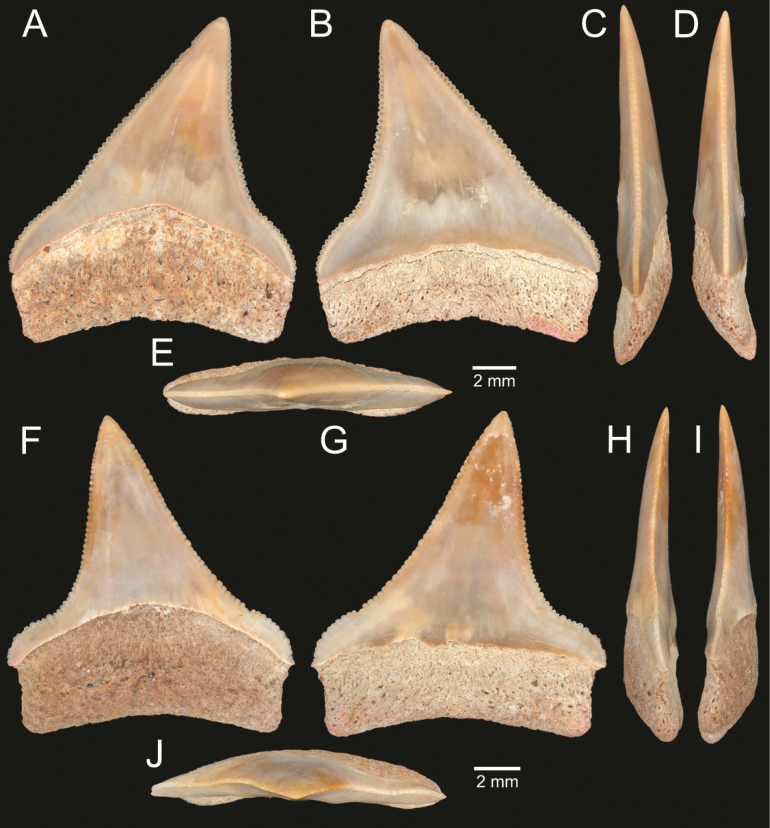
Carcharhinid shark teeth - *Glyphis* sp. **A–E** specimen CUF-NKNY-SC-3, upper anterolateral tooth in **A** lingual **B** labial **C** mesial **D** distal and **E** apical views **F–J** specimen CUF-NKNY-SD-3, upper lateral tooth in **F** lingual **G** labial **H** mesial **I** distal and **J** apical views.

**Figure 26.**
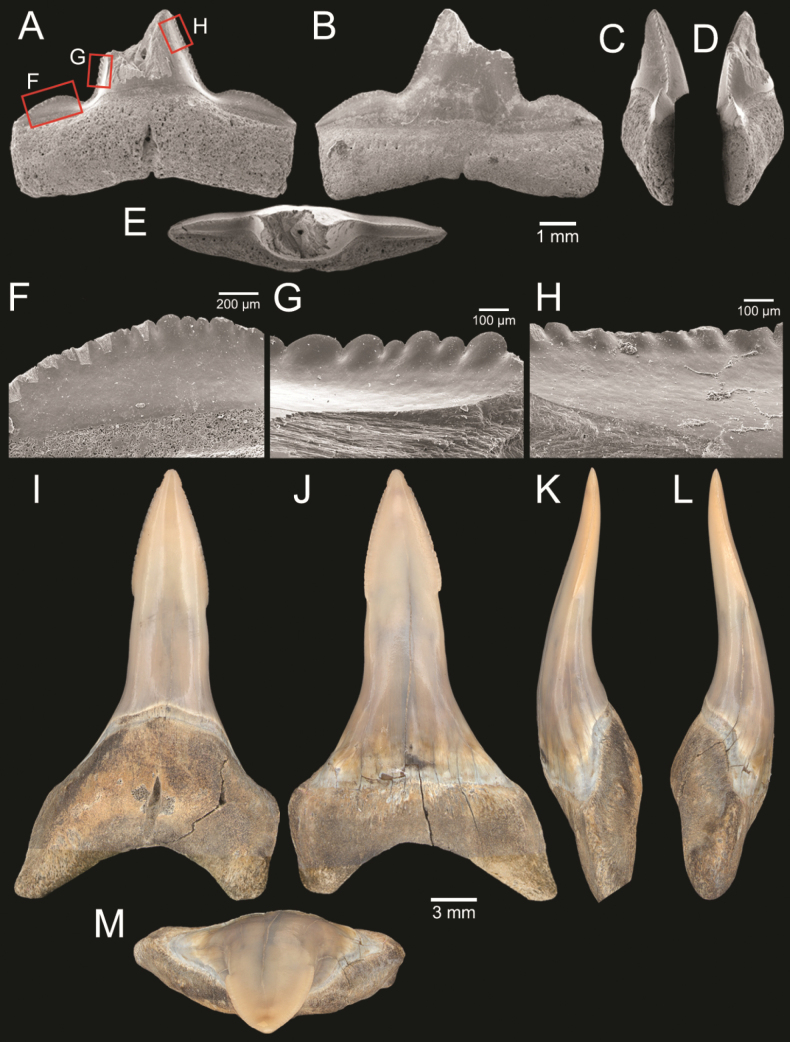
Carcharhinid shark teeth - *Glyphis* sp. **A–H** specimen CUF-NKNY-14, lower tooth in **A** lingual **B** labial **C** mesial **D** distal and **E** apical views **F–H** close-ups on the serration **F** on the distal heel, **G** at the base of the cusp distal cutting-edge and **H** in the middle part of the cusp mesial cutting-edge **I–M** specimen CUF-NKNY-S1-2, lower anterior tooth in **I** lingual **J** labial **K** mesial **L** distal and **M** apical views.

**Figure 27.**
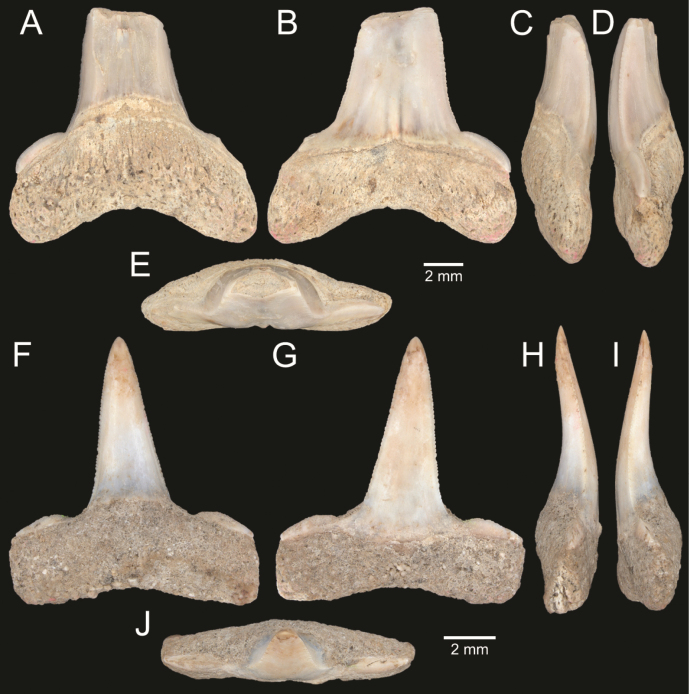
Carcharhinid shark teeth - *Glyphis* sp. **A–E** specimen CUF-NKNY-S3-1, lower tooth in **A** lingual **B** labial **C** mesial **D** distal and **E** apical views **F–J** specimen CUF-NKNY-S3-6, lower tooth in **F** lingual **G** labial **H** mesial **I** distal and **J** apical views.

**Figure 28.**
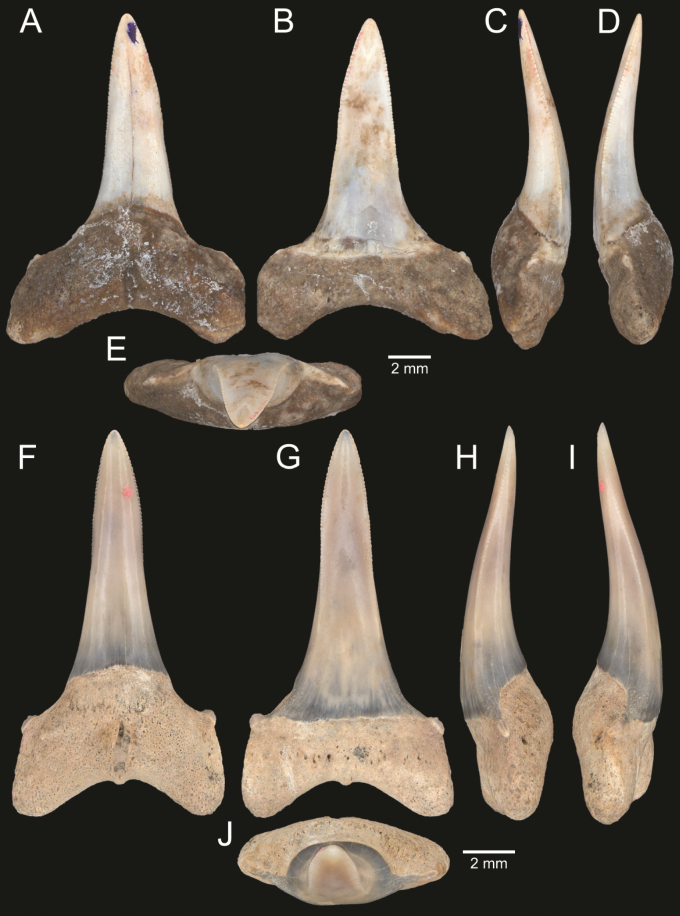
Carcharhinid shark teeth - *Glyphis* sp. **A–E** specimen CUF-NKNY-S4-1, lower tooth in **A** lingual **B** labial **C** mesial **D** distal and **E** apical views **F–J** specimen CUF-NKNY-S4-4, lower anterior tooth in **F** lingual **G** labial **H** mesial **I** distal and **J** apical views.

Figs 23 , 24 , 25 , 26 , 27 , 28

####### Referred material.

CUF-NKNY-8.2 (Fig. 23A–H), CUF-NKNY-Q03 (Fig. 24A–H), CUF-NKNY-S5-2, CUF-NKNY-S5-4, CUF-NKNY-S5-6, CUF-NKNY-S5-7, CUF-NKNY-SA-2 to 5, CUF-NKNY-SA-7, CUF-NKNY-SA-8, CUF-NKNY-SB-1, CUF-NKNY-SB-4 to 9, CUF-NKNY-SC-1, CUF-NKNY-SC-3 (Fig. 25A–E), CUF-NKNY-SC-4, CUF-NKNY-SC-6, CUF-NKNY-SC-8, CUF-NKNY-SC-9, CUF-NKNY-SD1 to 3 (Fig. 25F–J), CUF-NKNY-SD6, CUF-NKNY-SD-8 to 10, CUF-NKNY-SE1 to 2, CUF-NKNY-SE-4 to 7, CUF-NKNY-SE-10 to 15, CUF-NKNY-SE-17 to 18 (46 upper teeth), CUF-NKNY-14 (Fig. 26A–H), CUF-NKNY-S1-1 to 4 (Fig. 26I–M), CUF-NKNY-S3-1 (Fig. 27A–E), CUF-NKNY-S3-5 to 6 (Fig. 27F–J), CUF-NKNY-S4-1 to 2 (Fig. 28A–E), CUF-NKNY-S4-4 to 6 (Fig. 28F–J) (13 lower teeth).

####### Description.

Many teeth are poorly preserved. The crown of the upper teeth is triangular in outline, with a concavity at the base on both the mesial and distal sides in labial and lingual view, although the distal one is better marked, especially in anterior teeth (CUF-NKNY-S5-7). The serrations are quite fine and homogeneous in size along the cutting edges. The crown is strongly compressed labio-lingually and may be slightly curved lingually. When being broken, the crown displays a pulp cavity compressed labio-lingually, indicating an orthodont tooth histology (CUF-NKNY-SE-11). The base of the root is slightly concave in labial or lingual view. A well-developed nutritive groove and/or a nutritive foramen may be present on the lower half of the lingual side of the root.

The lower teeth may reach 40 mm in height. They are narrower and less compressed labio-lingually than the upper ones. The enameloid is smooth and the labial face of the crown is flatter than the lingual one. The main cusp is inclined lingually, sometimes with a sigmoid outline (CUF-NKNY-S4-2, CUF-NKNY-S4-5). Three different crown morphologies are noted. (1) On the specimens CUF-NKNY-S1-1, CUF-NKNY-S1-2, and CUF-NKNY-S1-4, the crown is devoid of heels and the tip of the main cusp is spearhead-shaped, the latter being regularly serrated, whereas the rest of the crown is smooth. (2) The specimens CUF-NKNY-S4-1 and CUF-NKNY-S4-4 have a narrow and upright triangular main cusp devoid of serration, showing a tiny accessory cusplet on each side of the main cusp in CUF-NKNY-S4-4 or only lingually in CUF-NKNY-S4-1. (3) On the specimen CUF-NKNY-S3-6, the main cusp is also upright, narrow, triangular in shape, and serrated on their mesial and distal edges. Well-developed heels are present on each side of the main cusp, separated from the latter by a notch. The heel is sometimes less marked on the mesial side (CUF-NKNY-S3-1). The heels display less developed serration than on the main cusp. Many teeth belonging to this morphotype display a broken apex of the main cusp (CUF-NKNY-14).

The root displays a bulged lingual face. It is more massive and more arched in spearhead-shaped teeth than in heeled ones. There is a nutritive groove in the lingual side of the root with a nutritive foramen in its middle part that can be observed in most of the teeth. The specimen CUF-NKNY-S1-1 displays a double groove.

####### Taxonomic remarks and comparisons.

Upper anterior teeth (CUF-NKNY-Q03) are more erect and narrower than the lateral ones (CUF-NKNY-8.2). Lower teeth with a spearhead-shaped apex correspond to the larger teeth in our sample, in agreement with the fact that this morphology is known only in anterior teeth of adult specimens of *Glyphisglyphis* and *G.garricki*. However, lateral teeth of these species display serrated heels and teeth of juveniles can display a pair of tiny lateral cusplets (Compagno et al. 2008; White et al. 2015). As a result, the three lower tooth morphotypes described above could be encountered into a single species during its ontogeny. Otherwise, it could indicate the presence of more than one species, possibly three: *G.glyphis*, *G.garricki*, and *G.gangeticus*, as the latter species often possesses anterior lower teeth with tiny lateral cusplets but lacks the spearhead-shaped apex (Roberts 2006; Cappetta 2012). Consequently, the teeth described above are left in open nomenclature as *Glyphis* sp. This taxon appears to be most common in the studied fauna, although the discrepancies between the number of upper and lower teeth recovered suggest some degree of collecting bias.

The genus is specific to the Indo-West Pacific tropical region and often referred as “river shark” due to its habitat in or nearby rivers and estuaries. They are quite rare, hence difficult to study. Five living species are known (Froese and Pauly 2024), but DNA analyses suggest that some of these are conspecific (Li et al. 2015). On the other hand, teeth of *Glyphis* are often recorded from Southeast Asia (Shimada et al. 2016; Kocsis et al. 2019 and references therein).

***Scoliodon* Müller & Henle, 1837**

###### ﻿ Scoliodon cf. laticaudus

Taxon classificationAnimaliaCarcharhiniformesCarcharhinidae

Müller & Henle, 1838

6A68C781-AADF-5C3F-9FB3-048E6C15F303

Fig. 29

cf.Carcharias (Scoliodon) laticaudus Müller & Henle, 1838: 28–29, pl. 8. Type locality: India.
cf. Scoliodon laticaudus . Compagno 1984: 534–535, with in-text figs. Krajangdara et al. 2022: 62, with in-text figs.

####### Referred material.

**Figure 29.**
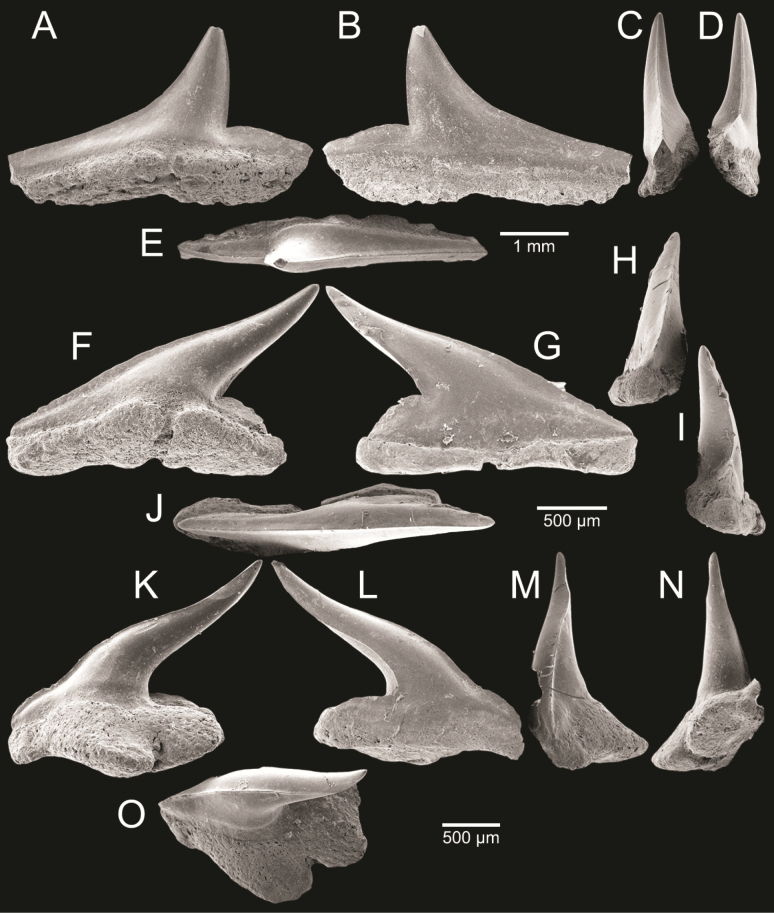
Carcharhinid shark teeth - Scoliodoncf.laticaudus**A–E** specimen CUF-NKNY-S3, lower anterolateral tooth in **A** lingual **B** labial **C** mesial **D** distal and **E** apical views **F–J** specimen CUF-NKNY-S4, upper anterior-anterolateral tooth in **F** lingual **G** labial **H** mesial **I** distal and **J** apical views **K–O** specimen CUF-NKNY-S5, parasymphyseal tooth in **K** lingual **L** labial **M** mesial **N** distal and **O** apical views.

CUF-NKNY-S3–S5 (Fig. 29A–O) (3 teeth).

####### Description.

The specimen CUF-NKNY-S3 is a small and elongated tooth with a low crown and a worn off root. The mesial cutting edge is concave and smooth and continues along the mesial heel. The distal edge of the crown is convex and separated by a notch from a distinct distal heel.

The specimen CUF-NKNY-S4 is a well-preserved tooth, with a crown strongly curved distally. The mesial cutting edge is sinusoid, starting convex, then becoming concave towards the apex. The apex of the crown overhangs distally the basal part of the tooth. The distal cutting edge is also sinusoid, though less so than the mesial one, and it is separated by a distinct notch from a well-developed distal heel. The enameloid of the crown extends more basally on the labial side of the tooth. The root is thick and the lobes run rather horizontally. It bears a well-developed and deep nutritive groove in the centre.

The cusp of the specimen CUF-NKNY-S5 is strongly sigmoid in labial and lingual view, and similar to that of CUF-NKNY-S4, but its cusp is narrower and less wide at the base, with more apparent mesial heels. The cutting edge is smooth and does not reach the apex of the cusp on its mesial side. The heels of the crown display faint serrations. The root is asymmetric and projected lingually and distally. There is a well-defined groove distally on the lingual side of the basal face, forming a deep notch in apical view.

####### Habitat.

Tropical zones of continental and insular shelves close to inshore, frequently in rocky areas (Compagno 1984).

####### Distribution.

Indian Ocean; Indo-West Pacific, from Japan to Indonesia (Compagno 1984).

####### Record in Thailand.

Andaman Sea (Krajangdara et al. 2022).

####### Taxonomic remarks and comparisons.

The three teeth best fit the dentition of the modern species *S.laticaudus* (spadenose shark). The specimen CUF-NKNY-S5 has a slender sigmoidal cusp and its mesial cutting edge does not reach the apex. It displays an asymmetric root, corresponding perfectly to a parasymphyseal tooth of a male specimen (Herman et al. 1991). The specimen CUF-NKNY-S4 likely represents an upper anterior-anterolateral tooth, whereas the specimen CUF-NKNY-S3 is probably a lower anterolateral tooth (see Springer 1964: fig. 3a). White et al. (2010) recognised another species, *S.macrorhynchos*, in the Western Pacific, meanwhile a molecular study also indicates the possible third *Scoliodon* species from the region (Lim et al. 2022), hence these fossil teeth are described in open nomenclature.

It may be mentioned that the specimen CUF-NKNY-S3 also resembles that of the lower anterolateral teeth of other common sharks in Southeast Asia (Krajangdara et al. 2022) such as *Loxodonmacrorhinus* (sliteye shark) and two *Rhizoprionodon* species, *R.acutus* (milk shark) and *R.oligolinx* (grey sharpnose shark). For the teeth of *Loxodon*, the apex of the crown is higher and somewhat aligned in a more distal position than that of the specimen CUF-NKNY-S3 (Springer 1964: fig. 4; Bass et al. 1975: pl. 8). The teeth of *R.oligolinx* seem to have a less concave mesial cutting edge (Springer 1964: fig. 13), whereas for *R.acutus*, the distal heel appears less elongated and vertically higher, but a clear distinction is difficult here (Springer 1964: fig. 6; Bass et al. 1975: pl. 9).

###### ﻿ Carcharhinidae

Taxon classificationAnimaliaCarcharhiniformesCarcharhinidae

indet.

F043912A-7B9B-5DEE-8F6D-472D24770C0B

####### Referred material.

CUF-NKNY-S3-3, CUF-NKNY-S4-3, CUF-NKNY-S5-3, CUF-NKNY-SC-2, CUF-NKNY-SC-7, CUF-NKNY-SD-4, CUF-NKNY-SD-5, CUF-NKNY-SD-11, CUF-NKNY-SE-8, CUF-NKNY-SE-16 (10 teeth).

####### Description.

These teeth are poorly preserved. The crown is generally triangular, more or less narrow, and symmetric with serration incompletely preserved. The root is broken away or does not display the details of its vascularisation pattern.

####### Taxonomic remarks and comparisons.

The presence of triangular serrated crowns suggests that most of the teeth belong to upper ones of *Carcharhinus* and/or *Glyphis*, but their poor preservation does not allow a more precise identification. The specimen CUF-NKNY-SC-7 may represent a lower tooth of *C.leucas*/*C.amboinensis*, but without the root and the base of the crown preserved, this is impossible to ascertain.

**Superorder Batomorphii Capetta, 1980**

**Order Myliobatiformes Compagno, 1973**

##### ﻿Family Dasyatidae Jordan & Gilbert, 1879

***Pastinachus* Rüppell, 1829**

###### ﻿ Pastinachus

Taxon classificationAnimaliaMyliobatiformesDasyatidae

sp.

89ADBEF9-2496-5A4D-9E1A-B1E0666E8170

Fig. 30

####### Referred material.

**Figure 30.**
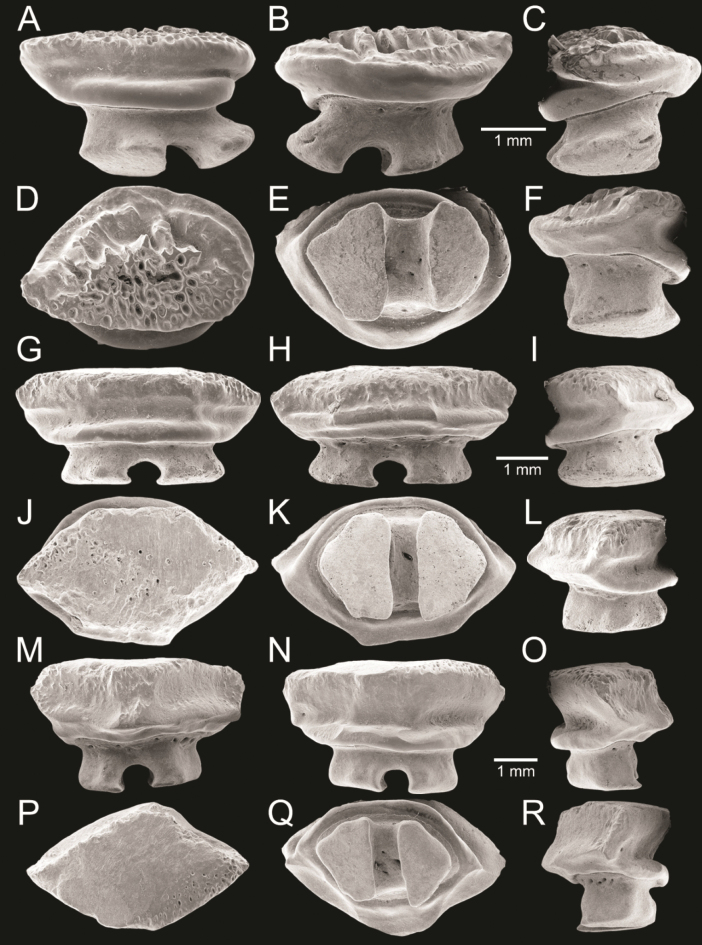
Dasyatid ray teeth - *Pastinachus* sp. **A–F** specimen CUF-NKNY-7.1 in **A** lingual **B** labial **C, F** profile **D** occlusal and **E** basal views **G–L** specimen CUF-NKNY-7.2 in **G** lingual **H** labial **I, L** profile **J** occlusal and **K** basal views **M–R** specimen CUF-NKNY-8.1 in **M** lingual **N** labial **O, R** profile **P** occlusal and **Q** basal views.

CUF-NKNY-7.1 (Fig. 30A–F), CUF-NKNY-7.2 (Fig. 30G–L), CUF-NKNY-8.1 (Fig. 30M–R) (3 teeth).

####### Description.

The crown is hexagonal to diamond-shaped in apical view, longer mesio-distally than labio-lingually. The crown surface is rather smooth to heavily pitted. The labial face of the crown displays a salient horizontal bulge. There is a well-developed horizontal groove in the basal part of the crown on the lingual face. The vascularisation of the teeth is holaulacorhize. There is a row of small foramina positioned under the crown on the labial face and between one and four foramina present in the groove separating the two branches of the root in basal view.

####### Taxonomic remarks and comparisons.

Heavily pitted crowns probably belong to non-functional teeth (Adnet et al. 2019). Four species of cowtail rays (*Pastinachusater*, *P.gracilicaudus*, *P.solocirostris*, and *P.stellurostris*) are known in Southeast Asia (Last et al. 2016), all of them having been recorded in Thai waters (Krajangdara et al. 2022). Regarding the nearby fossil record, teeth of *Pastinachus* were reported from India, Taiwan, and Borneo (see discussion in Kocsis et al. 2019).

﻿**Class Actinopterygii Klein, 1885**

**Infraclass Teleostei Müller, 1845**

**Order Scombriformes Rafinesque, 1810**

##### ﻿Family Trichiuridae Rafinesque, 1810

###### ﻿ Trichiuridae

Taxon classificationAnimaliaScombriformesTrichiuridae

indet.

7B9D026A-2B9A-5EDD-A115-F25D529DD433

Fig. 31

####### Referred material.

**Figure 31.**
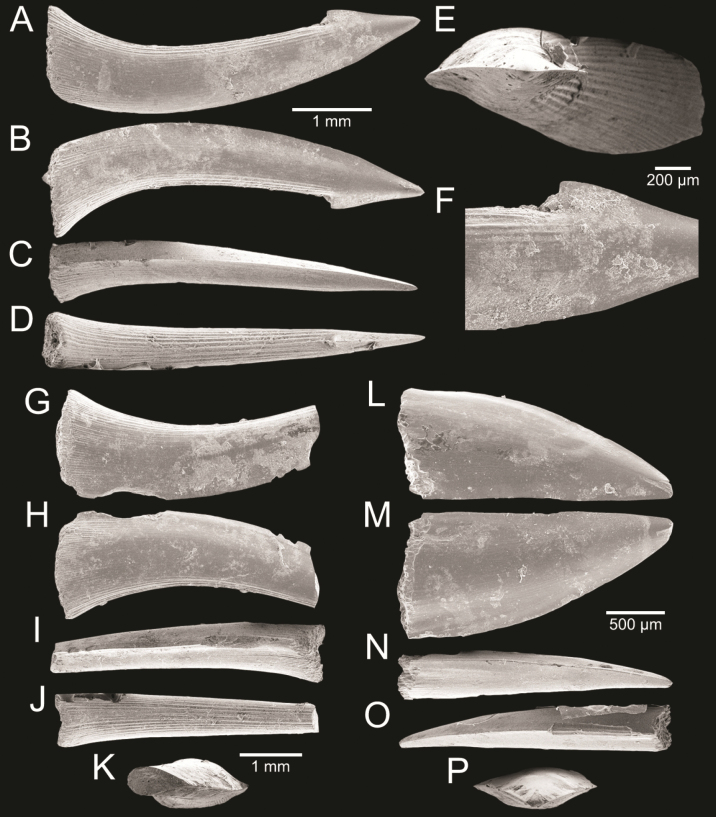
Trichiuridae indet. **A–F** specimen CUF-NKNY-2.1, anterior tooth in **A, B** lateral **C** anterior **D** posterior and **E** apical views **F** close-up of the tip of the tooth **G–K** specimen CUF-NKNY-2.2, anterior tooth in **G, H** lateral **I** anterior **J** posterior and **K** apical views **L–P** specimen CUF-NKNY-18.2, lateral tooth in **L, M** lateral **N** anterior **O** posterior and **P** apical views.

CUF-NKNY-2.1 (Fig. 31A–F), CUF-NKNY-2.2 (Fig. 31G–K), CUF-NKNY-18.2 (Fig. 31L–P) (3 teeth).

####### Description.

Labiolingually flattened teeth seem to thin out at their mesial edge. They are covered with elongated and fine striation. This striation becomes coarser basally and distally on the more elongated specimens (specimens CUF-NKNY-2.1 and CUF-NKNY-2.2). The latter two teeth are strongly curved distally with a concave distal edge and the more completely preserved specimen CUF-NKNY-2.1 bears an apical barb. The specimen CUF-NKNY-18.2 has a curved mesial margin, whereas the distal one is vertical and straight.

####### Taxonomic remarks and comparisons.

The two strongly curved teeth (specimens CUF-NKNY-2.1 and CUF-NKNY-2.2) represent fang-like features from the front of the jaw, while the specimen CUF-NKNY-18.2 comes from a rather distal position. This latter tooth resembles somewhat the lateral teeth of *Sphyranea* (barracuda, e.g., Gottfried et al. 2017), but those are more symmetrical. The global species database (Fishbase: www.fishbase.org) reports twenty-seven cutlassfishes from the wider region of South and Southeast Asia (Froese and Pauly 2024). Two common genera, *Trichurus* and *Lepuracanthus*, contain some species that bear such barbed fang-like teeth (Nakamura and Parin 1993; 1998).

**Order *incertae sedis* in Eupercaria Betancur-R. et al., 2014**

##### ﻿Family Sciaenidae Cuvier, 1829

***Johnius* Bloch, 1793**

###### ﻿ Johnius

Taxon classificationAnimaliaPerciformesSciaenidae

sp.

FD917AFD-4178-5F62-A287-D96F36C9D1B6

Fig. 32

####### Referred material.

**Figure 32.**
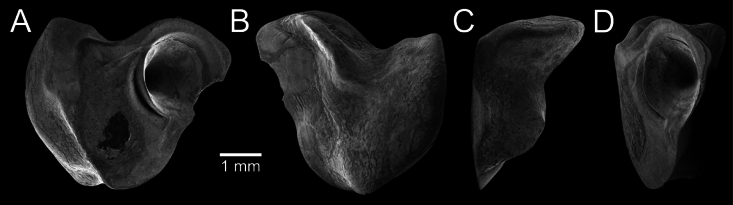
Sciaenid otolith of *Johnius* sp., specimen CUF-NKNY-16.2, right sagitta otolith in **A** inner **B** external **C** anterior and **D** posterior views.

CUF-NKNY-16.2 (Fig. 32A–D) (1 right sagitta otolith).

####### Description.

Thick otolith with a smooth external surface, whereas the internal surface bears a sulcus characteristic of the genus. The ostium is shallow, vertically positioned, higher than long, and it widens ventrally. The anterior and horizontal portion of the cauda is short and shallow, then opening posteriorly into a deep caudal funnel with an external circular shape.

####### Taxonomic remarks and comparisons.

Otoliths of *Johnius* are unique and have distinct characteristics that make them easily distinguishable from those of other sciaenids or teleosts (Schwarzhans 1993). There are 21 species of *Johnius* in Southeast Asia (Froese and Pauly 2024), and most of them inhabit shallow coastal waters and estuaries but some species are capable of entering rivers. They are also known from the fossil records in Japan (Ohe 1976) and Taiwan (Lin et al. 2022).

## ﻿﻿Discussion

Based on the survey of mid-Holocene marine faunas from the clay pit of Nakhon Nayok Province in Thailand, many fossils of molluscs together with other invertebrates and vertebrates have been found in the Bangkok clay layer at a depth of ~ 5–7 m below the surface. The faunal assemblages, especially molluscs (Table 2), depict the palaeo-ecosystem of the area at that time as corresponding to the intertidal to sublittoral zones. Some molluscan taxa, i.e., *Cerithideaobtusa*, *Pirenellaincisa*, *Telescopiumtelescopium*, *Ellobiumaurisjudae*, *Cassidulanucleus*, and *Geloinabengalensis*, also indicate that some parts of the area corresponded to mangrove forests and intertidal mudflats. The occurrence of mangrove forests has previously been corroborated by evidence of peat and pollens from the same, as well as nearby, sites (Songtham et al. 2007, 2015). In this study, several described mollusc taxa correspond to those reported from the Lower Central Plain of Bangkok and Samut Prakan and from the inland of Phetchaburi coasts (Somboon 1988; Somboon and Thiramongkol 1992; Robba et al. 2007; Negri 2009, 2012; Surakiatchai et al. 2018), suggesting similar paleoenvironments of these sites.

**Table 2.** Molluscan species assemblages from the Bangkok Clay deposits of Ongkharak in Nakhon Nayok recovered in the present study and from other sites retrieved from previous literature (indicated by an asterisk). NA = data not available.

Class	Species in this study	Lower Central Plain and Chao Phraya delta (Somboon 1988; Somboon and Thiramongkol 1992)	Lower Central Plain of Bangkok and inland of Phetchaburi coast (Robba et al. 2007; Negri 2009)	Ban Praksa, Samut Prakan (Negri 2012)	Sam Roi Yot National Park, Phetchaburi (Surakiatchai et al. 2018)	Habitat	Substrate preference
Gastropoda	1. Homalopomacf.sangarense	–	–	–	–	Sublittoral	Sand, hard
	2. *Neripteronviolaceum*	–	–	–	–	Intertidal, mangrove	Mud, hard
	3. *Neritaarticulata*	*	–	–	–	Intertidal, mangrove	Mud, hard
	4. *Cerithideaobtusa*	–	*	–	–	Intertidal, mangrove	Mud, hard
	5. *Pirenellaincisa*	–	–	–	–	Intertidal, mangrove	Mud
	6. *Telescopiumtelescopium*	–	–	–	–	Intertidal, mangrove	Mud
	7. *Eunaticinapapilla*	–	*	–	–	Intertidal–sublittoral	Sand
	8. *Naticastellata*	–	*	–	–	Sublittoral	Sand
	9. *Naticavitellus*	–	*	–	–	Intertidal– sublittoral	Sand, mud
	10. *Paratectonaticatigrina*	*	*	*	*	Intertidal– sublittoral	Sand, mud
	11. *Ergaeawalshi*	–	*	–	–	Intertidal– sublittoral	Hard
	12. *Bufonariarana*	–	–	–	–	Sublittoral	Sand, mud
	13. *Mericaelegans*	–	–	–	–	Sublittoral	Sand, mud
	14. *Scalptiascalariformis*	–	*	–	–	Sublittoral	Sand, mud
	15. *Pseudoneptuneavaricosa*	–	*	–	–	Sublittoral	NA
	16. *Brunneifususternatanus*	*	–	–	–	Sublittoral	Sand, mud
	17. *Nassariusmicans*	–	*	–	–	Intertidal– sublittoral	Sand
Gastropoda	18. *Nassariussiquijorensis*	–	*	–	*	Intertidal– sublittoral	Sand, mud
	19. *Chicoreuscapucinus*	*	–	–	–	Intertidal, mangrove	Sand, mud, hard
	20. *Indothaisgradata*	*	–	–	–	Sublittoral	Hard
	21. *Indothaislacera*	–	*	–	–	Intertidal– sublittoral	Mud, hard
	22. *Murextrapa*	*	*	–	*	Sublittoral	Sand, mud
	23. *Maoritomellavallata*	–	*	–	–	Sublittoral	NA
	24. *Pseudoetremafortilirata*	–	*	–	–	Sublittoral	Sand, mud
	25. *Turriculajavana*	*	*	–	*	Intertidal– sublittoral	Sand, mud, hard
	26. *Paradrilliamelvilli*	–	*	–	–	Sublittoral–upper bathyal	NA
	27. *Inquisitorvulpionis*	–	*	–	–	Sublittoral	Sand
	28. *Comitasilariae*	–	–	–	–	Sublittoral	Sand
	29. *Duplicariatricincta*	–	*	–	–	Sublittoral	Sand, mud
	30. *Granuliterebrabathyrhaphe*	–	*	*	–	Intertidal– sublittoral	Sand, mud
	31. *Pristiterebramiranda*	–	*	–	–	Sublittoral	NA
	32. *Architectonicaperdix*	–	*	–	*	Sublittoral	Sand, mud
	33. *Cylichnamodesta*	–	*	–	–	Sublittoral	Sand, mud
	34. *Ellobiumaurisjudae*	–	*	–	–	Intertidal, mangrove	Sand, mud, hard
	35. *Cassidulanucleus*	–	*	–	–	Intertidal, mangrove	Mud, hard
Bivalvia	1. *Jupiteriapuellata*	–	*	*	–	Sublittoral	Sand, mud
	2. *Saccellamauritiana*	–	*	–	–	Sublittoral	Sand, mud, hard
	3. *Anadarainaequivalvis*	–	*	–	*	Intertidal–sublittoral	Sand, mud
	4. *Anadaraindica*	–	*	–	*	Intertidal– sublittoral	Sand, mud
	5. *Tegillarcagranosa*	*	*	–	*	Intertidal, mangrove	Mud
	6. *Tegillarcanodifera*	–	–	–	–	Intertidal– sublittoral, mangrove	Sand, mud
	7. *Estellacarolivacea*	*	*	–	–	Intertidal– sublittoral, mangrove	Sand, mud
	8. *Noetiellapectunculiformis*	–	*	–	–	Intertidal– sublittoral, mangrove	Sand, mud
	9. Magallanacf.gigas	–	*	–	*	Intertidal– sublittoral	Mud, hard
	10. *Placunaplacenta*	–	*	*	*	Intertidal– sublittoral	Sand, mud
	11. *Volachlamyssingaporina*	–	–	–	–	Intertidal– sublittoral	Sand, mud, hard
	12. *Pegophysemabialata*	–	–	–	–	Intertidal– sublittoral	Sand, mud
	13. *Geloinabengalensis*	–	*	–	–	Intertidal, mangrove	Mud
	14. *Lutrariacomplanata*	–	*	–	–	Sublittoral	Sand, mud
	15. *Standellapellucida*	–	*	–	–	Intertidal– sublittoral, mangrove	Sand, mud
	16. *Tellinidesconspicuus*	–	–	–	–	Sublittoral	NA
	17. *Joannisiellaoblonga*	*	*	–	*	Intertidal– sublittoral	Mud
	18. *Dosiniadilecta*	–	*	–	*	Sublittoral	Mud
	19. *Paratapesundulatus*	*	*	*	*	Intertidal– sublittoral	Sand, mud
	20. *Placamenlamellatum*	–	*	–	–	Intertidal– sublittoral	Sand, mud
	21. *Corbulafortisulcata*	–	*	–	*	Intertidal–sublittoral	Sand, mud, hard
	22. *Potamocorbula* sp.	NA	NA	NA	NA	Intertidal	NA
	23. *Martesiastriata*	–	*	–	–	Intertidal–sublittoral	Hard
	24. *Pholasorientalis*	–	*	–	–	Intertidal–sublittoral	Sand, mud, hard
	25. Teredinidae indet.	NA	NA	NA	NA	Intertidal–sublittoral	Hard
	26. *Cultellusmaximus*	–	*	–	–	Intertidal–sublittoral, mangrove	Mud
	27. *Siliquaminima*	–	*	–	–	Intertidal–sublittoral	Sand, mud
Scaphopoda	1. *Dentaliumvariabile*	–	*	*	–	Sublittoral	Mud

**Figure 33.**
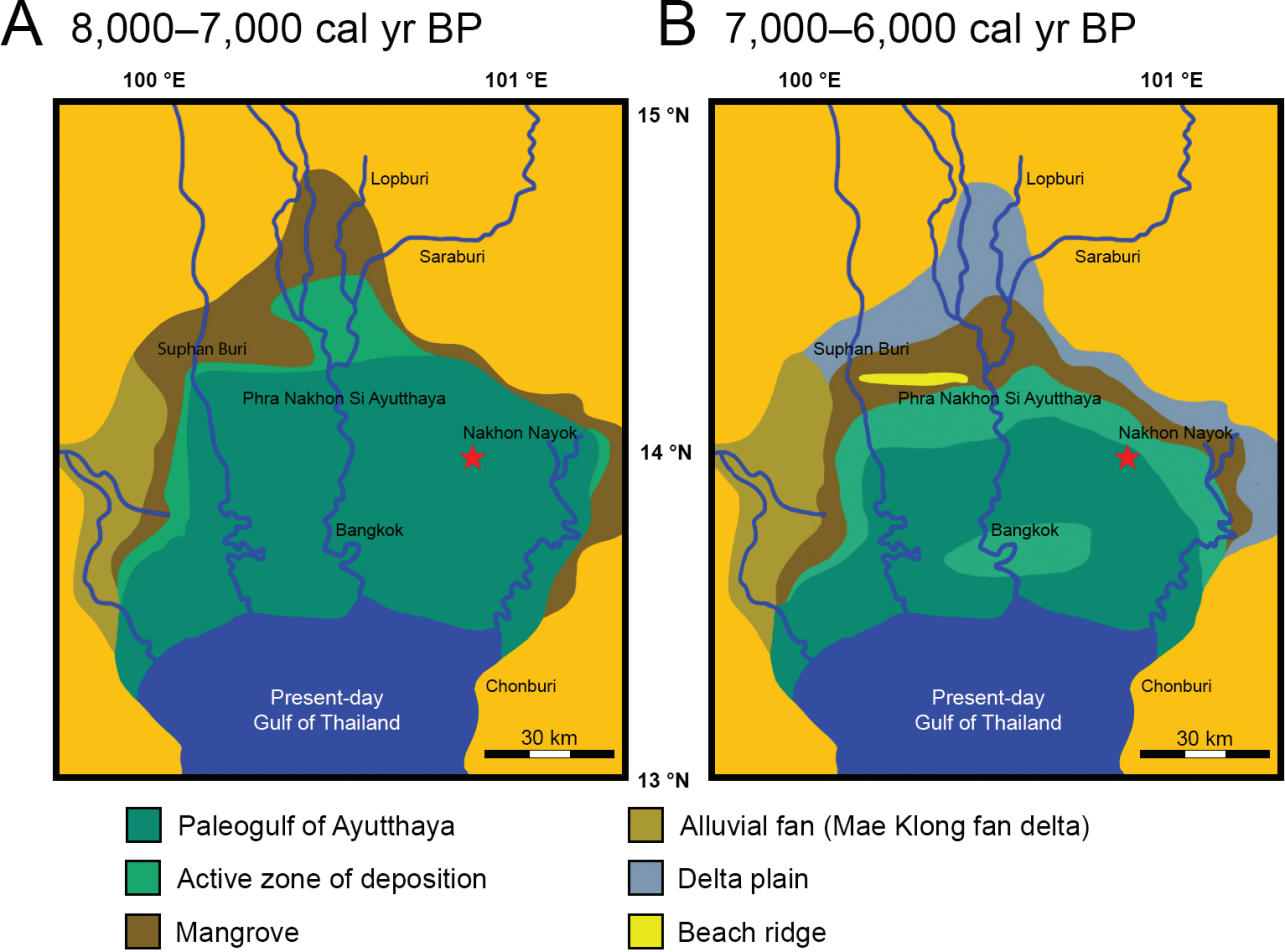
Paleogeographic maps illustrating the evolution of the Chao Phraya delta modified from Tanabe et al. (2003), during **A** 8,000–7,000 cal yr BP and **B** 7,000–6,000 cal yr BP. Red stars indicate the location of the study area.

Most of the marine and mangrove shells were found at a depth of 2.2 m below the uppermost part of a marine clay layer. The carbon-14 dating analysis indicates a mid-Holocene age for this level as being approximately 5,900–5,300 cal yr BP, whereas charcoal material was found at a greater depth of 2.2–4.6 m, spanning approximately an age from 8,800 to 5,700 cal yr BP (Table 1). However, the abundance of tree trunks and carbonised woods as well as the accumulation of peat at greater depth than most shells indicate the predominance of mangrove forests, suggesting an early stage of marine incursion further inland (Songtham et al. 2007). The ages of the Bangkok Clay deposits determined by this study are also in good agreement with those retrieved by Songtham et al. (2007). This duration corresponds to the depositional stage 2 (7,000–3,000 cal yr BP), a marine transgression time proposed by Tanabe et al. (2003). During this stage, a large mud shoal was formed around Samut Prakan Province and the Chao Phraya delta prograded toward the head of the palaeo-Gulf of Ayutthaya southward (Fig. 33; Tanabe et al. 2003). However, our chronological results suggest that the area of Ongkharak, Nakhon Nayok in central Thailand was possibly an ancient coastal shoreline between 7,900 and 5,300 cal yr BP, which was located further southward compared to the palaeogeographic scheme proposed by Tanabe et al. (2003).

The vertebrate fauna is dominated by cartilaginous fishes, among which the family Carcharhinidae is most common (Table 3). The genus *Glyphis*, also known as “river shark”, is dentally most abundant in the area due to its habitat in or nearby rivers and estuaries. It is a cryptic shark that is difficult to study, but it has been reported that these sharks also live in coastal and shallow marine regions, while the young grow up in a river habitat where the predation pressure is reduced (Li et al. 2015). The dominant presence of this taxon is indicative of the vicinity of freshwater input and/or habitats close to rivers, corresponding to the paleogeographic context of the region (Fig. 33) and the results of other studies from the Bangkok Clay (Robba et al. 2002, 2003, 2004, 2007; Songtham et al. 2007; Negri 2009, 2012). Several *Carcharhinus* species including *C.amblyrhynchoides*, *C.amblyrhinchos*, *C.leucas*, *C.amboinensis*, and *C.sorrah* were also found. As the position of the teeth in the jaws cannot be ascertained, other taxa could not be ruled out based on some similarities in tooth morphology. Nevertheless, the bull shark (*C.leucas*) appears to be abundant and is also known to live in freshwater environments. Moreover, the genus *Scoliodon* present in the area is reported from brackish environments (Riede 2004). The other recovered taxa, the stingray (*Pastinachus*), cutlassfish (Trichiuridae), and the sciaenid *Johnius* are all compatible with shallow marine coastal environments, as supported by the presence of molluscan faunas, although some species are reported from brackish environments. Due to the fact that the fish remains are almost entirely represented by isolated teeth and only one single otolith has been found so far (*Johnius*), the rarity of this type of remains could be the consequence of sampling biases and/or taphonomic processes. Therefore, targeting otoliths in future studies could shed more light on the bony fish fauna of the Bangkok Clay.

**Table 3.** Fish remains recovered from the Bangkok Clay deposits of Ongkharak in Nakhon Nayok.

**Class Chondrichthyes**				
**Order**	**Family**	**Species**	**No.**	**Material**
Carcharhiniformes	Carcharhinidae	Carcharhinuscf.amblyrhynchoides	1	tooth
		Carcharhinuscf.amblyrhynchos	1	tooth
		Carcharhinuscf.leucas	4	teeth
		*Carcharhinusleucas/Carcharhinusamboinensis*	10	teeth
		Carcharhinuscf.sorrah	2	teeth
		*Carcharhinus* spp.	7	teeth
		*Glyphis* sp.	59	teeth
		Scoliodoncf.laticaudus	3	teeth
		Carcharhinidae indet.	10	teeth
Myliobatiformes	Dasyatidae	*Pastinachus* sp.	3	teeth
**Class Actinopterygii**				
Scombriformes	Trichiuridae	Trichiuridae indet.	3	teeth
*incertae sedis* in Eupercaria	Sciaenidae	*Johnius* sp.	1	otolith

## Supplementary Material

XML Treatment for Oulangia cf. stokesiana

XML Treatment for Homalopoma cf. sangarense

XML Treatment for Neripteron violaceum

XML Treatment for Nerita articulata

XML Treatment for Cerithidea obtusa

XML Treatment for Pirenella incisa

XML Treatment for Telescopium telescopium

XML Treatment for Eunaticina papilla

XML Treatment for Natica stellata

XML Treatment for Natica vitellus

XML Treatment for Paratectonatica tigrina

XML Treatment for Ergaea walshi

XML Treatment for Bufonaria rana

XML Treatment for Merica elegans

XML Treatment for Scalptia scalariformis

XML Treatment for Pseudoneptunea varicosa

XML Treatment for Brunneifusus ternatanus

XML Treatment for Nassarius micans

XML Treatment for Nassarius siquijorensis

XML Treatment for Chicoreus capucinus

XML Treatment for Indothais gradata

XML Treatment for Indothais lacera

XML Treatment for Murex trapa

XML Treatment for Maoritomella vallata

XML Treatment for Pseudoetrema fortilirata

XML Treatment for Turricula javana

XML Treatment for Paradrillia melvilli

XML Treatment for Inquisitor vulpionis

XML Treatment for Comitas ilariae

XML Treatment for Duplicaria tricincta

XML Treatment for Granuliterebra bathyrhaphe

XML Treatment for Pristiterebra miranda

XML Treatment for Architectonica perdix

XML Treatment for Cylichna modesta

XML Treatment for Ellobium aurisjudae

XML Treatment for Cassidula nucleus

XML Treatment for Jupiteria puellata

XML Treatment for Saccella mauritiana

XML Treatment for Anadara inaequivalvis

XML Treatment for Anadara indica

XML Treatment for Tegillarca granosa

XML Treatment for Tegillarca nodifera

XML Treatment for Estellacar olivacea

XML Treatment for Noetiella pectunculiformis

XML Treatment for Magallana cf. gigas

XML Treatment for Placuna placenta

XML Treatment for Volachlamys singaporina

XML Treatment for Pegophysema bialata

XML Treatment for Geloina bengalensis

XML Treatment for Lutraria complanata

XML Treatment for Standella pellucida

XML Treatment for Tellinides conspicuus

XML Treatment for Joannisiella oblonga

XML Treatment for Dosinia dilecta

XML Treatment for Paratapes undulatus

XML Treatment for Placamen lamellatum

XML Treatment for Corbula fortisulcata

XML Treatment for Potamocorbula

XML Treatment for Martesia striata

XML Treatment for Pholas orientalis

XML Treatment for Teredinidae

XML Treatment for Cultellus maximus

XML Treatment for Siliqua minima

XML Treatment for Dentalium variabile

XML Treatment for Thalassina

XML Treatment for Fistulobalanus kondakovi

XML Treatment for Megabalanus cf. tintinnabulum

XML Treatment for Temnotrema siamense

XML Treatment for Carcharhinus cf. amblyrhynchoides

XML Treatment for Carcharhinus cf. amblyrhynchos

XML Treatment for Carcharhinus cf. leucas

XML Treatment for Carcharhinus cf. sorrah

XML Treatment for Carcharhinus

XML Treatment for Glyphis

XML Treatment for Scoliodon cf. laticaudus

XML Treatment for Carcharhinidae

XML Treatment for Pastinachus

XML Treatment for Trichiuridae

XML Treatment for Johnius

## ﻿﻿Additional information

### Author ORCIDs

Parin Jirapatrasilp https://orcid.org/0000-0002-5591-6724

Gilles Cuny https://orcid.org/0000-0001-7680-1697

László Kocsis https://orcid.org/0000-0003-4613-1850

Chirasak Sutcharit https://orcid.org/0000-0001-7670-9540

Kantapon Suraprasit https://orcid.org/0000-0002-3428-9549

### Data availability

All of the data that support the findings of this study are available in the main text.
